# Meroterpenoid Dimers from *Ganoderma* Mushrooms and Their Biological Activities Against Triple Negative Breast Cancer Cells

**DOI:** 10.3389/fchem.2022.888371

**Published:** 2022-05-03

**Authors:** Fu-Ying Qin, Yan-Yi Chen, Jiao-Jiao Zhang, Yong-Xian Cheng

**Affiliations:** ^1^ Institute for Inheritance-Based Innovation of Chinese Medicine, School of Pharmaceutical Sciences, Health Science Center, Shenzhen University, Shenzhen, China; ^2^ Guangdong Key Laboratory for Functional Substances in Medicinal Edible Resources and Healthcare Products, School of Life Sciences and Food Engineering, Hanshan Normal University, Chaozhou, China

**Keywords:** *Ganoderma cochlear*, *Ganoderma lucidum*, meroterpenoid dimers, dimercochlearlactone A−J, triple negative breast cancer

## Abstract

(±)-Dimercochlearlactones A−J (**1**–**10**), ten pairs of novel meroterpenoid dimers and one known spirocochlealactone A (**11**), were isolated from *Ganoderma* mushrooms. The structural elucidation of new compounds, including their absolute configurations, depends on spectroscopic analysis and electronic circular dichroism (ECD) calculations. Biological studies showed that (+)- and (–)-**2**, (–)-**3**, and (+)- and (–)-**11** are cytotoxic toward human triple negative breast cancer (TNBC) cells (MDA-MB-231) with IC_50_ values of 28.18, 25.65, 11.16, 8.18, and 13.02 μM, respectively. Wound healing assay revealed that five pairs of meroterpenoids (±)-**5**−(±)-**8** and (±)-**10** could significantly inhibit cell mobility at 20 μM in MDA-MB-231 cells. The results provide a new insight into the biological role of *Ganoderma* meroterpenoids in TNBC.

## Introduction

Triple negative breast cancer (TNBC), a subgroup of breast cancer, is often found as high grade of invasive ductal carcinoma with aggressive behavior (O'Reilly et al., 2021). The incidence rate of TNBC accounts for approximately 15%–20% of breast cancers (Chen et al., 2021; Chowdhury et al., 2021). “Triple negative” is regarded as the absence of the expression of three receptors, estrogen receptor (ER), progesterone receptor (PR), and human epidermal growth factor 2 receptor (HER2). It causes an about threefold shorter median overall survival (OS) comparing with other breast cancers (O'Reilly et al., 2021). In addition, the relapse and metastasis rate of TNBC is high, the relapse commonly found within 3 years, and the metastasis often occurs in visceral and brain (O'Reilly et al., 2021; Chen et al., 2021; Chowdhury et al., 2021). Due to the difficulties in treating TNBC, more potential molecules are needed.

**FIGURE 1:**
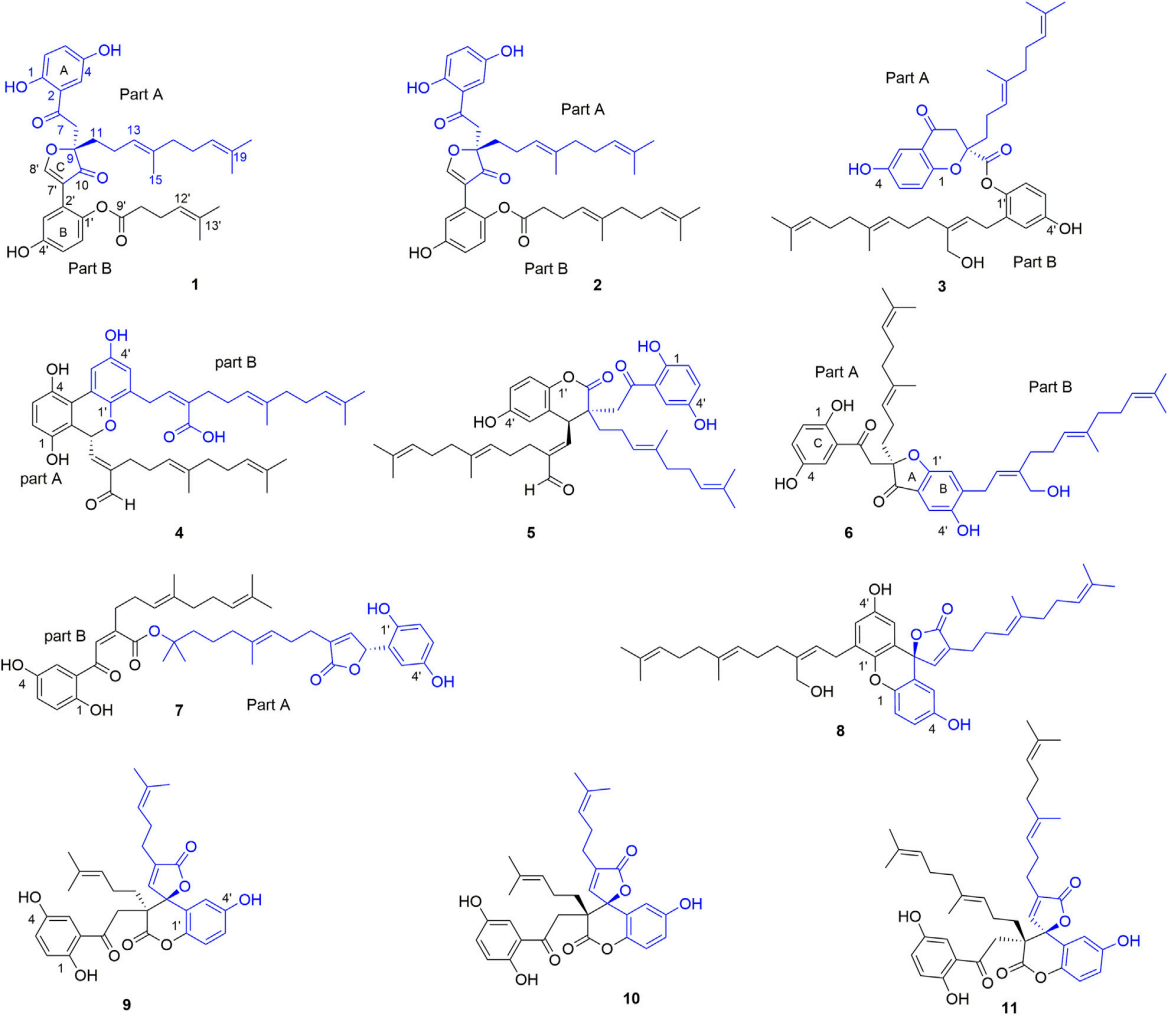
The structures of **1**–**11**.

*Ganoderma* is a traditional Chinese medicine and has been discovered numerous bioactivities as hypoglycemic effect, cardiovascular protection, anti-tumor, antioxidant, and brain injury prevention (Lin and Deng, 2019; Lin and Sun, 2019; Liu and Tie, 2019; Meng and Yang, 2019; Quan et al., 2019). All along, *Ganoderma* triterpenoids have been considered as the main active components with anti-tumor effects. In recent years, *Ganoderma* meroterpenoids, which process phenol moiety and terpene moiety, have been continuously excavated significant anti-tumor activities, such as toward human cancer cell lines (A549, KYSE30, BT549, and MDA-MB-231) (Qin et al., 2018; Cai et al., 2021; Zhang et al., 2021). For the purpose of discovering active agents toward TNBC from natural sources, eleven meroterpenoid dimers including ten novel ones were isolated from *Ganoderma* (Figure 1)*.* This paper deals with their isolation, structural elucidation, and biological evaluation for cytotoxicity and cell migration inhibition in TNBC cells.

## Materials and Methods

### General

An Anton Paar MCP-100 digital polarimeter was used to collect optical rotations data. UV and CD spectra were measured on a Chirascan instrument. NMR spectra were collected by a Bruker Avance Ⅲ 600 MHz or a 500-MHz spectrometer, and internal standard is TMS. HRESIMS were recorded on a Waters Xevo G2-XS QTOF or a Shimazu LC-20AD AB Sciex X500R MS spectrometer (Shimadzu Corporation, Tokyo, Japan). C-18 silica gel (40–60 μm; Daiso Co., Japan), MCI gel CHP 20P (75–150 μm, Mitsubishi Chemical Industries, Tokyo, Japan), Sephadex LH-20 (Amersham Pharmacia, Uppsala, Sweden), and Silica gel (Qingdao Marine Chemical Inc., Qingdao, China) were used for column chromatography. Preparative HPLC was carried out using a Chuangxin-Tongheng chromatograph equipped with a Thermo Hypersil GOLD-C18 column (250 mm × 21.2 mm, i.d., 5 μm). Semi-preparative HPLC was taken on a SEP-LC52 chromatograph with a YMC-Pack ODS-A column (250 mm × 10 mm, i.d., 5 μm). Chiral HPLC analysis was taken on an Agilent 1260 or SEP-LC52 chromatograph with a Daicel Chiralpak column (IC, 250 mm × 10 mm, i.d., 5 μm).

### Fungal Material

*Ganoderma cochlear* were purchased from Guangzhou Tongkang Pharmaceutical Co. Ltd. (Guangdong Province, China) in July 2014. *Ganoderma lucidum* were collected from Dayao County, Yunnan Province, China, in April 2018. Prof. Zhu-Liang Yang from Kunming Institute of Botany, Chinese Academy of Sciences, Kunming, China, authenticated these fungi. The voucher specimens (CHYX-0589 for *G. cochlea*r and CHYX-0615 for *G. lucidum*) are deposited at the School of Pharmaceutical Sciences, Shenzhen University Health Science Center, China.

### Extraction and Isolation

Powdered fruiting bodies of *G. cochlear* (200 kg) were extracted using refluxing 80% EtOH (3 × 120 L, 4, 3, 3 h) to yield a crude extract. An aliquot (8 kg of the residue corresponding to 95 kg fungal material) was suspended in H_2_O and extracted three times with EtOAc. The EtOAc soluble residue (4 kg) was then cut into four parts (Fr.1–Fr.4) by a silica gel column with increasing acetone in petroleum ether (10:1–0:1). Fr.2 (860 g) was separated by an MCI gel CHP 20P column (aqueous MeOH, 50%–100%) to get six subfractions (Fr.2.1–Fr.2.6). Of which, the subfraction of Fr.2.2 (120.0 g) was submitted to an RP-18 column (aqueous MeOH, 40%–100%) to obtain five parts (Fr.2.2.1−Fr.2.2.5). Fr.2.2.2 (12.4 g) was fractionated into three parts (Fr.2.2.2.1–Fr.2.2.2.3) by an MCI gel CHP 20P column (aqueous MeOH, 40%–100%). Among them, the last part (7.4 g) was purified by using Sephadex LH-20 (MeOH) and then separated by semi-preparative HPLC (MeOH/H_2_O, containing 0.05% TFA in H_2_O, 78%, flow rate: 3 ml/min) to get compounds **9** (t_R_ = 17.0 min, 30.2 mg) and **10** (t_R_ = 23.5 min, 36.5 mg).

Fr.2.5 (70 g) was fractionated into four parts (Fr.2.5.1–Fr.2.5.6) by a silica gel column eluted by increasing acetone in petroleum ether (10:1–0:1). Of which, Fr.2.5.4 (12.0 g) was divided into three parts (Fr.2.5.4.1–Fr.2.5.4.3) by an MCI gel CHP 20P column (aqueous MeOH, 70%–100%). The second part (6.0 g) was first gel filtrated over Sephadex LH-20 (MeOH), then cut by preparative HPLC (aqueous MeOH, 65%–100%) to get five parts (Fr.2.5.4.2.1–Fr.2.5.4.2.5). Among them, Fr.2.5.4.2.4 (500.0 mg) was further purified by semi-preparative HPLC (aqueous MeOH containing 0.05% TFA, 93%, flow rate: 3 ml/min) to afford **3** (t_R_ = 15.6 min, 10.0 mg) and **4** (t_R_ = 24.8 min, 25.6 mg).

Fr.2.5.5 (15.0 g) was fractionated into five parts (Fr.2.5.5.1–Fr.2.5.5.5) by a C-18 column (aqueous MeOH, 70%–100%). Of which, Fr.2.5.5.3 (3.0 g) was first filtrated by using Sephadex LH-20 (MeOH), then divided into six parts (Fr.2.5.5.3.1–Fr.2.5.5.3.6) by preparative HPLC (aqueous MeOH, 65%–100%). Fr.2.5.5.3.5 (150.0 mg) was purified *via* semi-preparative HPLC (aqueous MeOH containing 0.05% TFA, 92%, flow rate: 3 ml/min) to afford **1** (t_R_ = 12.5 min, 8.0 mg), **8** (t_R_ = 13.3 min, 8.6 mg), **5** (t_R_ = 15.8 min, 0.8 mg), and the impure part was further purified by semi-preparative HPLC to afford **2** (t_R_ = 25.8 min, 1.8 mg) (acetonitrile/H_2_O, 85% containing 0.05% TFA, flow rate: 3 ml/min). The subfraction of Fr.2.5.5.3.4 (200.0 mg) was purified by semi-preparative HPLC (aqueous MeOH containing 0.05% TFA, 88%, flow rate: 3 ml/min) to afford **6** (t_R_ = 14.8 min, 10.0 mg).

Fr.3 (780.0 g) was divided into eight subfractions (Fr.3.1–Fr.3.8) by an MCI gel CHP 20P column (aqueous MeOH, 40%–100%). Of which, Fr.3.6 (282.0 g) was first purified by Sephadex LH-20 (MeOH) to provide three parts (Fr.3.6.1–Fr.3.6.3). The last part (146.8 g) was frationated into four parts (Fr.3.6.3.1–Fr.3.6.3.4) using a C-18 column (aqueous MeOH, 50%–100%). Of them, Fr.3.6.3.2 (81.0 g) was also first filtrated by Sephadex LH-20 (MeOH), and then cut by an MCI gel CHP 20P column (aqueous MeOH, 40%–100%) to provide five parts (Fr.3.6.3.2.1–Fr.3.6.3.2.5). Among them, Fr.3.6.3.2.4 (23.0 g) filtrated by Sephadex LH-20 (MeOH) followed by a C-18 column (elution solvent: aqueous MeOH, 50%–100%) to get seven parts (Fr.3.6.3.2.4.1-Fr.3.6.3.2.4.7). Fr.3.6.3.2.4.5 (3.9 g) was filtrated by using Sephadex LH-20 (MeOH), then purified by semi-preparative HPLC (aqueous acetonitrile, 78% containing 0.05% TFA, flow rate: 3 ml/min) to obtain **7** (7.5 mg, t_R_ = 20.6 min).

The dried fruiting bodies of *G. lucidum* (30.0 kg) were powdered and extracted with 95% EtOH under percolation (240 L) at room temperature to afford a crude extract (2.1 kg), which was partitioned between water and EtOAc for three times to obtain an EtOAc extract (1.1 kg). The extract was submitted to an MCI gel CHP 20P column (aqueous MeOH, 40%–100%) to obtain 13 fractions (Fr.1–Fr.13). Fr.13 (228.0 g) was cut by a silica gel column eluted by increasing acetone in petroleum ether (10:1−3:1) to give two parts (Fr.13.1 and Fr.13.2). The second part (50.9 g) was submitted to Sephadex LH-20 (MeOH) to obtain three parts (Fr.13.2.1–Fr.13.2.3). Fr.13.2.2 (2.5 g) was separated by *vacuum* liquid chromatography (VLC) with increasing acetone in petroleum ether (10:1–3:1) to give five parts (Fr.13.2.2.1–Fr.13.2.2.5). Among them, Fr.13.2.2.3 (456.0 mg) was purified by preparative HPLC (aqueous AcCN, 57%–95%) to afford **11** (39.6 mg).

Compounds **1**–**11** are racemics, further purification by chiral column (Daicel Chiralpak IC, 250 mm × 10 mm, i.d., 5 μm) (flow rate: 3.0 ml/min) afforded their enantiomers (+)**-1** (3.50 mg, t_R_ = 14.8 min) and (–)**-1** (3.60 mg, t_R_ = 19.9 min) (n-hexane/ethanol, 90:10); (+)**-2** (0.75 mg, t_R_ = 8.6 min) and (–)**-2** (0.79 mg, t_R_ = 10.7 min) (n-hexane/ethanol, 85:15); (+)**-3** (4.70 mg, t_R_ = 20.6 min) and (–)**-3** (4.50 mg, t_R_ = 18.1 min) (n-hexane/ethanol, 92:8); (+)**-4** (10.50 mg, t_R_ = 18.1 min) and (–)**-4** (10.80 mg, t_R_ = 20.8 min) (n-hexane/ethanol, 95:5); (+)**-5** (0.35 mg, t_R_ = 12.3 min) and (–)**-5** (0.38 mg, t_R_ = 13.2 min) (n-hexane/ethanol, 90:10); (+)**-6** (4.70 mg, t_R_ = 24.4 min) and (–)**-6** (4.50 mg, t_R_ = 21.8 min) (n-hexane/ethanol, 90:10); (+)**-7** (3.60 mg, t_R_ = 17.6 min) and (–)**-7** (3.40 mg, t_R_ = 20.9 min) (n-hexane/ethanol, 90:10); (+)**-8** (3.70 mg, t_R_ = 11.9 min) and (–)**-8** (3.50 mg, t_R_ = 13.8 min) (n-hexane/ethanol, 90:10); (+)**-9** (1.32 mg, t_R_ = 19.7 min) and (–)**-9** (1.33 mg, t_R_ = 24.1 min) (n-hexane/ethanol, 95:5), (+)**-10** (0.98 mg, t_R_ = 22.6 min) and (–)**-10** (1.01 mg, t_R_ = 20.3 min) (n-hexane/ethanol, 95:5); (+)**-11** (18.8 mg, t_R_ = 17.2 min) and (–)**-11** (18.5 mg, t_R_ = 15.0 min) (n-hexane/ethanol, 96:4).

### Compound Characterization

Dimercochlearlactone A (**1**): yellowish gum; [α]_D_^20^ +18.9 (*c* 0.09, MeOH); CD (MeOH) *Δε*_370_ + 0.9, *Δε*_310_ –2.5, *Δε*_260_ + 2.8, *Δε*_216_ –3.1; (+)-**1**; [α]_D_^20^ –11.0 (*c* 0.10, MeOH); CD (MeOH) *Δε*_371_ –0.4, *Δε*_310_ + 2.0, *Δε*_260_ –2.2, *Δε*_215_ + 1.5; (–)-**1**; UV (MeOH) *λ*_max_ (log*ε*) 369 (3.42), 284 (58), 254 (3.95), 233 (4.15), 209 (4.31) nm; HRESIMS *m/z* 625.2774 [M + Na]^+^ (calcd for C_36_H_42_NaO_8_, 625.2777). ^1^H and ^13^C NMR data, see Tables 1 and 2.

**TABLE 1:** ^1^H NMR data of **1**–**5** (*δ* in ppm, *J* in Hz).

No	1	2	3	4	5
	δ_H_ ^a^	δ_H_ ^a^	δ_H_ ^b^	δ_H_ ^b^	δ_H_ ^c^
3	7.14 d (3.0)	7.14 d (2.9)	7.16 (d, 3.0)		7.12 d (2.9)
5	6.98 dd (8.9, 3.0)	6.97 dd (8.9, 2.9)	7.06 d (8.9, 3.0)	6.70 d (8.5)	7.03 d (8.7 2.9)
6	6.80 d (8.9)	6.80 d (8.9)	7.01 d (8.9)	6.61 d (8.5)	6.88 d (8.7)
7				6.41 d (9.6)	7.12 d (2.9)
8	Ha: 3.92 d (17.7)	Ha: 3.92 d (17.8)	Ha: 3.20 d (16.9)	6.51 d (9.6)	Ha: 3.90 d (18.3)
	Hb: 3.57 d (17.7)	Hb: 3.57 d (17.8)	Hb: 3.11 d (16.9)		Hb: 2.89 d (18.3)
10				9.17 s	
11	Ha: 1.86 m	Ha: 1.84 m	2.15 m	Ha: 2.63 m	Ha: 1.75 m
	Hb: 1.74 m	Hb: 1.74 m		Hb: 2.50 m	Hb: 1.55 m
12	1.97 m	1.97 m	Ha: 2.36 m	2.19 m	1.91 overlap
	1.83 m	1.85 m	Hb: 2.26 m		
13	5.03 t (6.9)	5.04 overlap	5.20 t (6.9)	5.25 t (6.9)	4.92 t (6.9)
15	1.52 s	1.52 s	1.66 s	1.56 s	1.46 s
16	1.91 m	1.92 m	2.03 m	1.91 overlap	1.82 m
17	2.00 m	2.00 m	2.10 overlap	2.02 overlap	2.02 overlap
18	5.07 t (6.9)	5.04 overlap	5.07 overlap	5.05 overlap	5.04 t (6.9)
20	1.54 s	1.54 s	1.58 s	1.59 s	1.56 s
21	1.63 s	1.62 s	1.65 s	1.66 s	1.66 s
3′	7.10 d (2.9)	7.10, d (2.9)	6.57 d (2.7)	8.00 d (3.0)	6.41 d (2.4)
5′	6.71 dd (8.8, 2.9)	6.71 dd (8.8, 2.9)	6.54 dd (8.6, 2.7)	6.57 d (3.0)	6.77 d (8.7, 2.4)
6′	6.93 d (8.8)	6.93 d (8.8)	6.51 d (8.6)		7.01 d (8.7)
7′			Ha: 2.83 dd (16.0, 7.4)	Ha: 3.83 dd (15.6, 7.3)	5.21 d (10.7)
			Hb: 2.79 dd (16.0, 7.4)	Hb: 3.65 dd (15.6, 7.9)	
8′	8.77 s	8.77 s	5.13 t-like (7.4)	5.96 t-like (7.6)	6.47 d (10.7)
10′	2.51 m	2.50 m	4.08 s		9.57 s
11′	2.26 q (7.4)	2.26 q (7.4)	2.14 m	2.18 m	Ha: 2.26 m
					Hb: 2.00 m
12′	5.09 t-like (7.0)	5.11 t-like (7.0)	2.10 overlap	2.10 m	Hb: 2.06 m
					Hb: 1.91 m
13′			5.12 t (6.7)	5.15 t (6.7)	4.97 t (6.9)
14′	1.55 s	1.56 s			
15′	1.63 s	1.92 m	1.59 s	1.66 s	1.52 s
16′		2.00 m	1.96 m	1.91 m	1.92 m
17′		5.04 overlap	2.04 overlap	2.02 m	2.02 m
18′			5.10 overlap	5.05 overlap	5.00 t (6.9)
19′		1.54 s			
20′		1.62 s	1.60 s	1.58 s	1.56 s
21′			1.67 s	1.64 s	1.66 s
1-OH	10.75 s	10.76 s			
4-OH	9.17 s	9.17 s	9.51^d^ s		11.43
4′-OH	9.50 s	9.51 s	9.41^d^ s		

a Record in 500 MHz in DMSO-*d*_6_.

b Record in 600 MHz in methanol-*d*_4_.

c Record in 600 MHz in CDCl_3_.

d Observed in DMSO-*d*_6_.

**TABLE 2:** ^13^C NMR data of **1**–**10** (*δ* in ppm, *J* in Hz).

No	1^a^	2^a^	3^b^	4^b^	5^c^	6^c^	7^a^	8^c^	9^c^	10^c^
1	153.0 s	153.0 s	153.7 s	147.1 s	156.9 s	156.7 s	154.7 s	144.9 s	156.8 s	156.6 s
2	120.8 s	120.8 s	122.3 s	122.1 s	118.7 s	118.8 s	120.4 s	116.4 s	119.0 s	118.7 s
3	114.8 d	114.8 d	111.4 d	117.9 s	113.9 d	114.9 d	116.0 d	112.0 d	114.4 d	113.9 d
4	149.4 s	149.4 s	155.5 s	149.1 s	147.4 s	147.5 s	150.0 s	151.5 s	148.1 s	147.8 s
5	124.1 d	124.2 d	126.5 d	117.8 d	125.6 d	125.4 d	125.2 d	118.6 d	126.4 d	125.9 d
6	118.3 d	118.2 d	120.8 d	116.3 d	119.8 d	119.4 d	118.8 d	118.4 d	119.7 d	119.8 d
7	199.1 s	199.1 s	192.7 s	69.3 d	202.4 s	200.3 s	197.0 s	81.4 s	203.5 s	201.8 s
8	45.1 t	45.2 t	45.3 t	149.6 d	41.4 t	44.0 t	130.0 d	151.4 s	33.9 t	38.1 t
9	88.2 s	88.2 s	85.8 s	144.9 s	46.0 s	89.0 s	144.4 s	132.4 s	52.2 s	50.9 s
10	202.6 s	202.6 s	172.8 s	197.2 d	169.7 s	203.3 s	166.7 s	173.8 s	168.5 s	167.8 s
11	36.2 t	36.2 t	39.5 t	25.3 t	31.5 t	36.6 t	33.9 t	25.0 t	30.5 t	33.7 t
12	20.7 t	20.7 t	23.2 t	28.6 t	26.9 t	21.6 t	26.5 t	25.9 t	23.7 t	25.6 t
13	122.4 d	122.5 d	124.0 d	124.8 d	122.2 d	122.6 d	123.0 d	122.4 d	122.1 d	122.0 d
14	135.5 s	135.5 s	137.9 s	137.0 s	137.2 s	136.0 s	136.4 s	137.1 s	133.7 s	133.5 s
15	15.6 q	15.6 q	16.3 q	16.2 q	15.9 q	16.1 q	16.3 q	16.3 q	17.8 q	17.6 q
16	39.1 t	39.0 t	41.0 t	40.8 t	39.5 t	39.5 t	39.6 t	39.6 t	25.7 q	25.6 q
17	26.0 t	26.0 t	27.8 t	27.8 t	26.4 t	26.5 t	26.6 t	26.5 t		
18	122.5 d	123.9 d	125.5 d	125.5 d	124.0 d	124.1 d	124.5 d	124.1 d		
19	130.6 s	130.6 s	132.5 s	132.0 s	131.7 s	131.4 s	131.2 s	131.5 s		
20	17.4 q	17.4 q	18.0 q	17.8 q	17.7 q	17.7 q	18.0 q	17.7 q		
21	25.3 q	25.3 q	26.1 q	25.9 q	25.7 q	25.7 q	25.9 q	25.7 q		
1′	154.7 s	154.6 s	142.5 s	144.1 s	152.6 s	149.9 s	147.7 s	142.8 s	152.9 s	153.7 s
2′	122.5 s	122.5 s	135.3 s	124.0 s	125.6 d	139.4 d	122.3 s	116.6 s	121.5 s	121.8 s
3′	114.9 d	114.9 d	117.4 d	114.7 d	114.0 d	113.7 d	112.7 d	118.7 d	112.6 d	113.9 d
4′	139.5 s	139.5 s	157.0 s	152.1 s	144.3 s	166.2 s	150.5 s	151.0 s	144.0 s	152.7 s
5′	114.5 d	114.5 d	114.7 d	117.1 d	115.4 d	120.3 s	116.6 d	109.8 d	118.4 d	118.5 d
6′	123.5 d	123.5 d	123.2 d	124.0 s s	117.7 d	108.8 d	116.8 d	130.7 s	119.7 d	110.2 d
7′	115.0 s	115.1 s	28.5 t	31.7 t	40.1 d	31.2 t	77.9 d	28.0 t	88.3 s	87.1 s
8′	174.8 d	174.8 d	125.4 d	141.2 d	146.0 d	125.9 d	149.9 d	125.6 d	146.3 d	152.7 d
9′	171.2 s	171.2 s	141.4 s	132.9 s	149.2 s	139.6 s	131.8 s	139.9 s	136.7 s	129.2 s
10′	33.7 t	33.7 t	60.1 t	171.5 s	194.0 s	61.1 t	174.2 s	60.4 t	172.1 s	173.6 s
11′	22.8 t	22.8 t	35.9 t	36.1 t	24.6 t	36.6 t	25.3 t	35.3 t	25.1 t	24.7 t
12′	123.9 d	122.2 d	28.5 t	28.6 t	23.0 t	26.8 t	25.9 t	26.7 t	25.2 t	23.2 t
13′	132.2 s	135.8 s	125.4 d	124.4 d	121.9 d	123.4 d	123.4 d	123.7 d	122.2 d	122.0 d
14′	17.4 q	15.6 q	136.4 s	136.9 s	137.0 s	136.5 s	136.0 s	135.7 s	133.8 s	133.6 s
15′	25.4 q	39.0 t	16.4 q	16.2 q	16.1 q	15.9 q	16.0 q	16.1 q	17.8 q	17.7 q
16′		26.0 t	41.0 t	40.9 t	39.4 t	39.6 t	39.5 t	39.6 t	25.6 q	25.5 q
17′		123.9 d	27.9 t	27.8 t	26.5 t	26.6 t	21.7 t	26.5 t		
18′		130.6 s	125.7 d	125.5 d	124.2 d	124.1 d	40.3 t	124.1 d		
19′		17.4 q	132.2 s	132.1 s	131.6 s	131.5 s	84.5 s	131.7 s		
20′		25.4 q	18.0 q	17.8 q	17.7 q	17.7 q	25.5 q	17.7 q		
21′			26.1 q	25.9 q	25.7 q	25.7 q	25.5 q	25.7 q		

a Record in 125 MHz in DMSO-*d*_6_.

b Record in 150 MHz in methanol-*d*_4_.

c Record in 150 MHz in CDCl_3_.

Dimercochlearlactone B (**2**): yellowish gum; [α]_D_^20^ +17.0 (*c* 0.11, MeOH); CD (MeOH) *Δε*_372_ + 1.0, *Δε*_311_ –3.3, *Δε*_260_ + 3.5, *Δε*_209_ –6.3; (+)-**2**; [α]_D_^20^ –14.7 (*c* 0.11, MeOH); CD (MeOH) *Δε*_369_ –0.4, *Δε*_309_ + 1.7, *Δε*_260_ –2.0, *Δε*_214_ + 2.8; (–)-**2**; UV (MeOH) *λ*_max_ (log*ε*) 369 (3.31), 285 (3.49), 259 (3.87), 233 (4.10), 201 (4.39) nm; HRESIMS *m/z* 693.3406 [M + Na]^+^ (calcd for C_41_H_50_NaO_8_, 693.3403). ^1^H and ^13^C NMR data, see Tables 1 and 2.

Dimercochlearlactone C (**3**): yellowish gum; [α]_D_^20^ +30.6 (*c* 0.12, MeOH); CD (MeOH) *Δε*_329_ + 3.5 *Δε*_229_ –11.6, ; (+)-**3**; [α]_D_^20^ –57.6 (*c* 0.12, MeOH); CD (MeOH) *Δε*_330_ – 4.2, *Δε*_231_ + 7.8; (–)-**3**; UV (MeOH) *λ*_max_ (log*ε*) 356 (3.55), 283 (3.50), 254 (3.96), 221 (4.48), 203 (4.81) nm; HRESIMS *m/z* 669.3777 [M – H]^–^ (calcd for C_42_H_53_O_7_, 669.3797). ^1^H and ^13^C NMR data, see Tables 1 and 2.

Dimercochlearlactone D (**4**): yellowish gum; [α]_D_^20^ +4.5 (*c* 0.17, MeOH); CD (MeOH) *Δε*_350_ + 4.6, *Δε*_279_ –9.2, *Δε*_220_ + 13.6, *Δε*_205_ –8.5; (+)-**4**; [α]_D_^20^ –4.7 (*c* 0.22, MeOH); CD (MeOH) *Δε*_352_ –5.5, *Δε*_280_ + 9.1, *Δε*_220_ –14.4, *Δε*_204_ + 8.8; (–)-**4**; UV (MeOH) *λ*_max_ (log*ε*) 350 (3.62), 225 (4.44) nm; HRESIMS *m/z* 667.3620 [M – H]^–^ (calcd for C_42_H_51_O_7_, 667.3640). ^1^H and ^13^C NMR data, see Tables 1 and 2.

Dimercochlearlactone E (**5**): yellowish gum; [α]_D_^20^ +26.9 (*c* 0.30, MeOH); CD (MeOH) *ε*_368_ + 0.9, *Δε*_279_ + 1.5, *Δε*_265_ –1.0, *Δε*_250_ + 1.7, *Δε*_232_ –7.6, *Δε*_216_ + 2.9; *Δε*_205_ –3.0; (+)-**5**; [α]_D_^20^ –19.0 (*c* 0.32, MeOH); CD (MeOH) *ε*_368_ –0.9, *Δε*_280_ –1.5, *Δε*_264_ + 1.4, *Δε*_249_ –1.4, *Δε*_233_ + 7.9, *Δε*_216_ –4.0, *Δε*_204_ + 3.7 (–)-**5**; UV (MeOH) *λ*_max_ (log*ε*) 369 (3.36), 285 (3.34), 255 (3.87), 222 (4.28), 203 (4.50) nm; HRESIMS *m/z* 781.3580 [M + CF_3_COO]^–^ (calcd for C_44_H_52_F_3_O_9_, 781.3569). ^1^H and ^13^C NMR data, see Tables 1 and 2.

Dimercochlearlactone F (**6**): yellowish gum; [α]_D_^20^ +38.2 (*c* 0.10, MeOH); CD (MeOH) *ε*_381_ –5.7, *Δε*_319_ + 5.5, *Δε*_255_ + 3.6, *Δε*_215_ –3.5; (+)-**6**; [α]_D_^20^ –16.4 (*c* 0.11, MeOH); CD (MeOH) *ε*_380_ + 4.7, *Δε*_320_ –4.0, *Δε*_256_ –3.8 *Δε*_214_ + 2.0; (–)-**6**; UV (MeOH) *λ*_max_ (log*ε*) 368 (3.94), 262 (4.24), 229 (4.40), 203 (4.64) nm; HRESIMS *m/z* 783.3727 [M + CF_3_COO]^–^ (calcd for C_44_H_54_F_3_O_9_, 783.3725). ^1^H and ^13^C NMR data, see Tables 2 and 3.

**TABLE 3:** ^1^H NMR data of **6**–**10** (*δ* in ppm, *J* in Hz).

No	6	7	8	9	10
	δ_H_ ^a^	δ_H_ ^b^	δ_H_ ^a^	δ_H_ ^a^	δ_H_ ^a^
3	7.11 d (2.7)	7.07 d (3.0)	6.57 d (2.9)	7.14 d (2.9)	7.12 d (8.9)
5	6.99 d (8.9, 2.7)	7.01 d (8.9, 3.0)	6.88 d (8.9, 2.9)	7.08 dd (8.9, 2.9)	7.04 dd (8.9, 2.9)
6	6.80 d (8.9)	6.82 d (8.9)	7.12 d (8.9)	6.84 d (8.9)	6.84 d (8.9)
8	Ha: 3.62 d (13.8)	6.92 s	6.98 s	Ha: 3.47 d (17.4)	Ha: 3.68 d (18.4)
	Hb: 3.45 d (13.8)			Hb: 3.37 d (17.4)	Hb: 3.07 d (18.4)
11	Ha: 1.95 m	2.37 t (7.4)	2.50 m	Ha: 2.21 m	1.82 m
	Hb: 1.91 m			Hb: 1.63 m	
12	1.90 m	2.19 m	2.37 m	2.10 m	1.94 m
13	5.01 t (6.9)	5.14 t (6.9)	5.15 t (6.9)	4.96 overlap	4.85 overlap
15	1.57 s	1.59 s	1.63 s	1.53 s	1.42 s
16	1.95 m	1.97 m	2.00 m	1.60 s	1.54 s
17	2.03 m	2.03 m	2.04 m		
18	5.04 overlap	5.07 t (6.9)	5.04 overlap		
20	1.58 s	1.54 s	1.60 s		
21	1.66 s	1.61 s	1.63 s		
3′	6.85 s	6.35 d (2.9)	6.44 d (2.9)	6.45 d (2.9)	6.57 d (2.9)
5′		6.57 dd (8.6, 2.9)	6.77 d (2.8)	6.85 dd (8.9, 2.9)	6.87 dd (8.9, 2.9)
6′	7.08 s	6.69 d (8.6)		6.98 d (8.9)	7.04 d (8.9)
7′	3.51 m	6.16 d (1.5)	3.57 d		
8′	5.41 t (7.8)	7.39 d (1.5)	5.49 t (6.9)	7.06 s	7.16 s
10′	4.29 m		4.31 s		
11′	2.15 m	2.23 m	2.21 m	Ha: 2.06 m	Ha: 2.06 m
				Hb: 1.86 m	Hb: 1.98 m
12′	2.13 m	2.20 m	2.17 m	2.10 m	Ha: 2.02 m
					Hb: 1.85 m
13′	5.09 t (6.9)	5.04 t (6.7)	5.12 t (6.9)	4.96 overlap	4.85 overlap
15′	1.47 s	1.47 s	1.60 s	1.58 s	1.48 s
16′	1.90 m	1.80 m	1.95 m	1.67 s	1.61 s
17′	2.03 m	1.21 m	2.04 m		
18′	5.04 overlap	1.48 m	5.04 overlap		
20′	1.58 s	1.26 s	1.57 s		
21′	1.66 s	1.27 s	1.66 s		
1-OH		11.20 s			
4-OH	11.33 s	9.18 s	9.42*^c^* s	11.73	11.37 s
1′-OH		9.21 s			
4′-OH		8.77 s	9.29*^c^* s		

a Record in 600 MHz in CDCl_3_.

b Record in 500 MHz in DMSO-*d*_6._

c Observed in DMSO-*d*_6_.

Dimercochlearlactone G (**7**): yellowish gum; [α]_D_^20^ +18.6 (*c* 0.09, MeOH); CD (MeOH) *Δε*_209_ + 12.1; (+)-**7**; [α]_D_^20^ –20.2 (*c* 0.10, MeOH); CD (MeOH) *Δε*_207_ –14.2; (–)-**7**; UV (MeOH) *λ*_max_ (log*ε*) 380 (3.42) 259 (3.95) nm; HRESIMS *m/z* 723.3501 [M + Na]^+^ (calcd for C_42_H_52_NaO_9_, 723.3509). ^1^H and ^13^C NMR data, see Tables 2 and 3.

Dimercochlearlactone H (**8**): yellowish gum; [α]_D_^20^ +17.6 (*c* 0.09, MeOH); CD (MeOH) *Δε*_230_ + 1.7; *Δε*_210_ –8.4; (+)-**8**; [α]_D_^20^ –18.9 (*c* 0.10, MeOH); CD (MeOH) *Δε*_228_ –2.0; *Δε*_207_ + 6.4; (–)-**8**; UV (MeOH) *λ*_max_ (log*ε*) 316 (3.85), 242 (4.33), 203 (4.79) nm; HRESIMS *m/z* 765.3633 [M + CF_3_COO]^–^ (calcd for C_44_H_52_F_3_O_8_, 765.3620). ^1^H and ^13^C NMR data, see Tables 2 and 3.

Dimercochlearlactone I (**9**): yellowish gum; [α]_D_^20^ +10.1 (*c* 0.08, MeOH); CD (MeOH) *Δε*_244_ –17.7, *Δε*_213_ –9.4; (+)-**9**; [α]_D_^20^ −5.1 (*c* 0.07, MeOH); CD (MeOH) *Δε*_239_ + 13.9, *Δε*_209_ + 13.76; (–)-**9**; UV (MeOH) *λ*_max_ (log*ε*) 373 (3.39), 296 (3.35), 254 (3.82), 209 (4.42) nm; HRESIMS *m/z* 569.2154 [M + Na]^+^ (calcd for C_32_H_34_NaO_8_, 569.2152). ^1^H and ^13^C NMR data, see Tables 2 and 3.

Dimercochlearlactone J (**10**): yellowish gum; [α]_D_^20^ +58.5 (*c* 0.09, MeOH); CD (MeOH) *Δε*_260_ −4.4, *Δε*_226_ + 31.5; (+)-**10**; [α]_D_^20^ –47.1 (*c* 0.10, MeOH); CD (MeOH) *Δε*_258_ + 7.5, *Δε*_226_ −31.8; (–)-**10**; UV (MeOH) *λ*_max_ (log*ε*) 372 (3.66), 297 (3.57), 254 (4.08), 203 (4.45) nm; HRESIMS *m/z* 569.2149 [M + Na]^+^ (calcd for C_32_H_34_NaO_8_, 569.2152). ^1^H and ^13^C NMR data, see Tables 2 and 3.

### Biological Activity Assay on TNBC Cell Lines (MDA-MB-231)

TNBC cell line MDA-MB-231 was purchased from Procell (Procell Life Science & Technology Co. Ltd., Wuhan, China). Cell culture, cell viability, and wound healing assays were conducted following the reported protocols (Cai et al., 2021).

## Results and Discussion

**FIGURE 2:**
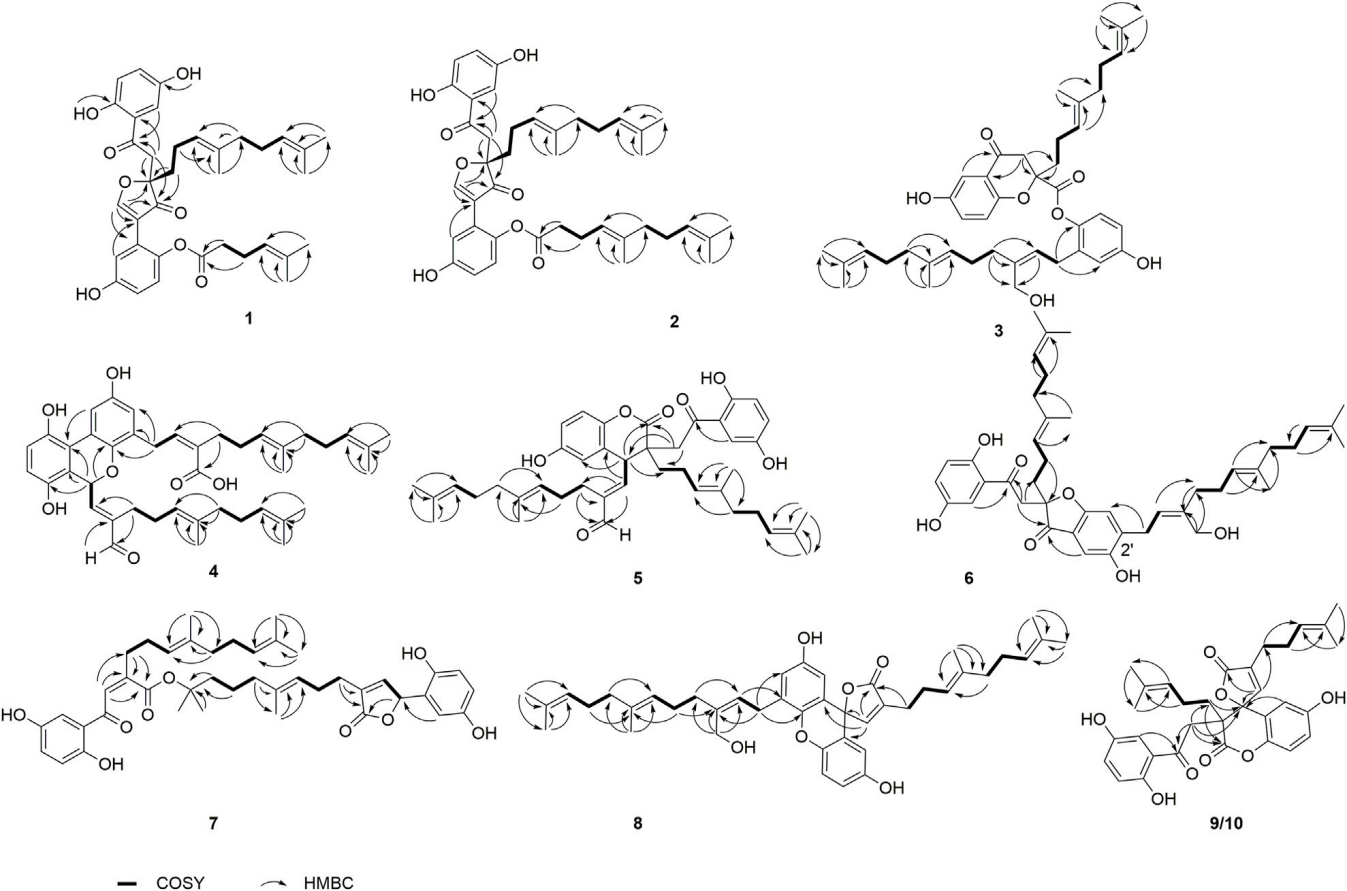
Key COSY and HMBC correlations of **1**–**10**.

Dimercochlearlactone A (**1**) was determined to have a molecular formula as C_36_H_42_O_8_ from its positive HRESIMS (*m/z* 625.2774 [M + Na]^+^, calcd for C_36_H_42_NaO_8_, 625.2777). Two typical ABX spin systems (*δ*_H_ 7.14, d, *J* = 3.0 Hz, H-3; *δ*_H_ 6.98, dd, *J* = 8.9, 3.0 Hz, H-5; *δ*_H_ 6.80, d, *J* = 8.9 Hz, H-6; *δ*_H_ 7.10, d, *J* = 2.9 Hz, H-3′; *δ*_H_ 6.71, dd, *J* = 8.8, 2.9 Hz, H-5′; *δ*_H_ 6.93, d, *J* = 8.8 Hz, H-6′) were observed by its ^1^H NMR data (Table 1). The ^13^C NMR (Table 2) and DEPT spectra show 36 carbon signals, which were assigned as 5 methyl, 7 methylene, 10 methine, and 14 nonprotonated carbons (10 aromatic including 4 oxygenated, 1 oxygenated aliphatic, 2 ketones, and 1 carbonyl). When consideration of the NMR data of the previous reported meroterpenoids (Qin et al., 2018), the above signals suggest that dimercochlearlactone A (**1**) might be a dimeric meroterpenoid. The structure of dimercochlearlactone A (**1**) contains two parts (Parts A and B in Figure 1), which were mainly determined by 2D NMR spectra. For the structure of part A, the ^1^H-^1^H COSY correlations from H_2_-12 (*δ*_H_ 1.97 and 1.83) to H_2_-11 (*δ*_H_ 1.86 and 1.74) and H-13 (*δ*_H_ 5.03), and from H_2_-17 (*δ*_H_ 2.00) to H_2_-16 (*δ*_H_ 1.91) and H-18 (*δ*_H_ 5.07), along with the HMBC correlations (Figure 2) from H_3_-20 (*δ*_H_ 1.54) and H_3_-21 (*δ*_H_ 1.63) to C-18 (*δ*_C_ 122.5) and C-19 (*δ*_C_ 130.6), from H_3_-20 to C-21 (*δ*_C_ 25.3), from H_3_-15 (*δ*_H_ 1.52) and H_2_-16 to C-13 (*δ*_C_ 122.4) and C-14 (*δ*_C_ 135.5), from H_3_-15 to C-16 (*δ*_C_ 39.1), and from H_2_-12 to C-14 suggest the presence of two isoprenyl moieties in dimercochlearlactone A (**1**). In addition, the HMBC correlations from H-8 (*δ*_H_ 3.92 and 3.57) to C-7 (*δ*_C_ 199.1), C-9 (*δ*_C_ 88.2) and C-10 (*δ*_C_ 202.6) and from H_2_-11 to C-9 and C-10 imply another isoprenyl residue. Furthermore, HMBC correlation between H-3 (*δ*_H_ 7.14) and C-7 indicates that the sesquiterpeniod moiety is attached to the ring A *via* C-7 to C-2. Thus, the structure of part A was determined as shown.

**FIGURE 3:**
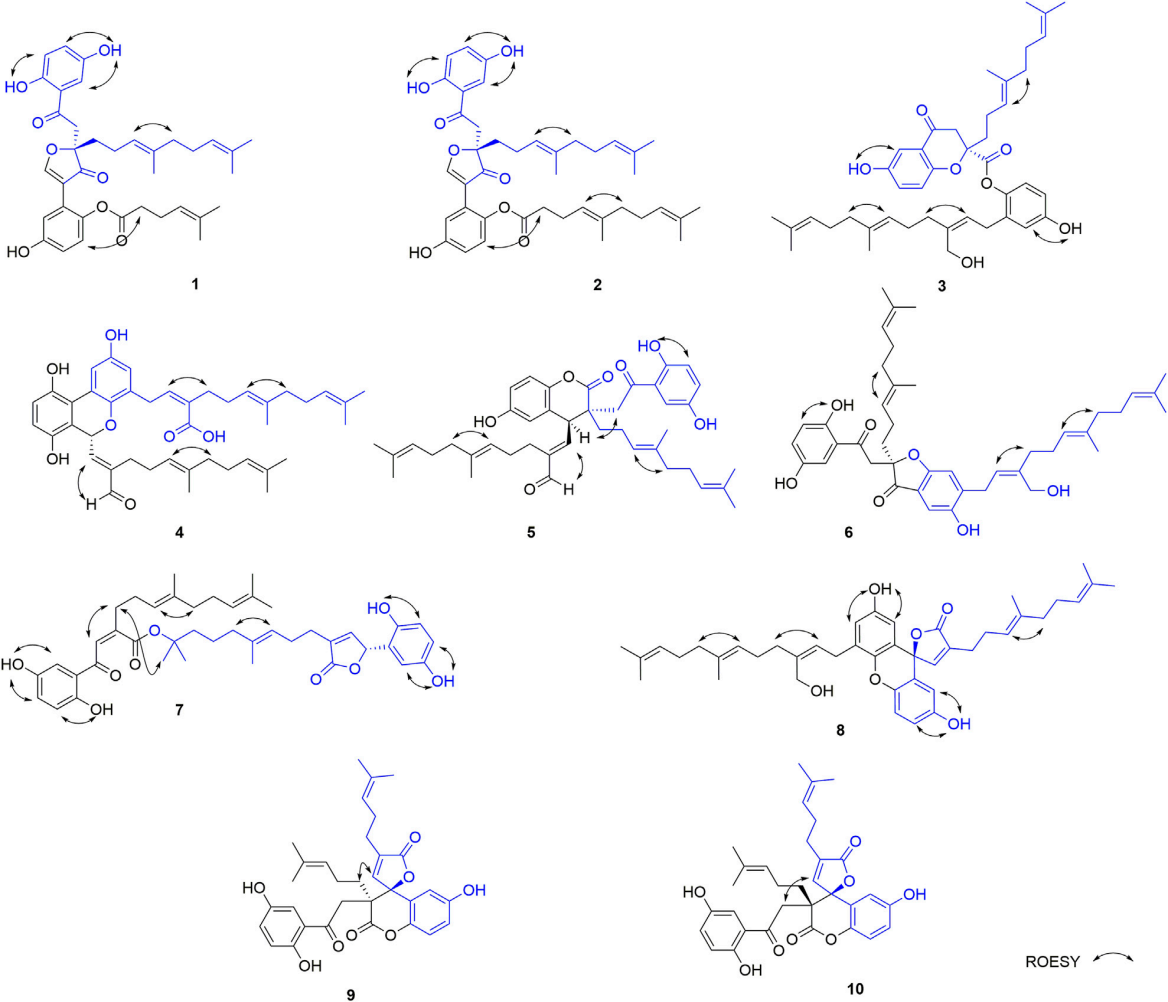
Key ROESY correlations of **1**–**10**.

Part B was also elucidated by 2D NMR experiments (^1^H-^1^H COSY, HSQC and HMBC). The HMBC correlations from H-3′ (*δ*_H_ 7.10) to C-7′, from H-8′ (*δ*_H_ 8.77) to C-2′ (*δ*_C_ 122.5), C-7′ (*δ*_C_ 115.0), C-9 and C-10 and the above-mentioned HMBC correlations from H-8 to C-9 and C-10 not only imply the presence of ring B but also indicate that C-2′ is attached to the ring C. The structure of side chain in part B was confirmed by ^1^H-^1^H COSY correlations observed from H_2_-11′(*δ*_H_ 2.26) to H_2_-10′ (*δ*_H_ 2.51) and H-12′(*δ*_H_ 5.09) and HMBC correlations from H_3_-14′ (*δ*_H_ 1.55) and H_3_-15′ (*δ*_H_ 1.63) to C-12′ (*δ*_C_ 123.9) and C-13′ (*δ*_C_ 132.2), and from H_2_-11′ and H_2_-10′ to C-9′ (*δ*_C_ 171.2). Since the carboxyl group (C-9′) in the side chain of part B needs form an ester with the phenolic hydroxyl group to meet the molecular formula requirement, the position of the side chain in part B linkage can be determined by the following evidence. In the 2D NMR experiments, the HMBC correlations of 1-OH (*δ*_H_ 10.75)/C-1 (*δ*_C_ 153.0), 4-OH (*δ*_H_ 9.17)/C-4 (*δ*_C_ 149.4), and 4′-OH (*δ*_H_ 9.50)/C-4′ (*δ*_C_ 139.5) and ROESY correlations between 1-OH with H-6, 4-OH with H-3, and 4′-OH with H-6 led to the determination of the side chain linkage at the C-1′ position. This conclusion was further secured by the ROESY correlation (Figure 3) between H_2_-10′ and H-6′. Thus, the 2D structure of **1** was assigned.

**FIGURE 4:**
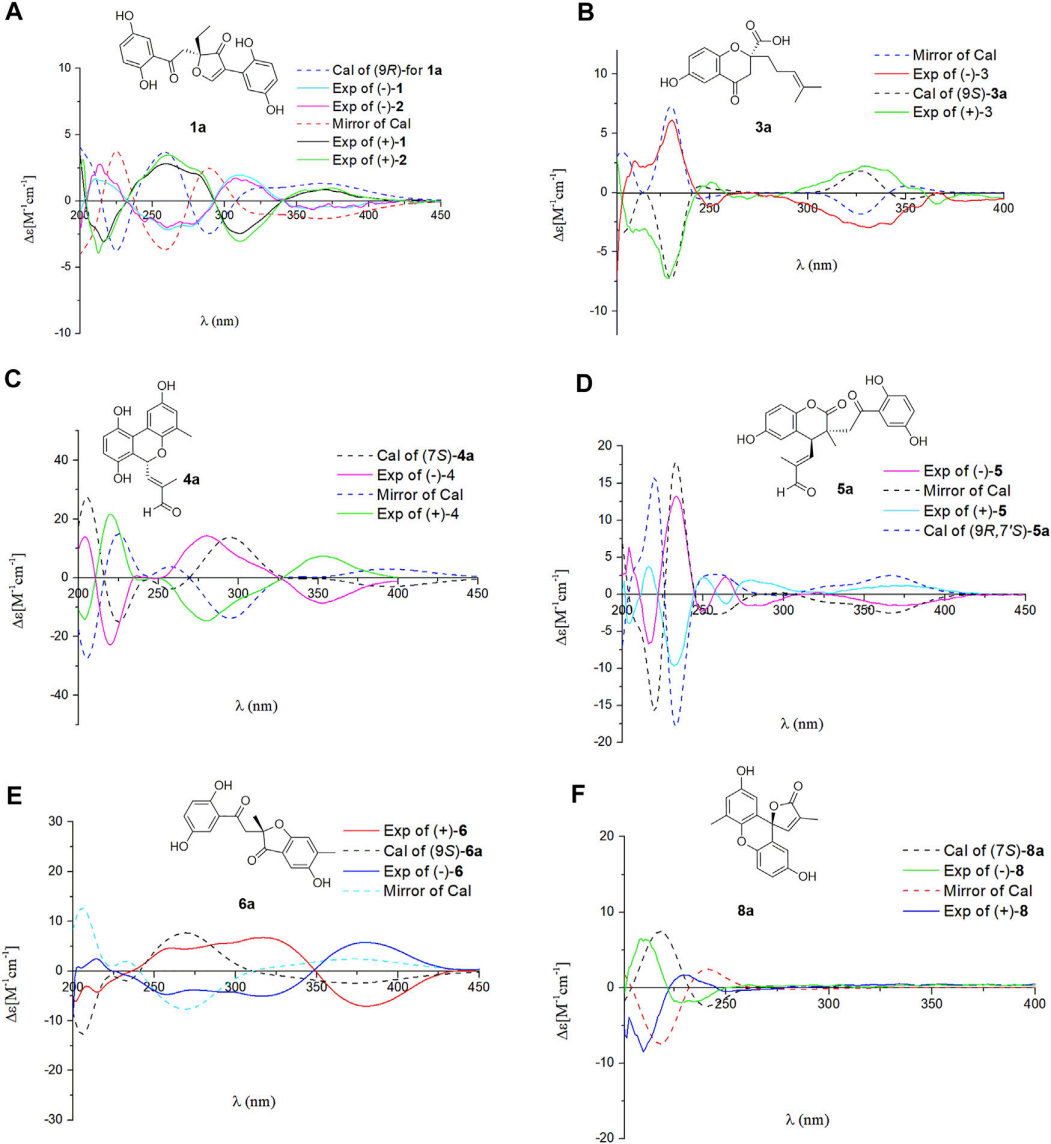
Comparison of the calculated ECD spectra with the experimental CD spectra in MeOH. **(A)** The calculated ECD spectrum of (10*S*)-**1a** at APFD/6-311+G (2d,p) level, σ = 0.30 eV; shift = –12 nm. **(B)** The calculated ECD spectrum of (9*S*)-**3a** at B3LYP/6-31G (d,p) level, σ = 0.20 eV; shift = +20 nm. **(C)** The calculated ECD spectrum of (7*S*)-**4a** at B3LYP/6-31G (d,p) level, σ = 0.30 eV; shift = +0 nm. **(D)** The calculated ECD spectrum of (9*R*,7′*S*)-**5a** at B3LYP/6-31G (d,p) level, σ = 0.20 eV; shift = +0 nm. **(E)** The calculated ECD spectrum of (9*S*)-**6a** at APFD/6-311+G (2d,p) level, σ = 0.40 eV; shift = –10 nm. **(F)** The calculated ECD spectrum of (7*S*)-**8a** at B3LYP/6-31G (d,p) level, σ = 0.30 eV; shift = –30 nm.

In the ROESY spectrum, the correlation between H-13 and H_2_-16 demonstrates that the *Δ* ^13(14)^ double bond is *E* configuration. Dimercochlearlactone A (**1**) was found to be a racemate, the separation by chiral-phase HPLC afforded enantiomers (+)-dimercochlearlactone A (**1**) and (–)-dimercochlearlactone A (**1**). Computational ECD spectral methods at time-dependent density functional theory (TDDFT) were employed to define the absolute configurations of (+)-dimercochlearlactone A (**1**) and (–)-dimercochlearlactone A (**1**). Due to the structure flexibility of dimercochlearlactone A (**1**), the model compound (**1a**) was constructed to ECD calculations. The result showed that the experimental CD spectrum of (+)-dimercochlearlactone A (**1**) exhibited similar Cotton effects with calculated ECD spectrum (Figure 4) of (9*R*)-**1a**. Accordingly, the absolute configurations as 9*R* for (+)-dimercochlearlactone A (**1**) and 9*S* for (–)-dimercochlearlactone A (**1**) were determined.

The NMR data of dimercochlearlactone B (**2**) resemble those of dimercochlearlactone A (**1**) revealing that the structure of dimercochlearlactone B (**2**) similar to that of dimercochlearlactone A (**1**). Only difference appears at the their side chains, which is a 7-carbon side chain in dimercochlearlactone A (**1**) was attached an isopentenyl to form a 12-carbon side chain in dimercochlearlactone B (**2**), supporting by the ^1^H-^1^H COSY correlations from H_2_-16′ (*δ*_H_ 2.00) to H_2_-15′ (*δ*_H_ 1.92) and H-17′ (*δ*_H_ 5.04) and the HMBC correlations (Figure 2) from H_3_-19′ (*δ*_H_ 1.54) and H_3_-20′ (*δ*_H_ 1.62) to C-17′ (*δ*_C_ 123.9) and C-18′ (*δ*_C_ 130.0), from H_2_-14′ (*δ*_H_ 1.56) to C-12′ (*δ*_C_ 122.2), C-13′ (*δ*_C_ 135.8) and C-15′ (*δ*_C_ 39.0). In the ROESY experiment, correlations between H-13 and H_2_-16 and between H-12′ and H_2_-15′ suggest that both double bonds *Δ* ^13(14)^ and *Δ* ^12′(13′)^ are *E*-from configurations (Figure 3). Racemic dimercochlearlactone B (**2**) was separated by chiral HPLC to yield (+)-dimercochlearlactone B (**2**) and (–)-dimercochlearlactone B (**2**). Their absolute configurations were deduced as 9*R* for (+)-dimercochlearlactone B (**2**) and 9*S* for (–)-dimercochlearlactone B (**2**) by using the above-mentioned ECD calculations (Figure 4).

**FIGURE 5:**
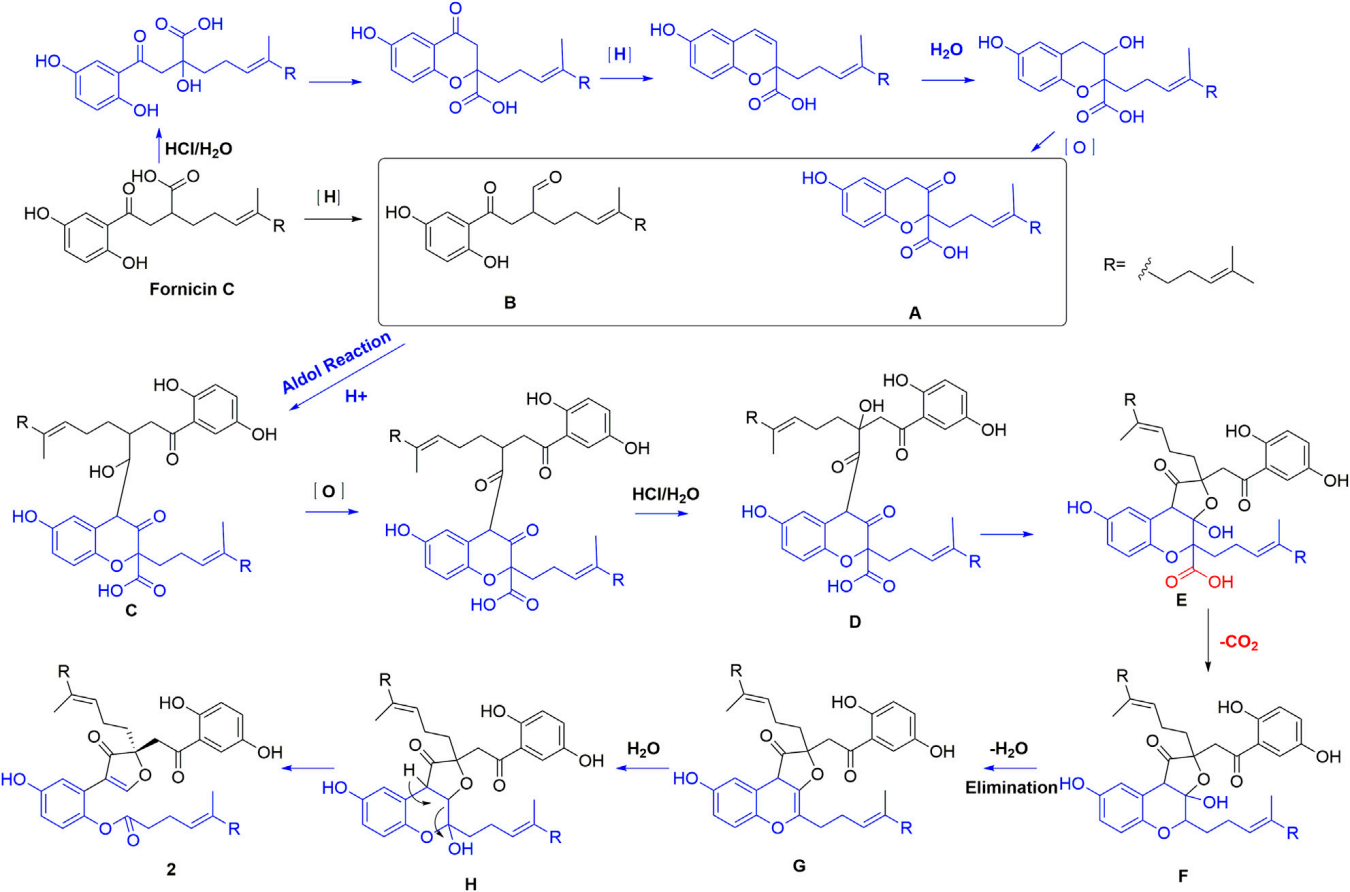
Plausible pathway for the biogenesis of **2**.

Compounds **1** and **2** bear a same skeleton, which are different from the previously isolated *Ganoderma* meroterpenoids, a plausible pathway for the biogenesis of **2** was proposed (Figure 5). At first, fornicin C (Niu et al., 2006) undergoes a series of oxidation, ring formation, and reduction reactions to form intermediates A and B, respectively, which further form intermediate C *via* aldol condensation reaction. Intermediate C undergoes a reduction and substitution reaction to form D, which then give hemiacetal E *via* a substitution addition reaction. After a decarboxylation reaction, hemiacetal E can produce intermediate F. Finally, F undergoes intermediates G and H through dehydration and C-C bond cracking to form **2**.

Dimercochlearlactone C (**3**) has the molecular formula C_42_H_54_O_7_ (16 degrees of unsaturation) deduced by the HRESIMS analysis at *m/z* 669.3777 [M-H]^－^(calcd for C_42_H_53_O_7_, 669.3797). The ^1^H NMR spectrum of dimercochlearlactone C (**3**) exhibits signals for two typical ABX systems (*δ*_H_ 7.16, d, *J* = 3.0 Hz, H-3; *δ*_H_ 7.06, dd, *J* = 8.9, 3.0 Hz, H-5; *δ*_H_ 7.01, d, *J* = 8.9 Hz, H-6; *δ*_H_ 6.57, d, *J* = 2.7 Hz, H-3′; *δ*_H_ 6.54, dd, *J* = 8.6, 2.7 Hz, H-5′; *δ*_H_ 6.51, d, *J* = 8.6 Hz, H-6′). It was found 42 carbon signals including 6 methyl, 11 methylene, 11 methine, and 14 nonprotonated carbons (11 aromatic including 4 oxygenated, 1 oxygenated aliphatic, 1 ketone, and 1 carbonyl) by analyzing its ^13^C NMR and DEPT spectra. Like compound dimercochlearlactone A (**1**), the NMR data of dimercochlearlactone C (**3**) suggest a meroterpenoid dimer. The data of part A are very similar to those of ganotheaecolumol A (Luo et al., 2018), differing in that C-20 is a hydroxymethylene in ganotheaecolumol A, while the same position in part A of dimercochlearlactone C (**3**) is a methyl group. This deduction is supported by the HMBC correlations between H_3_-20 (*δ*_H_ 1.58) with C-18 (*δ*_C_ 125.5), C-19 (*δ*_C_ 132.5), and C-21 (*δ*_C_ 26.1).

The analysis of 2D NMR spectra (See Supplementary Figures S30–S33) of dimercochlearlactone C (**3**) reveals that the structure of part B is similar to that of ganomycin F (Cheng et al., 2018). Thus, there are four possibilities for the connection between part A and part B of dimercochlearlactone C (**3**), C-4-O-C-1′, C-4-O-C-4′, C-9-O-C-1′, and C-9-O-C-4′. In the ^1^H NMR spectrum, signals of two phenolic hydroxyl groups (*δ*_H_ 9.51, s and *δ*_H_ 9.41, s) are observed, it could be concluded that the connections between part A and part B in dimercochlearlactone C (**3**) are C-9-O-C-1′ or C-9-O-C-4′. Furthermore, the ROESY correlations (observed in DMSO-*d*_6_) between 4-OH with H-3 and 4′-O with/H-3′ are indicative of phenolic hydroxyl attaching to C-4 and C-4′, which indicate that C-9 and C-1′ are connected *via* oxygen atom to form phenolic ester. The ROESY correlation between H-8′ (*δ*_H_ 5.13) and H_2_-11′ (*δ*_H_ 2.14) suggests that the double bond *Δ* ^8′(9′)^ is *Z*-form configuration. Furthermore, ROESY correlations between H-13 with H_2_-16 and H-13′ with H_2_-16′ demonstrate that both *Δ* ^13(14)^ and *Δ* ^13′(14′)^ double bonds are *E* configuration. Racemic dimercochlearlactone C (**3**) was segregated into (+)-dimercochlearlactone C (**3**) and (–)-dimercochlearlactone C (**3**) by using chiral HPLC. Since the calculated ECD curve of (9*S*)-**3a** (Model structure) agrees with the experimental CD spectrum of (+)-dimercochlearlactone C (**3**) (Figure 4), the absolute configurations at the stereogenic center were established as 9*S* for (+)-dimercochlearlactone C (**3**) and 9*R* for (–)-dimercochlearlactone C (**3**)**.**

The molecular formula of dimercochlearlactone D (**4**) was deduced as C_42_H_52_O_7_ by its negative HRESIMS. In ^1^H NMR spectrum of dimercochlearlactone D (**4**)**,** the signals at (*δ*_H_ 6.70, d, *J* = 8.5 Hz, H-6; 6.61, d, *J* = 8.5 Hz, H-5; δ_H_ 8.00, d, *J* = 3.0 Hz, H-3′; δ_H_ 6.57, d, *J* = 3.0 Hz, H-5′) suggest that a 1,2,3,4-tetrasubstituted benzene ring and a 1,3,4,5-tetrasubstituted benzene ring in the structure of dimercochlearlactone D (**4**). The ^13^C NMR and DEPT spectra of dimercochlearlactone D (**4**) show 42 carbons including 6 methyl, 9 methylene, 12 methine, and 15 nonprotonated carbons (14 aromatic including 4 oxygenated and 1 carbonyl). The structure of dimercochlearlactone D (**4**) was mainly determined by 2D NMR spectra. The observation correlations from H_2_-12 to H_2_-11 and H-13, and from H_2_-17 to H_2_-16 and H-18 in ^1^H-^1^H COSY spectrum, along with the HMBC correlations from H_3_-20 and H_3_-21 to C-18 and C-19, from H_3_-20 to C-21, from H_3_-15 and H-16 to C-13 and C-14, and from H_3_-15 to C-16 indicate the presence of two isoprenyl moieties. Another isoprenyl residue is supported by the observation of ^1^H-^1^H COSY correlation between H-7 with H-8 and the HMBC correlations from H-10 and H-11 to C-8 and C-9, from H-10 to C-11, and from H-7 to C-9. Further observation of HMBC correlations from H-7 and H-8 to C-2 suggests that C-7 is connected to C-2. Similarly, the ^1^H-^1^H COSY correlations from H_2_-7′ to H-8′, from H_2_-12′ to H_2_-11′ and H-13′, and from H_2_-17′ to H_2_-16′ and H-18′ along with the HMBC correlations from H_3_-20′, and H_3_-21′ to C-18′ and C-19′, from H-20′ to C-21′, from H_3_-15′ and H-16′ to C-13′ and C-14′, from H_3_-15′ to C-16′, from H-10′ and H-11′ to C-8′ and C-9′, and from H-10′ to C-11′ indicates substructure consisting of three isoprenyl groups in part B of **4**. Moreover, the correlation from H-6′ to C-7′ in HMBC spectrum suggests that the side chain and benzene ring of part B are linked *via* C-7′-C-6′. Finally, the two meroterpenoids are linked *via* C-2′-C-3 and C-8-O-C-1′ supported by the key HMBC correlations from H-3′ to C-2 and from H-8 to C-1′. The ROESY correlations from H-8 to H-10, from H-13 to H-16, and from H-13′ to H-16′ indicate that three double bonds (*Δ* ^8(9)^, *Δ* ^13(14)^, and *Δ* ^13′(14′)^) are *E*-form, and that between H-8′ and H_2_-11′ suggests *Δ* ^8′(9′)^ double bond is *Z* form. Racemic **4** was submitted to chiral HPLC to afford their enantiomers. The absolute configurations were determined to be 7*S* for (–)-**4** and 7*R* for (+)-**4** by using computational ECD methods (Figure 4).

The NMR data of dimercochlearlactone E (**5**) are similar to those of the known spirocochlealactone A (Qin et al., 2018). Careful analysis of their structures showed that compound **5** is formed by the reduction of nonprotonated carbon (*δ*_C_ 88.3) and a carbonyl group (*δ*_C_ 173.0) in spirocochlealactone A to a methine (*δ*_C_ 40.1) and an aldehyde group (*δ*_C_ 194.0), respectively. The ^1^H-^1^H COSY correlation between H-7′ and H-8′, and the HMBC correlations from H-7′ to C-8′ and C-9′, and from H-8′ to C-9′, C-10′ and C-11′ supports the above conclusions. In the ROESY spectrum, correlations from H-8′ to H-10′, from H-13′ to H-16′, and from H-13 to H-16 indicate that three double bonds (*Δ* ^8′(9′)^, *Δ* ^13′(14′)^, and *Δ* ^13(14)^) are *E*-form. Furthermore, the relative configuration of dimercochlearlactone E (**5**) was assigned as 9R*,7S*, gaining support from the ROESY correlation of Hb-8/H-7′. Dimercochlearlactone E (**5**) was also separated by chiral HPLC to afforded (+)-dimercochlearlactone E (**5**) and (–)-dimercochlearlactone E (**5**). Their absolute configurations were assigned as 9*R*,7*S* for (+)-dimercochlearlactone E (**5**) and 9*S*,7*R* for (–)-dimercochlearlactone E (**5**) by comparing their CD curves with the calculated ones. Thus, the structure of **5** was determined.

Dimercochlearlactone F (**6**) has the molecular formula C_42_H_54_O_7_ based on HRESIMS analysis (*m/z* 783.3727 [M + CF_3_COO]^－^; calcd for 783.3725). The ^1^H NMR spectrum of **6** contains signals for one typical ABX system (*δ*_H_ 7.11, d, *J* = 2.7 Hz, H-3; *δ*_H_ 6.99, dd, *J* = 8.9, 2.7 Hz, H-5; *δ*_H_ 6.80, d, *J* = 8.9 Hz, H-6) and a 1,2,4,5-tetrasubstituted benzene ring (δ_H_ 6.58, s, H-3′ and δ_H_ 7.08 s, H-5′). The ^13^C NMR and DEPT spectra contain the resonances for 42 carbons including 6 methyl, 11 methylene, 10 methine, and 15 nonprotonated carbons (12 aromatic including 4 oxygenated, 1 oxygenated aliphatic, 2 ketones). The above signals suggest that compound **6** is also a meroterpenoid dimer, and its structure consists of parts A and B. The substructure of part A in **6** is similar to that of part A in **1** as they have very similar NMR data. The substructure of part B is very similar to ganomycin F (Cheng et al., 2018). The difference is that the 3-position of the benzene ring in part A is connected to the other additional substructures, which is a hydrogen atom in ganomycin F. The HMBC correlation from H-6′ to C-10 suggests that C-10 is connected to C-5′. Although no HMBC correlations are observed to support C-9-O-C-3 fragment, the presence of ring A is confirmed due to the observation of characteristic chemical shift of C-4 (*δ*_C_ 166.2) in the benzene ring (Ring B). Same phenomenon was observed in other such kind of benzofuran structures, such as cochlearol I and spiroapplanatumines A–Q (Luo et al., 2017; Wang et al., 2019). The ROESY correlations from H-13′ to H-16′ and from H-13 to H-16 indicate that both double bonds (*Δ* ^13′(14′)^ and *Δ* ^13(14)^) are *E*-form. Further correlation from H-8′ to H-11′ observed in ROESY spectrum suggests a *E*-form double bond (*Δ* ^8′(9′)^). Chiral separation by HPLC afforded (+)-**6** and (–)-**6**. Their absolute configurations were determined as 9*S* and 9*R*, respectively, when comparing their experimental CD curves with the calculated ones. As a result, the structure of **6** was assigned.

Dimercochlearlactone G (**7**) has a molecular formula C_42_H_52_O_9_ based on its HRESIMS, ^13^C NMR, and DEPT data. Two ABX aromatic coupling systems at *δ*_H_ 7.07 (d, *J* = 3.0 Hz, H-3), 7.01 (dd, *J* = 8.9, 3.0 Hz, H-5), 6.82 (d, *J* = 8.9 Hz, H-6), 6.35 (d, *J* = 2.9 Hz, H-3′), 6.57 (dd, *J* = 8.6, 2.9 Hz, H-5′), and 6.69 (d, *J* = 8.6 Hz, H-6′) were observed in its ^1^H NMR spectrum. Its ^13^C NMR and DEPT spectra reveal the presence of 42 carbons ascribed to 6 methyl, 9 methylene, 12 methine, and 15 nonprotonated carbons. Analysis of the NMR data of **7** found that part A is similar to dayaolingzhiol K (Zhang et al., 2021). The main difference is that the chemical shift of C-19 is 71.5 ppm in dayaolingzhiol K, while in compound **7** the chemical shift of C-19′ is downfield to 84.5 ppm. In addition, the NMR data of part B resembles that of ganodercin A implying that they have similar structure (Qin et al., 2021). Since no tailing behavior is observed in TLC, the carboxyl group in the structure of part B must be esterified. The structure of **7** is further confirmed by ^1^H NMR, HMBC, and ROESY spectra in DMSO-*d*_6_. In the ^1^H NMR spectrum, there are four free phenolic hydroxyl signals at *δ*_H_ 11.20 (s, 1-OH), 9.18 (s, 4-OH), 9.21 (s, 1′-OH), and 8.77 (s, 4′-OH). The ROESY correlations from 1-OH to H-6, from 4-OH to H-3, from 1′-OH to H-6′, and from 4′-OH to H-3′ fix position of phenolic hydroxyl. Thus, it can be inferred that the carboxyl group can satisfy the requirement of molecular weight only when it is esterified with C-19′. This conjecture was further secured by the ROESY correlation between H_3_-21′ and H_2_-11 and the above-mentioned downfield chemical shift of C-19′. Therefore, the planar structure of **7** was deduced. The ROESY correlation between H-8 and H-11 suggests that the double bond *Δ* ^8(9)^ is *Z*-form configuration. Furthermore, ROESY correlations from H-13 to H-16 and from H-13′ to H-16′ imply *Δ* ^13(14)^ and *Δ* ^13′(14′)^ double bonds are both *E*-form. The absolute configurations of **7** were assigned as 7′*R* for (+)-**7** and 7′*S* for (–)-**7** by comparing experimental CD spectra with those of (+)- and (–)-zizhine A (Cao et al., 2016).

The molecular formula of dimercochlearlactone H (**8**) was assigned as C_42_H_52_O_6_ by the analysis of its HRESIMS. Its ^1^H NMR spectrum contains one ABX aromatic coupling system with the signals at *δ*_H_ 6.57 (d, *J* = 3.0 Hz, H-3), 6.88 (dd, *J* = 8.9, 3.0 Hz, H-5), and 7.12 (d, *J* = 8.9 Hz, H-6). The signals at *δ*_H_ 6.44 (d, *J* = 2.9 Hz, H-3′) and *δ*_H_ 6.77 (d, *J* = 2.9 Hz, H-5′) demonstrate the existence of a 1,3,4,5-tetrasubstituted benzene ring in **8**. Analysis of its ^13^C NMR and DEPT spectra resulted in 42 carbons, including 6 methyl, 10 methylene, 11 methine, and 15 nonprotonated carbons (13 sp2 including 4 oxygenated, 1 oxygenated aliphatic, and 1 carbonyl). Compound **8** is a meroterpenoids dimer consisting of two parts. The data of part A resemble those of ganomycin I (El Dine et al., 2009); the difference is that C-7 (*δ*_C_ 81.4) is a nonprotonated carbon in **8**. The HMBC correlations from H-3 and H-8 to C-7 and from H-8 to C-9 and C-10 support the structure of part A. The structure of part B is very similar to ganomycin F, with the difference appearing at C-2′ being a nonprotonated carbon. The HMBC correlations from H-3′ to C-7 and from H-8 to C-2′ suggest that C-7 is connected to C-2′. The molecular weight and the unsaturation of the molecule need to form another ring to be satisfied. There are three possible ring formations, which are C-1-O-C-1′ C-1′-O-C-10 or C-1-O-C-10. In the ^1^H NMR spectrum (DMSO-*d*_6_), there are two free phenolic hydroxyl signals at *δ*_H_ 9.42 (s, 4-OH), 9.29 (s, 4′-OH). The ROESY correlations from 4-OH to H-3 and H-5 and from 4′-OH to H-3′ and H-5′ fix phenolic hydroxyl at C-4′ and C-4′, confirming the formation of ring C. Furthermore, the ROESY correlations from H-13′ to H-16′ and from H-13 to H-16 suggest that double bonds *Δ* ^13′(14′)^ and *Δ* ^13(14)^ are *E*-form. Additional ROESY correlation between H-8′ and H-11′ suggests a *E*-form double bond *Δ* ^8′(9′)^. The absolute configurations of **8** were determined to be 7*R* for (+)-**8** and 7*S* for (–)-**8** by using ECD calculation methods.

The molecular formula of **9** was specified as C_32_H_34_O_8_ based on the analysis of its positive HRESIMS ([M + Na]^+^, m/z 569.2154, calcd 569.2151). The ^1^H NMR spectrum exhibits two typical ABX spin systems *δ*_H_ 7.14, d, *J* = 2.9 Hz, H-3; 7.08, dd, *J* = 8.9, 2.9 Hz, H-5; 6.84 d, *J* = 8.9 Hz, H-6; *δ*_H_ 6.98, d, *J* = 8.9 Hz, H-6′; 6.85, dd, *J* = 8.9, 2.9 Hz, H-5′; 6.45, d, *J* = 2.9 Hz, H-3′. The ^13^C NMR and DEPT spectra of **9** display 4 methyl, 5 methylene, 9 olefifinic methine, 14 nonprotonated carbons (11 aromatic including 4 oxygenated, 2 carbonyl, and a ketone group). These data are very similar to those of spirocochlealactone B (Qin et al., 2018). The difference between **9** and spirocochlealactone B is that a sesquiterpenoid residue in spirocochlealactone B is disappeared instead of a monoterpenoid residue in **9**. This inference is supported by the ^1^H-^1^H COSY correlations from H_2_-12′ to H_2_-11′ and H-13′ and the HMBC correlations from H_3_-15′ and H_3_-16′ to C-13′ and C-14′, from H-8′ to C-9′, C-10′ and C-11′, and from H-11′ to C-12′ and C-10′. The relative configurations at the chiral centers were determined to be 9S*,7′S* based on the observation of the ROESY correlation H-8′(*δ*_H_ 7.06)/Ha-11 (*δ*_H_ 2.21). The absolute configurations of **9** were determined as 9*R*,7′*R* for (+)-**9** and 9*S*,7′*S* for (−)-**9** by comparing the experimental CD spectra with those of (+)-spirocochlealactone B and (−)-spirocochlealactone B (Qin et al., 2018). Thus, the structure of **9** was identified and named as dimercochlearlactone I.

The molecular formula of compound dimercochlearlactone J (**10**) is similar to that of **9**. Careful examination of 2D NMR (see Supplementary Figures S81–S84, S91−S94) data between **10** and **9** indicates that they have same planar structure. The observed ROESY correlation (see Supplementary Figure S94) of H-8′ (*δ*_H_ 7.16)/H-8 (*δ*_H_ 3.68, 3.07) in **10** suggests 9R*,7*S*′* relative configurations at chiral centers. The absolute configurations of **10** were determined as 9*S*,7′*R* for (+)-dimercochlearlactone J (**10**) and 9*R*,7′*S* for (−)-dimercochlearlactone J (**10**) by comparing their experimental CD spectra to (+)-spirocochlealactone A and (−)-spirocochlealactone A (Qin et al., 2018). Thus, the structure of **10** was assigned.

Compound **11** was identified as spirocochlealactone A by comparing its NMR and MS data with the literature data (Qin et al., 2018). This compound has been isolated by us from 10 kg of *G. cochlear*, and in this experiment, it was isolated from *G. lucidum*.

**FIGURE 6:**
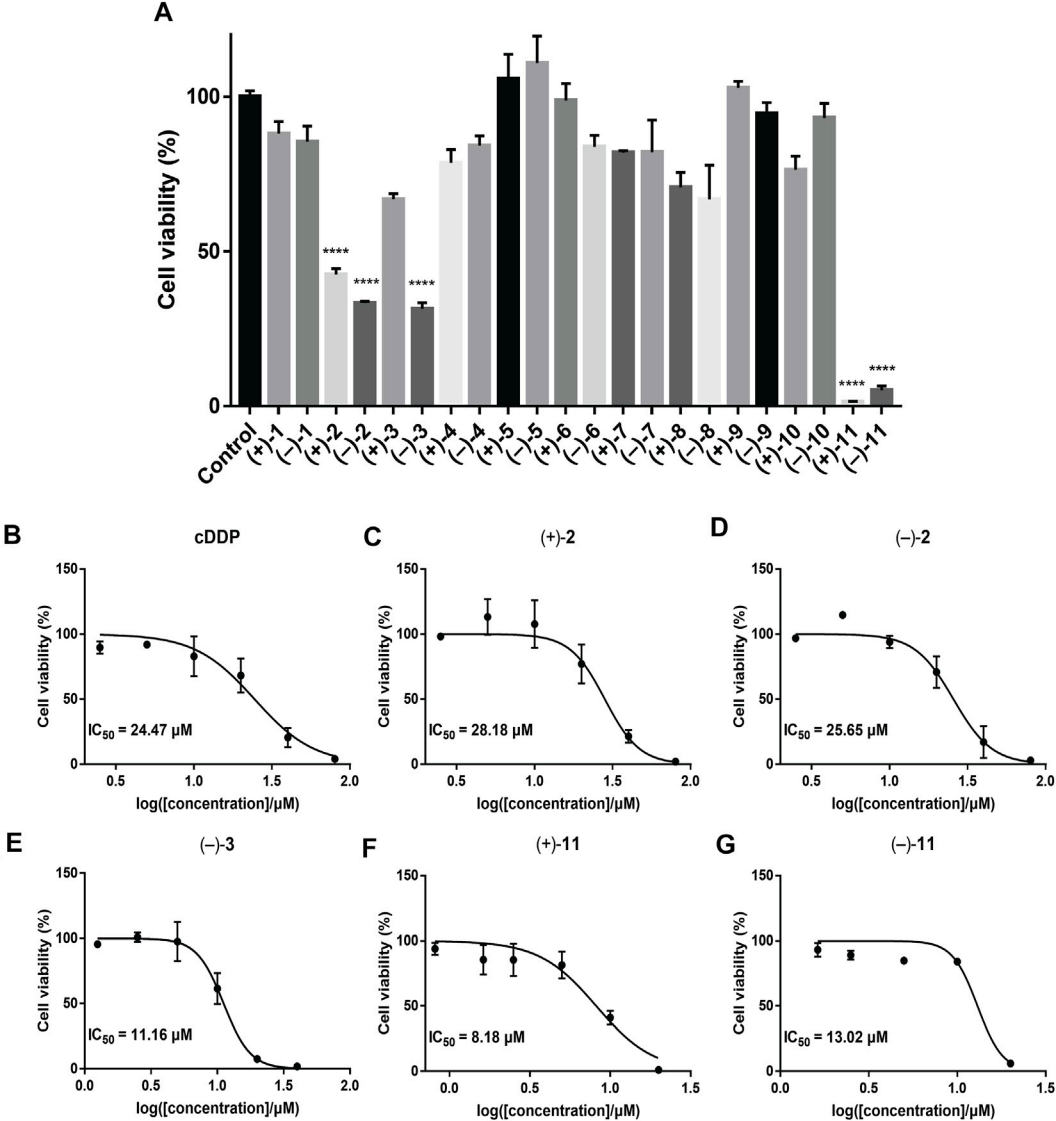
Cell viability of the compounds in MDA-MB-231 cells by CCK-8 assay. The results are represented as mean ± SD of triplicates. ****p < 0.0001 vs. control group. **(A)** Primary screening at 20 μM. **(B–G)** Dose-response curves of cisplatin (positive control) and the tested compounds showing IC_50_ values.

**FIGURE 7:**
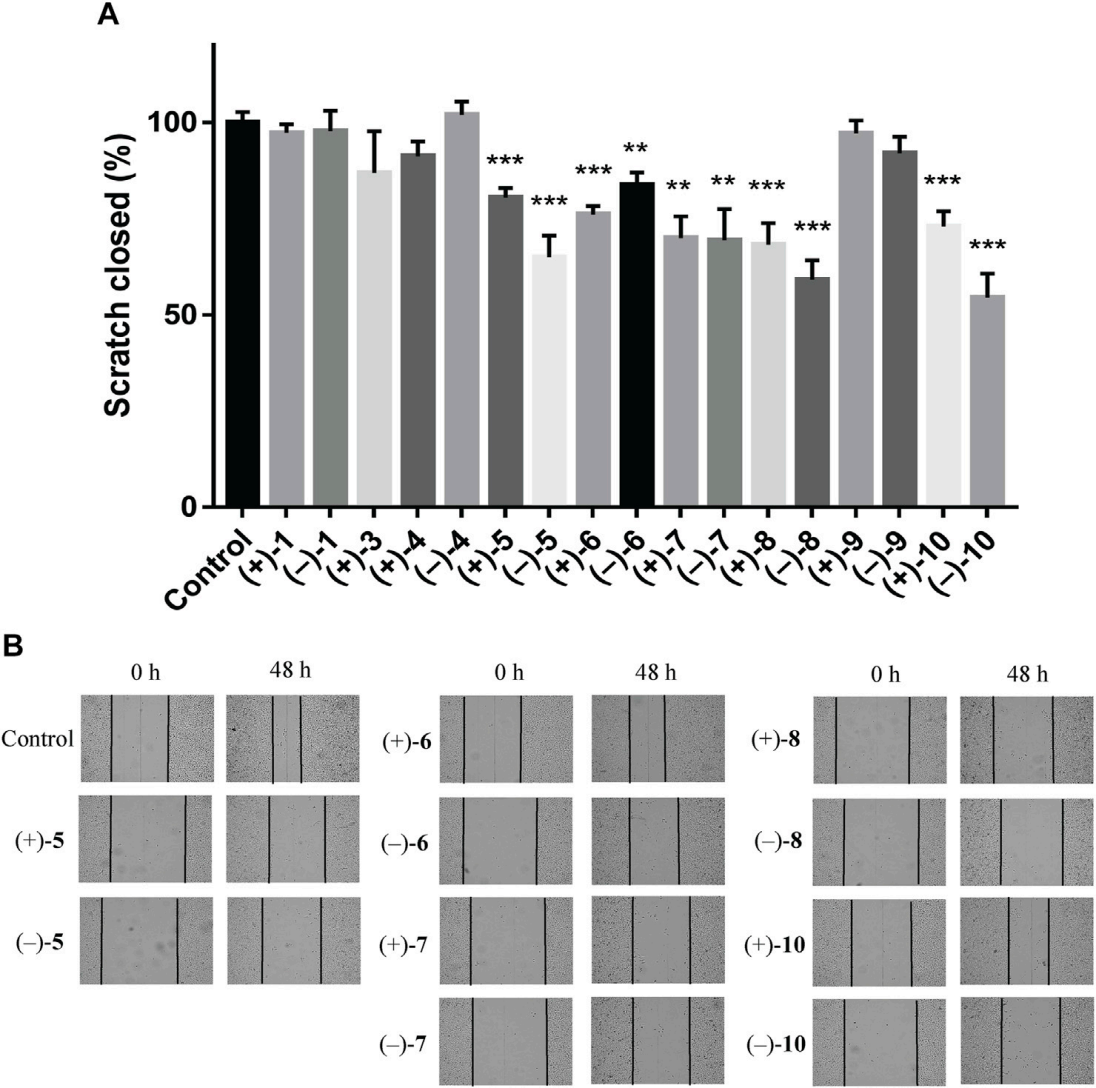
Compounds suppress the migratory ability of MDA-MB-231 cells by wound healing assay. **(A)** Quantification of the migratory ability of compounds against MDA-MB-231 cells at 20 μM. The results were represented as mean ± SD of triplicates. **p < 0.01, ***p < 0.001 (vs. control group). **(B)** Representative images of the wound healing assay in MDA-MB-231 cells.

To investigate the anti-TNBC effects of the isolated compounds, we used the MDA-MB-231 cells for our analyses. All 22 dimer meroterpenoid enantiomers were evaluated for their suppressive effect toward MDA-MB-231 cells. It was found that (+)**-2**, (–)-**2**, (–)-**3**, (+)**-11**, and (–)-**11** significantly decreased the cell viability in MDA-MB-231 cells (Figure 6A). Moreover, the morphological and density changes were observed in MDA-MB-231 cells upon exposure of the compounds (Supplementary Figure S98). To further analyze the effects of the compounds on MDA-MB-231 cells, the dose-response studies were performed. The cell viability of MDA-MB-231 cells was substantially inhibited by treatment with compounds for 48 h in a dose-dependent manner. As shown in Figure 6B, similar to the positive control (cisplatin), compound treatment dose-dependently inhibits MDA-MB-231 cells growth. The IC_50_ of compounds for MDA-MB-231 cells are 28.18, 25.65, 11.16, 8.18, and 13.02 μM, respectively. The remaining compounds showed negligible inhibitory effects on cell viability at 20 μM (Figure 6A). Interestingly, although all the remaining compounds have rather low cytotoxicities toward MDA-MB-231 cells, ten of the isolates (+)**-5**, (–)-**5**, (+)**-6**, (–)-**6**, (+)**-7**, (–)-**7**, (+)**-8**, (–)-**8**, (+)**-10**, and (–)-**10** significantly inhibit the migration ability of MDA-MB-231 cells (Figure 7), suggesting that they might be promising lead compounds for the development of anti-cancer drugs against metastasis of TNBC.

## Conclusion

To conclude, this study resulted in the isolation of ten pairs of novel meroterpenid dimers and one pair of known compounds from *Ganoderma* species. Biological results revealed the importance of (+)**-2**, (–)-**2**, (–)-**3**, (+)**-11**, and (–)-**11** in the development of the anti-TNBC drugs. Furthermore, (+)**-5**, (–)-**5**, (+)**-6**, (–)-**6**, (+)**-7**, (–)-**7**, (+)**-8**, (–)-**8**, (+)**-10**, and (–)-**10** significantly inhibit the migration ability of MDA-MB-231 cells, thereby providing promising compounds for the development of anti-TNBC drugs.

## Data Availability

The original contributions presented in the study are included in the article/Supplementary Material, further inquiries can be directed to the corresponding author.

## References

[B1] Cai D. Zhang J. J. Wu Z. H. Qin F. Y. Yan Y. M. Zhang M. (2021). Lucidumones B-H, Racemic Meroterpenoids That Inhibit Tumor Cell Migration from *Ganoderma lucidum* . Bioorg. Chem. 110, 104774. 10.1016/j.bioorg.2021.104774 33711656

[B2] Cao W. W. Luo Q. Cheng Y. X. Wang S. M. (2016). Meroterpenoid Enantiomers from *Ganoderma sinensis* . Fitoterapia 110, 110–115. 10.1016/j.fitote.2016.03.003 26947249

[B3] Chen H. Yang J. P. Yang Y. L. Zhang J. P. Xu Y. Lu X. L. (2021). The Natural Products and Extracts: Anti-triple-negative Breast Cancer *In Vitro* . Chem. Biodivers 18, e2001047. 10.1002/cbdv.202001047 34000082

[B4] Cheng L. Z. Qin F. Y. Ma X. C. Wang S. M. Yan Y. M. Cheng Y. X. (2018). Cytotoxic and *N*-Acetyltransferase Inhibitory Meroterpenoids from *Ganoderma cochlear* . Molecules 23, 1797. 10.3390/molecules23071797 PMC6100301 30037018

[B5] Chowdhury P. Ghosh U. Samanta K. Jaggi M. Chauhan S. C. Yallapu M. M. (2021). Bioactive Nanotherapeutic Trends to Combat Triple Negative Breast Cancer. Bioact. Mater. 6, 3269–3287. 10.1016/j.bioactmat.2021.02.037 33778204 PMC7970221

[B6] El Dine R. S. El Halawany A. M. Ma C. M. Hattori M. (2009). Inhibition of the Dimerization and Active Site of HIV-1 Protease by Secondary Metabolites from the Vietnamese Mushroom *Ganoderma colossum* . J. Nat. Prod. 72, 2019–2023. 10.1021/np900279u 19813754

[B7] Lin Z. Deng A. (2019). Antioxidative and Free Radical Scavenging Activity of *Ganoderma* (Lingzhi). Adv. Exp. Med. Biol. 1182, 271–297. 10.1007/978-981-32-9421-9_12 31777024

[B8] Lin Z. Sun L. (2019). Antitumor Effect of *Ganoderma* (Lingzhi) Mediated by Immunological Mechanism and its Clinical Application. Adv. Exp. Med. Biol. 1182, 39–77. 10.1007/978-981-32-9421-9_2 31777014

[B9] Liu Q. Tie L. (2019). Preventive and Therapeutic Effect of *Ganoderma* (Lingzhi) on Diabetes. Adv. Exp. Med. Biol. 1182, 201–215. 10.1007/978-981-32-9421-9_8 31777020

[B10] Luo Q. Wei X. Y. Yang J. Luo J. F. Liang R. Tu Z. C. (2017). Spiro Meroterpenoids from *Ganoderma applanatum* . J. Nat. Prod. 80, 61–70. 10.1021/acs.jnatprod.6b00431 27996259

[B11] Luo Q. Li M. K. Luo J. F. Tu Z. C. Cheng Y. X. (2018). COX-2 and JAK3 Inhibitory Meroterpenoids from the Mushroom *Ganoderma theaecolum* . Tetrahedron 74, 4259–4265. 10.1016/j.tet.2018.06.053

[B12] Meng J. Yang B. (2019). Protective Effect of *Ganoderma* (Lingzhi) on Cardiovascular System. Adv. Exp. Med. Biol. 1182, 181–199. 10.1007/978-981-32-9421-9_7 31777019

[B13] Niu X. M. Li S. H. Sun H. D. Che C. T. (2006). Prenylated Phenolics from *Ganoderma fornicatum* . J. Nat. Prod. 69, 1364–1365. 10.1021/np060218k 16989537

[B14] O'Reilly D. Sendi M. A. Kelly C. M. (2021). Overview of Recent Advances in Metastatic Triple Negative Breast Cancer. Wjco 12, 164–182. 10.5306/wjco.v12.i3.164 33767972 PMC7968109

[B15] Qin F. Y. Yan Y. M. Tu Z. C. Cheng Y. X. (2018). Meroterpenoid Dimers from *Ganoderma cochlear* and Their Cytotoxic and COX-2 Inhibitory Activities. Fitoterapia 129, 167–172. 10.1016/j.fitote.2018.06.019 29969649

[B16] Qin F. Y. Zhang J. J. Wang D. W. Xu T. Cai D. Cheng Y. X. (2021). Direct Determination of *E* and *Z* Configurations for Double Bond in Bioactive Meroterpenoids from *Ganoderma* Mushrooms by Diagnostic ^1^H NMR Chemical Shifts and Structure Revisions of Previous Analogues. J. Funct. Foods 87, 104758. 10.1016/j.jff.2021.104758

[B17] Quan Y. Ma A. Yang B. (2019). Preventive and Therapeutic Effect of *Ganoderma* (Lingzhi) on Brain Injury. Adv. Exp. Med. Biol. 1182, 159–180. 10.1007/978-981-32-9421-9_6 31777018

[B18] Wang X. L. Wu Z. H. Di L. Zhou F. J. Yan Y. M. Cheng Y. X. (2019). Renoprotective Meroterpenoids from the Fungus *Ganoderma cochlear* . Fitoterapia 132, 88–93. 10.1016/j.fitote.2018.12.002 30521858

[B19] Zhang J. J. Wang D. W. Cai D. Lu Q. Cheng Y. X. (2021). Meroterpenoids from *Ganoderma lucidum* Mushrooms and Their Biological Roles in Insulin Resistance and Triple Negative Breast Cancer. Front. Chem. 9, 772740. 10.3389/fchem.2021.772740 34805099 PMC8595597

