# Supplementary material for: Meroterpenoid Dimers from Ganoderma Mushrooms and Their Biological Activities Against Triple Negative Breast Cancer Cells
Source: Front Chem. 2022 May 3;10:888371. doi: 10.3389/fchem.2022.888371 (PMC9111535; doi:10.3389/fchem.2022.888371)
Supplement: Supplementary file 1 [file DataSheet1.doc]

Supplementary Material

# Meroterpenoid dimers from *Ganoderma* mushrooms and their biological activities against triple negative breast cancer cells

Fu-Ying Qin^1†^, Yan-Yi Chen^1†^, Jiao-Jiao Zhang^1^, Yong-Xian Cheng^1,2^*

^1^Institute for Inheritance-Based Innovation of Chinese Medicine, School of Pharmaceutical Sciences, Health Science Center, Shenzhen University, Shenzhen 518060, China.

^2^Guangdong Key Laboratory for Functional Substances in Medicinal Edible Resources and Healthcare Products, School of Life Sciences and Food Engineering, Hanshan Normal University, Chaozhou 521041, China

*** Correspondence:** Yong-Xian Cheng

email: [yxcheng@szu.edu.cn](mailto:yxcheng@szu.edu.cn)

^†^ These authors contributed equally to this paper

**Contents**

**Table S1.** ^1^H (600 MHz) and ^13^C NMR (150 MHz) data of **1** in methanol-*d*_4_ (*δ* in ppm, *J* in Hz)

**Table S2.** ^1^H (600 MHz) and ^13^C NMR (150 MHz) data of **2** in methanol-*d*_4_ (*δ* in ppm, *J* in Hz)

**Figure S1.** ^1^H NMR (500 MHz) spectrum of **1** in DMSO-*d*_6_.

**Figure S2.** ^13^C NMR (125 MHz) spectrum of **1** in DMSO-*d*_6_.

**Figure S3.** HSQC spectrum of **1** in DMSO-*d*_6_.

**Figure S4.** HMBC spectrum of **1** in DMSO-*d*_6_.

**Figure S5.** ^1^H-^1^H COSY spectrum of **1** in DMSO-*d*_6_.

**Figure S6.** ROESY spectrum of **1** in DMSO-*d*_6_.

**Figure S7.** ^1^H NMR (600 MHz) spectrum of compound **1** in methanol-*d*_4_.

**Figure S8.** ^13^C NMR (150 MHz) spectra and DEPT of compound **1** in methanol-*d*_4_.

**Figure S9.** HSQC spectrum of compound **1** in methanol-*d*_4_.

**Figure S10.** HMBC spectrum of compound **1** in methanol-*d*_4_.

**Figure S11.** ^1^H-^1^H COSY spectrum of compound **1** in methanol-*d*_4_.

**Figure S12.** ROESY spectrum of compound **1** in methanol-*d*_4_.

**Figure S13.** HRESIMS of **1**.

**Figure S14.** ^1^H NMR (500 MHz) spectrum of **2** in DMSO-*d*_6_.

**Figure S15.** ^13^C NMR (125 MHz) spectrum of **2** in DMSO-*d*_6_.

**Figure S16.** HSQC spectrum of **2** in DMSO-*d*_6_.

**Figure S17.** HMBC spectrum of **2** in DMSO-*d*_6_.

**Figure S18.** Enlarge HMBC spectrum of compound **2** in DMSO-*d*_6_.

**Figure S19.** ^1^H-^1^H COSY spectrum of compound **2** in DMSO-*d*_6_.

**Figure S20.** ROESY spectrum of **2** in DMSO-*d*_6_.

**Figure S21.** ^1^H NMR (600 MHz) spectrum of compound **2** in methanol-*d*_4_.

**Figure S22.** ^13^C NMR (150 MHz) spectra and DEPT of compound **2** in methanol-*d*_4_.

**Figure S23.** HSQC spectrum of compound **2** in methanol-*d*_4_.

**Figure S24.** HMBC spectrum of compound **2** in methanol-*d*_4_.

**Figure S25.** ^1^H-^1^H COSY spectrum of compound **2** in methanol-*d*_4_.

**Figure S26.** ROESY spectrum of compound **2** in methanol-*d*_4_.

**Figure S27.** HRESIMS of **2**.

**Figure S28.** ^1^H NMR (600 MHz) spectrum of compound **3** in methanol-*d*_4_.

**Figure S29.** ^13^C NMR (150 MHz) spectra and DEPT of compound **3** in methanol-*d*_4_.

**Figure S30.** HSQC spectrum of compound **3** in methanol-*d*_4_.

**Figure S31.** HMBC spectrum of compound **3** in methanol-*d*_4_.

**Figure S32.** ^1^H-^1^H COSY spectrum of compound **3** in methanol-*d*_4_.

**Figure S33.** ROESY spectrum of compound **3** in methanol-*d*_4_.

**Figure S34.** ^1^H NMR (600 MHz) spectrum of compound **3** in DMSO-*d*_6_.

**Figure S35.** ROESY spectrum of compound **3** in DMSO-*d*_6_.

**Figure S36.** Enlarge ROESY spectrum of compound **3** in DMSO-*d*_6_.

**Figure S37.** HRESIMS of **3**.

**Figure S38.** ^1^H NMR (600 MHz) spectrum of compound **4** in methanol-*d*_4_.

**Figure S39.** ^13^C NMR (150 MHz) and DEPT spectra of compound **4** in methanol-*d*_4_.

**Figure S40.** HSQC spectrum of compound **4** in methanol-*d*_4_.

**Figure S41.** HMBC spectrum of compound **4** in methanol-*d*_4_.

**Figure S42.** COSY spectrum of compound **4** in methanol-*d*_4_.

**Figure S43.** ROESY spectrum of compound **4** in methanol-*d*_4_.

**Figure S44.** HRESIMS of **4**.

**Figure S45.** ^1^H NMR (600 MHz) spectrum of compound **5** in CDCl_3_.

**Figure S46.** ^13^C NMR (150 MHz) spectra of compound **5** in CDCl_3_.

**Figure S47.** HSQC spectrum of compound **5** in CDCl_3_.

**Figure S48.** HMBC spectrum of compound **5** in CDCl_3_.

**Figure S49.** COSY spectrum of compound **5** in CDCl_3_.

**Figure S50.** ROESY spectrum of compound **5** in CDCl_3_.

**Figure S51.** HRESIMS of **5**.

**Figure S52.** ^1^H NMR (600 MHz) spectrum of compound **6** in CDCl_3_.

**Figure S53.** ^13^C NMR (150 MHz) and DEPT spectra of compound **6** in CDCl_3_.

**Figure S54.** HSQC spectrum of compound **6** in CDCl_3_.

**Figure S55.** HMBC spectrum of compound **6** in CDCl_3_.

**Figure S56.** COSY spectrum of compound **6** in CDCl_3_.

**Figure S57.** ROESY spectrum of compound **6** in CDCl_3_.

**Figure S58.** Enlarge ROESY spectrum of compound **6** in CDCl_3_.

**Figure S59.** HRESIMS of **6**.

**Figure S60.** ^1^H NMR (500 MHz) spectrum of compound **7** in DMSO-*d*_6_.

**Figure S61.** ^13^C NMR (125 MHz) and DEPT spectra of compound **7** in DMSO-*d*_6_.

**Figure S62.** HSQC spectrum of compound **7** in DMSO-*d*_6_.

**Figure S63.** HMBC spectrum of compound **7** in DMSO-*d*_6_.

**Figure S64.** COSY spectrum of compound **7** in DMSO-*d*_6_.

**Figure S65.** ROESY spectrum of compound **7** in DMSO-*d*_6_.

**Figure S66.** HRESIMS of **7**.

**Figure S67**. CD spectrum of (+)-**7**.

**Figure S68**. CD spectrum of (–)-**7**.

**Figure S69.** ^1^H NMR (600 MHz) spectrum of compound **8** in CDCl_3_.

**Figure S70.** ^13^C NMR (150 MHz) and DEPT spectra of compound **8** in CDCl_3_.

**Figure S71.** HSQCspectrum of compound **8** in CDCl_3_.

**Figure S72.** HMBC spectrum of compound **8** in CDCl_3_.

**Figure S73.** COSY spectrum of compound **8** in CDCl_3_.

**Figure S74.** ROESY spectrum of compound **8** in CDCl_3_.

**Figure S75.** ^1^H NMR spectrum of compound **8** in DMSO-*d*_6_.

**Figure S76.** ROESY spectrum of compound **8** in DMSO-*d*_6_.

**Figure S77.** Enlarge ROESY spectrum of compound **8** in DMSO-*d*_6_.

**Figure S78**. HRESIMS of **8**.

**Figure S79.** ^1^H NMR (500 MHz) spectrum of compound **9** in CDCl_3_.

**Figure S80.** ^13^C NMR (125 MHz) and DEPT spectra of compound **9** in CDCl_3_.

**Figure S81.** HSQC spectrum of compound **9** in CDCl_3_.

**Figure S82.** HMBC spectrum of compound **9** in CDCl_3_.

**Figure S83.** COSY spectrum of compound **9** in CDCl_3_.

**Figure S84.** ROESY spectrum of compound **9** in CDCl_3_.

**Figure S85.** Enlarge ROESY spectrum of compound **9** in CDCl_3_.

**Figure S86.** HRESIMS of **9**.

**Figure S87.** CD spectrum of (+)-**9**.

**Figure S88.** CD spectrum of (–)-**9**.

**Figure S89.** ^1^H NMR (500 MHz) spectrum of compound **10** in CDCl_3_.

**Figure S90.** ^13^C NMR (125 MHz) and DEPT spectra of compound **10** in CDCl_3_.

**Figure S91.** HSQC spectrum of compound **10** in CDCl_3_.

**Figure S92.** HMBC spectrum of compound **10** in CDCl_3_.

**Figure S93.** COSY spectrum of compound **10** in CDCl_3_.

**Figure S94.** ROESY spectrum of compound **10** in CDCl_3_.

**Figure S95.** HRESIMS of **10**.

**Figure S96**. CD spectrum of (+)-**10**.

**Figure S97**. CD spectrum of (–)-**10**.

**Figure S98**. Representative images of the cell viability assay in MDA-MB-231 cells.

**Table S1.** 1H (600 MHz) and 13C NMR (150 MHz) data of **1** in methanol-*d*4 (*δ* in ppm, *J* in Hz)

| No. | *δ*_H_ | *δ*_C_ |  | No. | *δ*_H_ | *δ*_C_ |
| --- | --- | --- | --- | --- | --- | --- |
| 1 |  | 156.6 s |  | 1' |  | 156.3 s |
| 2 |  | 120.5 s |  | 2' |  | 123.8 s |
| 3 | 7.22, d (2.9) | 115.9 d |  | 3' | 7.12, d (2.9) | 116.6 d |
| 4 |  | 150.7 d |  | 4' |  | 141.9 d |
| 5 | 7.03, dd (8.9, 2.9) | 126.3 d |  | 5' | 6.76, dd (8.9, 2.9) | 116.1 d |
| 6 | 6.80, d (8.9) | 119.8 d |  | 6' | 6.94, d (8.9) | 124.7 d |
| 7 |  | 201.7 C |  | 7' |  | 118.0 s |
| 8 | Ha 3.90, d (17.2)  Hb 3.56, d (17.2) | 45.4 t |  | 8' | 8.58, s | 176.5 d |
| 9 |  | 90.3 s |  | 9' |  | 173.8 s |
| 10 |  | 205.4 s |  | 10' | 2.54, t (7.4) | 35.4 t |
| 11 | Ha 2.00, m  Hb 1.89, m | 38.1 t |  | 11' | 2.37, q (7.4) | 24.4 t |
| 12 | 2.08, m | 22.4 t |  | 12' | 5.14, t-like (7.0) | 123.5 d |
| 13 | 5.11, t (6.9) | 123.8 d |  | 13' |  | 134.3 s |
| 14 |  | 137.7 s |  | 14' | 1.62, s | 17.8 q |
| 15 | 1.58, s | 16.2 q |  | 15' | 1.68, s | 25.8 q |
| 16 | 1.97, m | 40.7 t |  |  |  |  |
| 17 | 2.07, m | 27.6 t |  |  |  |  |
| 18 | 5.09, t (6.9) | 125.3 d |  |  |  |  |
| 19 |  | 132.3 s |  |  |  |  |
| 20 | 1.60, s | 17.8 q |  |  |  |  |
| 21 | 1.69, s | 25.9 q |  |  |  |  |

**Table S2.** 1H (600 MHz) and 13C NMR (150 MHz) data of **2** (*δ* in ppm, *J* in Hz)

| No. | *δ*_H_ | *δ*_C_ |  | No. | *δ*_H_ | *δ*_C_ |
| --- | --- | --- | --- | --- | --- | --- |
| 1 |  | 156.7 s |  | 1' |  | 156.3 s |
| 2 |  | 120.6 s |  | 2' |  | 123.8 s |
| 3 | 7.21, d (2.9) | 115.9 d |  | 3' | 7.10, d (2.9) | 116.7 d |
| 4 |  | 150.8 s |  | 4' |  | 141.9 s |
| 5 | 7.01, dd (8.9, 2.9) | 126.4 d |  | 5' | 6.74, dd (8.9, 2.9) | 116.1 d |
| 6 | 6.78, d (8.9) | 119.8 d |  | 6' | 6.92, d (8.9) | 124.7 d |
| 7 |  | 201.8 C |  | 7' |  | 118.0 C |
| 8 | Ha: 3.88, d (17.4)  Hb: 3.54, d (17.4) | 44.8 t |  | 8' | 8.56, s | 176.5 d |
| 9 |  | 90.3 s |  | 9' |  | 173.8 s |
| 10 |  | 205.5 s |  | 10' | 2.52, t (7.4) | 35.4 t |
| 11 | Ha: 1.95, m  Hb: 1.86, m | 38.0 t |  | 11' | 2.35, q (7.4) | 24.4 t |
| 12 | 2.05, m | 22.4 t |  | 12' | 5.14, t-like (7.0) | 123.5 d |
| 13 | 5.09, t (6.9) | 123.8 d |  | 13' |  | 138.0 s |
| 14 |  | 137.7 s |  | 14' | 1.61, s | 16.2 q |
| 15 | 1.58, s | 16.2 q |  | 15' | 1.97, overlap | 40.7 t |
| 16 | 1.97, overlap | 40.8 t |  | 16' | 2.05, overlap | 27.7 t |
| 17 | 2.05, overlap | 27.7 t |  | 17' | 5.07, overlap | 125.3 d |
| 18 | 5.07, overlap | 125.3 d |  | 18' |  | 132.3 s |
| 19 |  | 132.3 s |  | 19' | 1.57, s | 17.8 q |
| 20 | 1.57, s | 17.8 q |  | 20' | 1.64, s | 25.9 q |
| 21 | 1.64, s | 25.9 q |  |  |  |  |


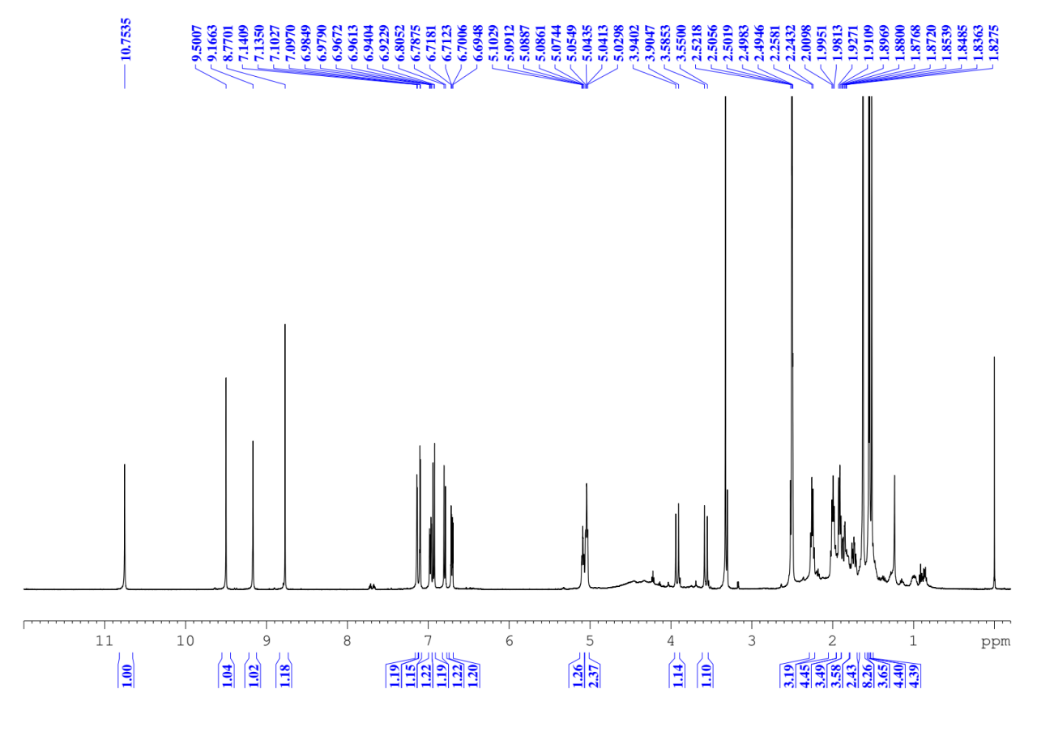


**Figure S1.** ^1^H NMR (500 MHz) spectrum of compound **1** in DMSO-*d*_6_.


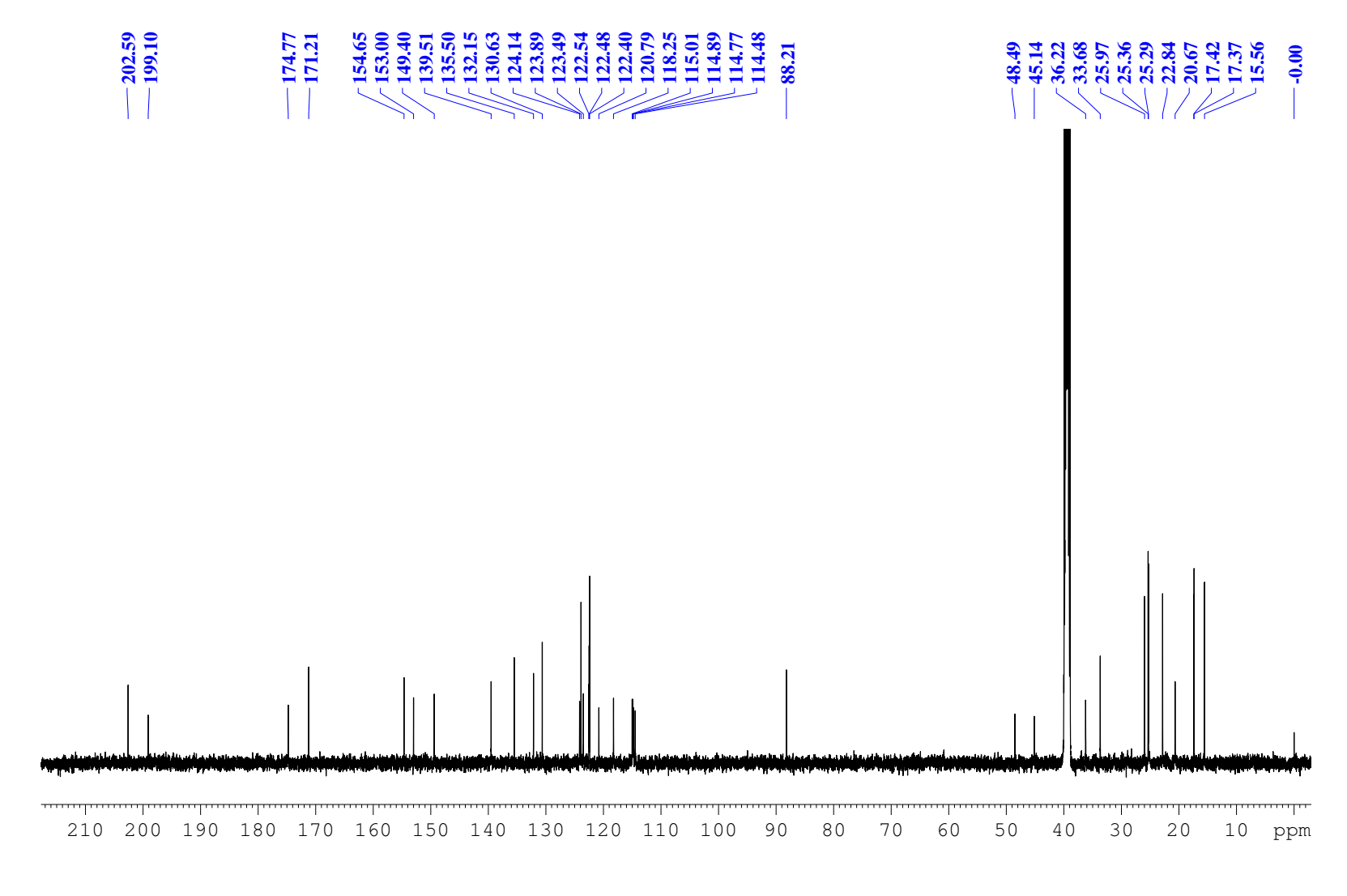


**Figure S2.** ^13^C NMR (125 MHz) spectrum of compound **1** in DMSO-*d*_6_.


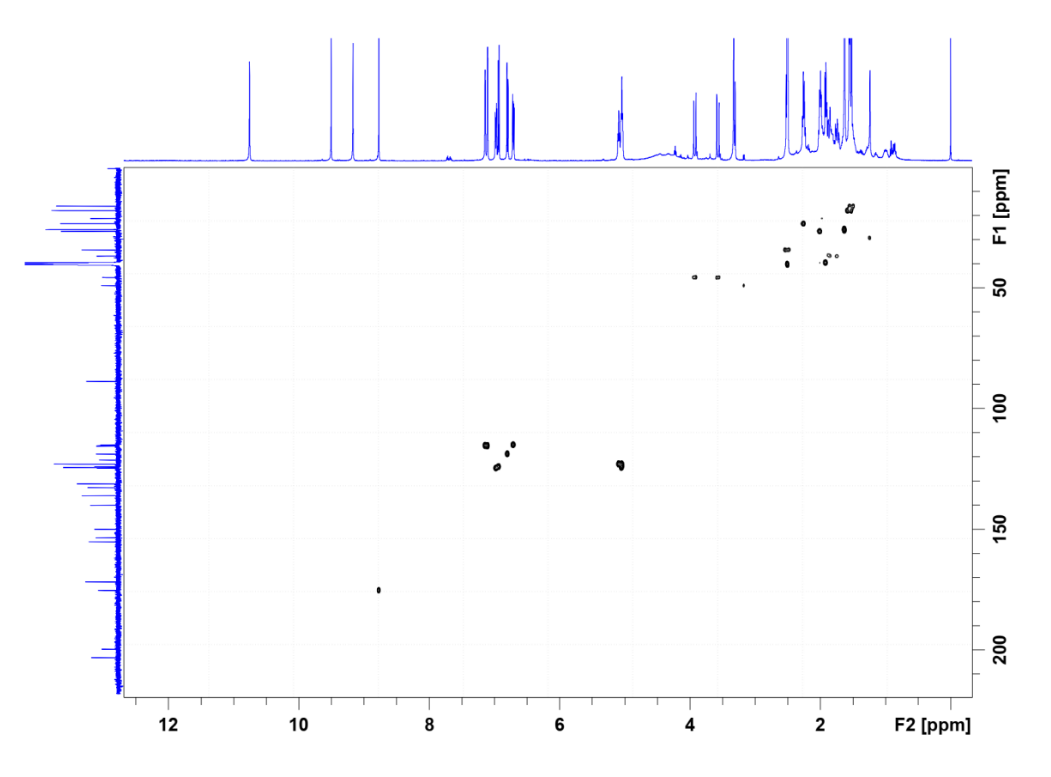


**Figure S3.** HSQC spectrum of compound **1** in DMSO-*d*_6_.


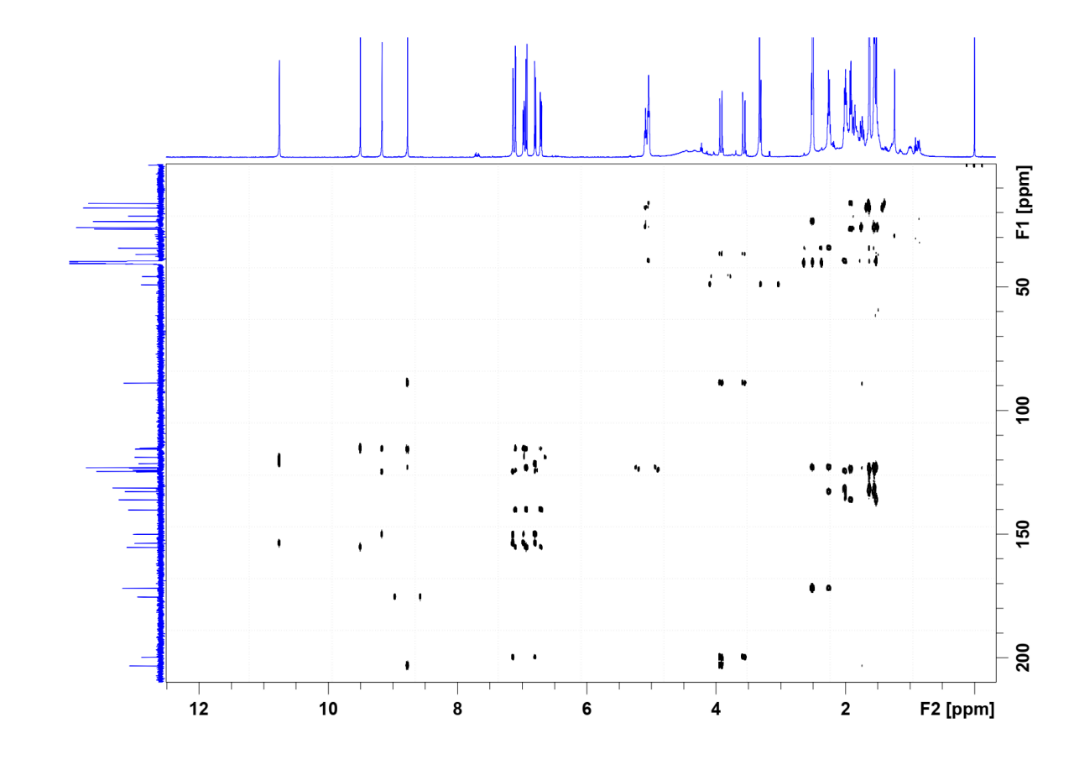


**Figure S4.** HMBC spectrum of compound **1** in DMSO-*d*_6_.


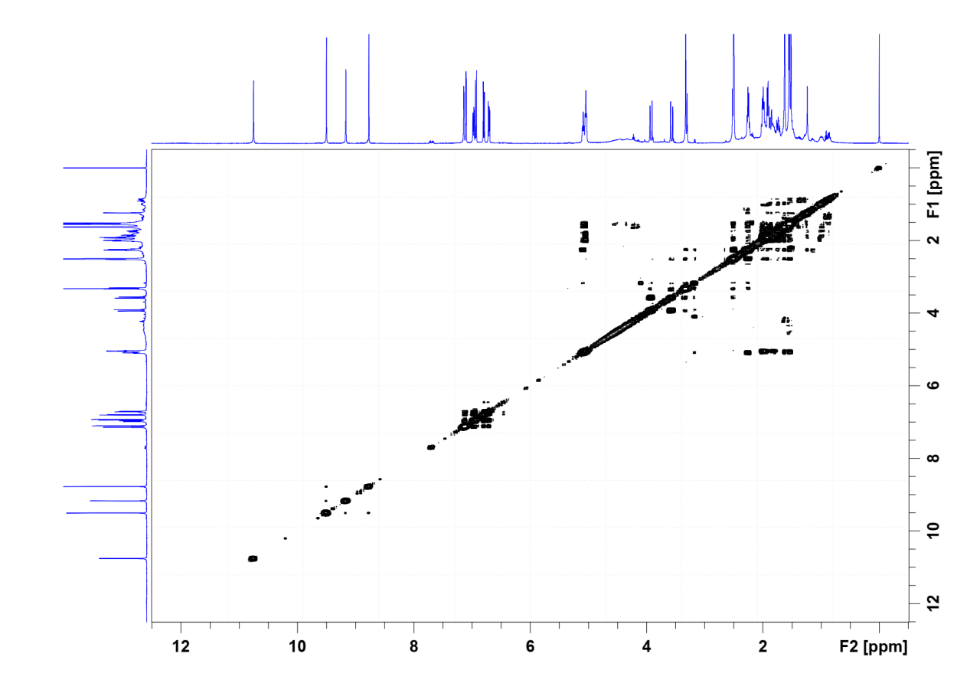


**Figure S5.** ^1^H-^1^H COSY spectrum of compound **1** in DMSO-*d*_6_.


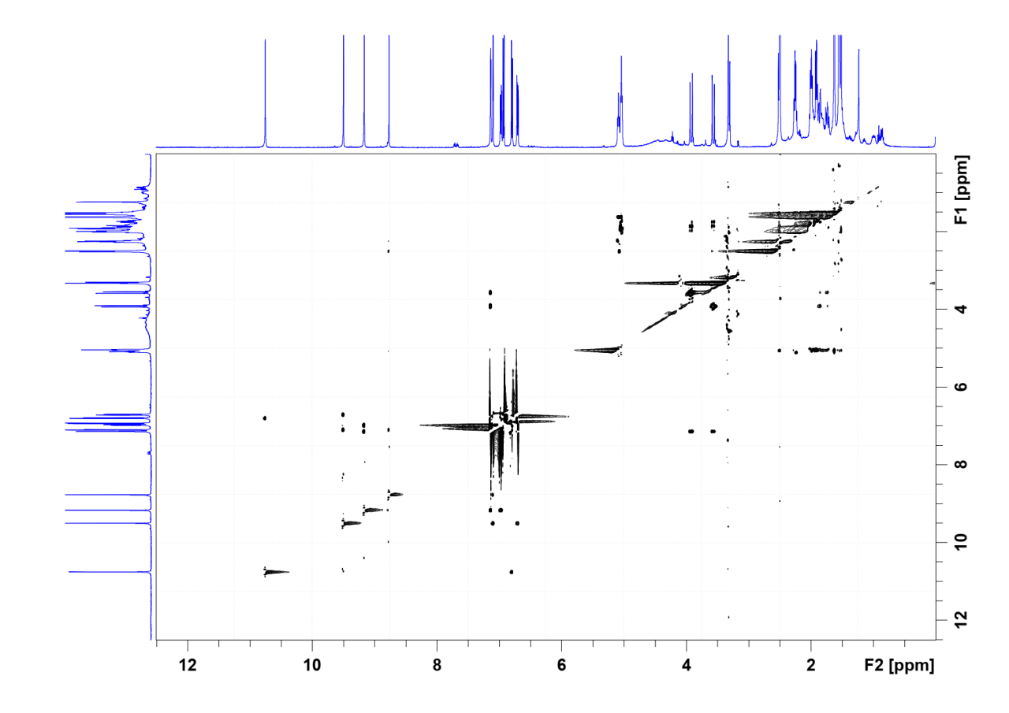


**Figure S6.** ROESY spectrum of compound **1** in DMSO-*d*_6_.


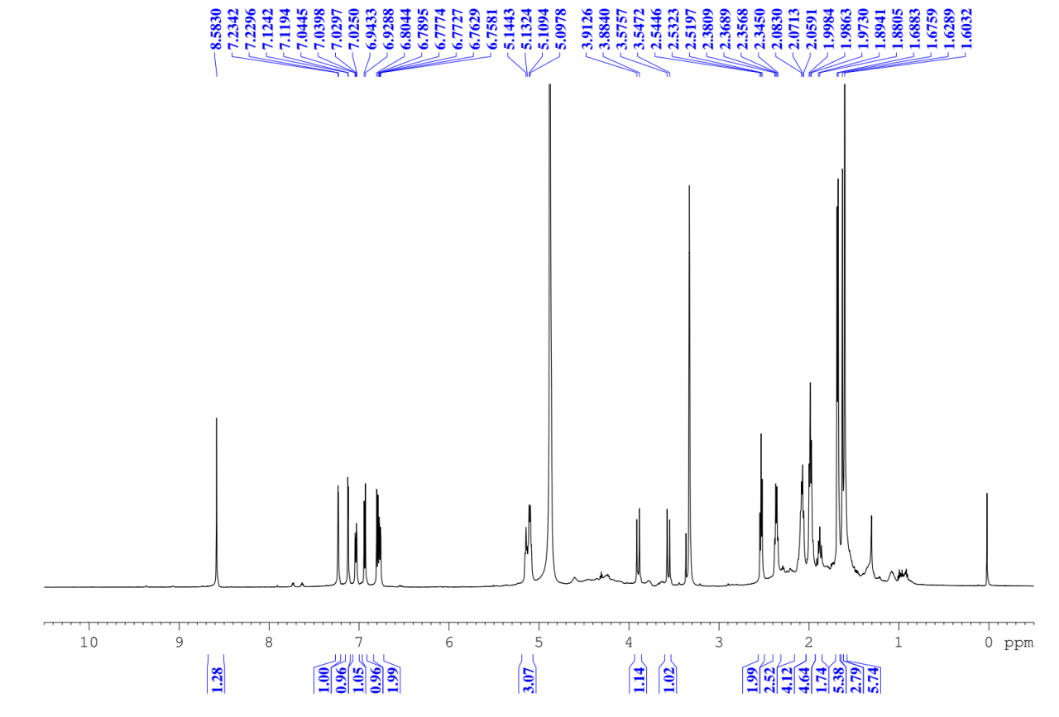


**Figure S7.** ^1^H NMR (600 MHz) spectrum of compound **1** in methanol-*d*_4_.


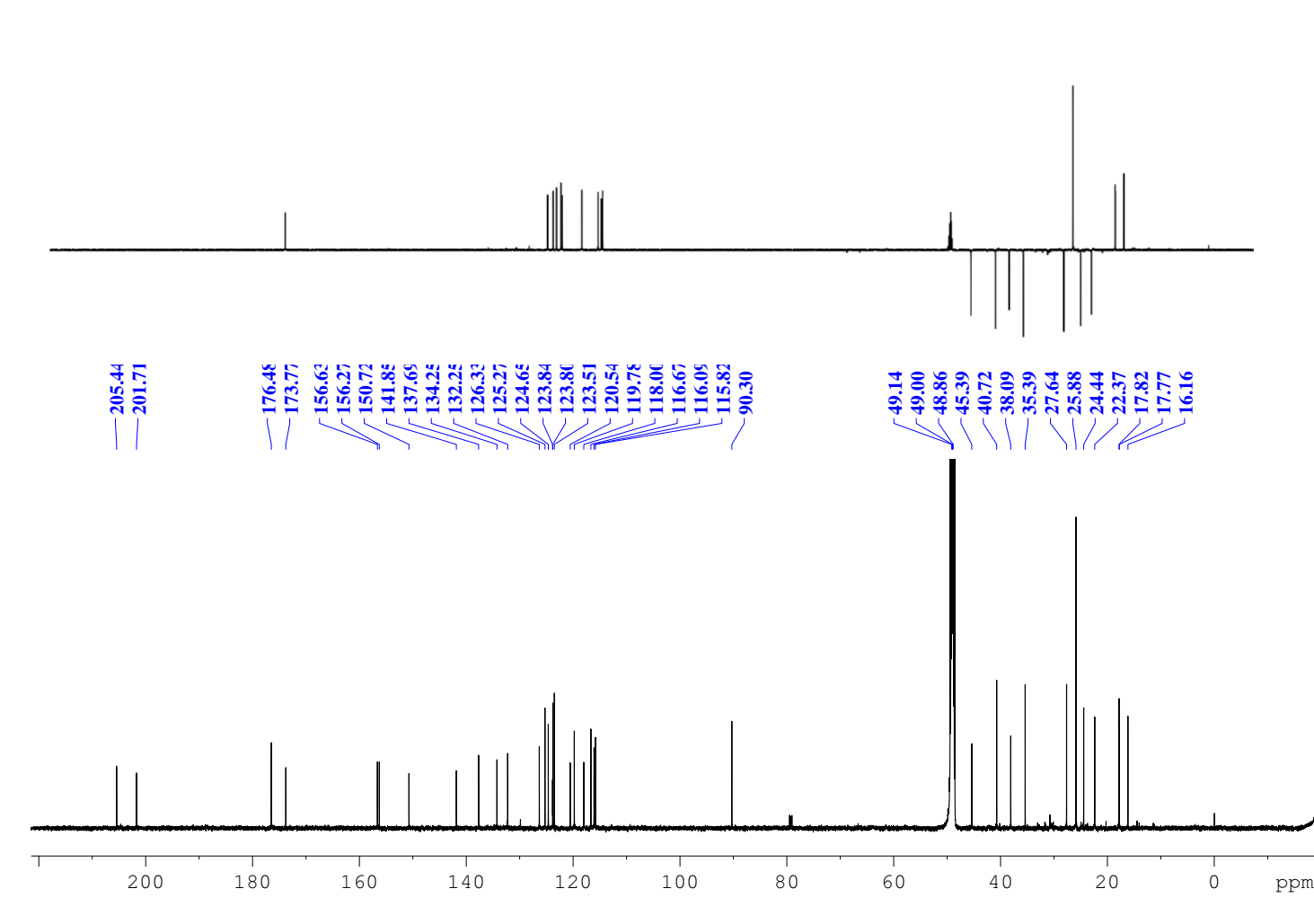


**Figure S8.** ^13^C NMR (150 MHz) and DEPT spectra of compound **1** in methanol-*d*_4_.


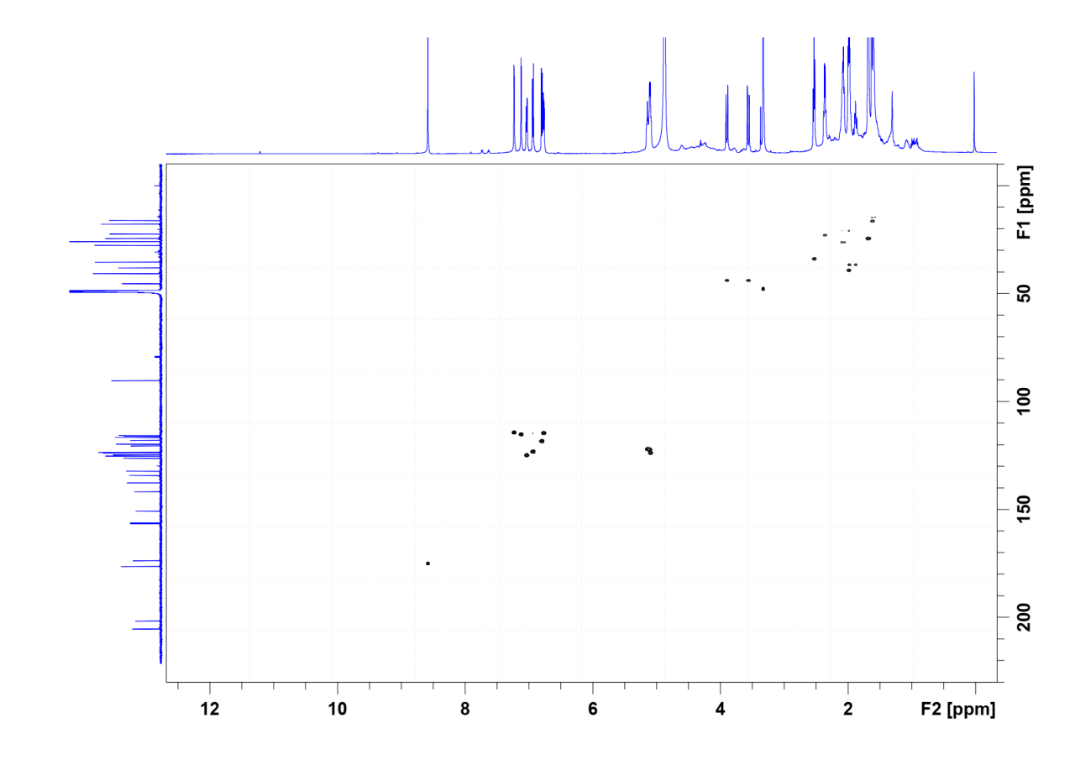


**Figure S9.** HSQC spectrum of compound **1** in methanol-*d*_4_.


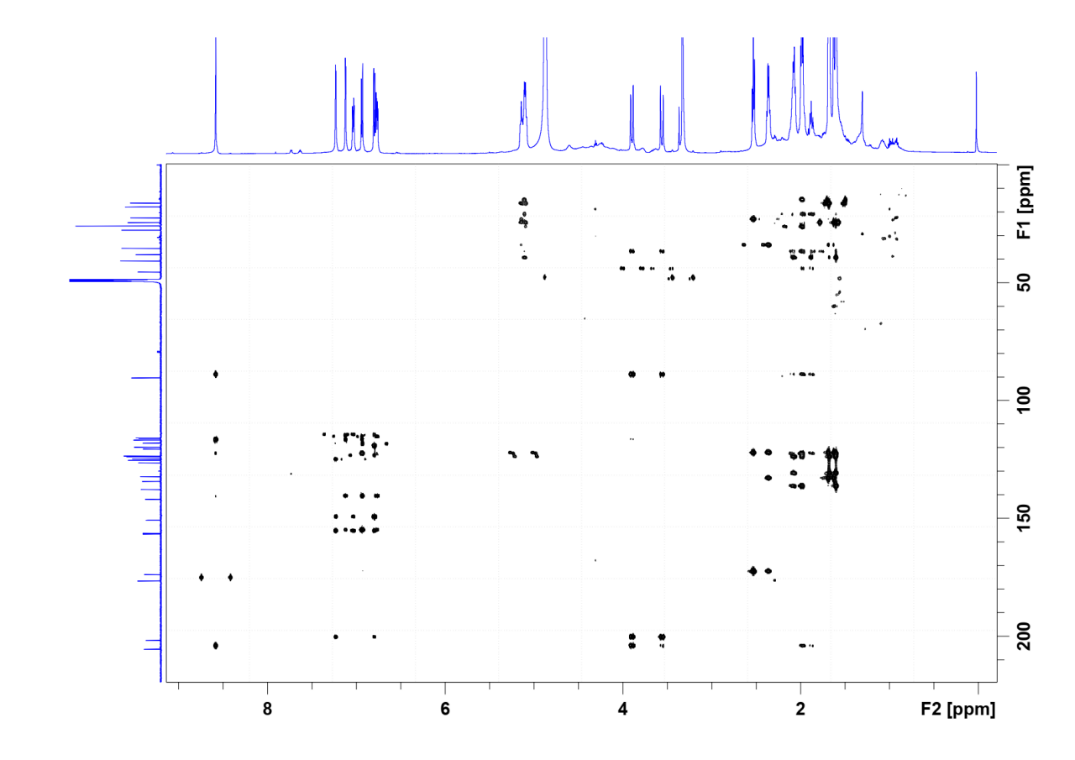


**Figure S10.** HMBC spectrum of compound **1** in methanol-*d*_4_.


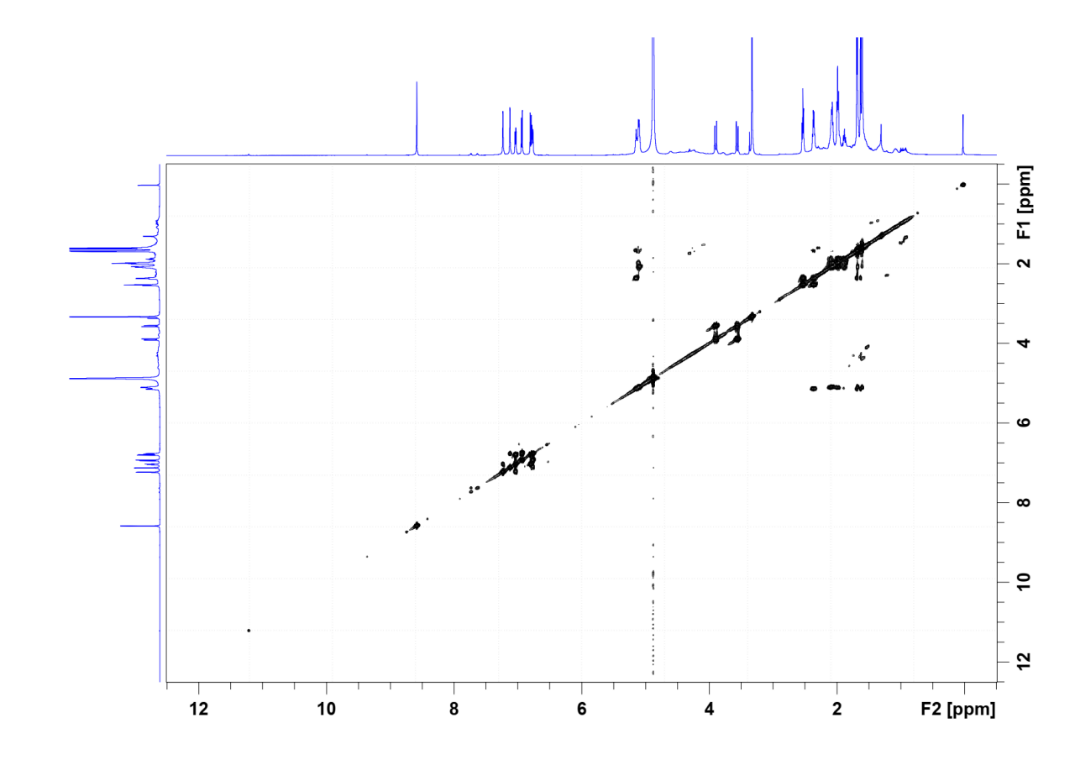


**Figure S11.** COSY spectrum of compound **1** in methanol-*d*_4_.


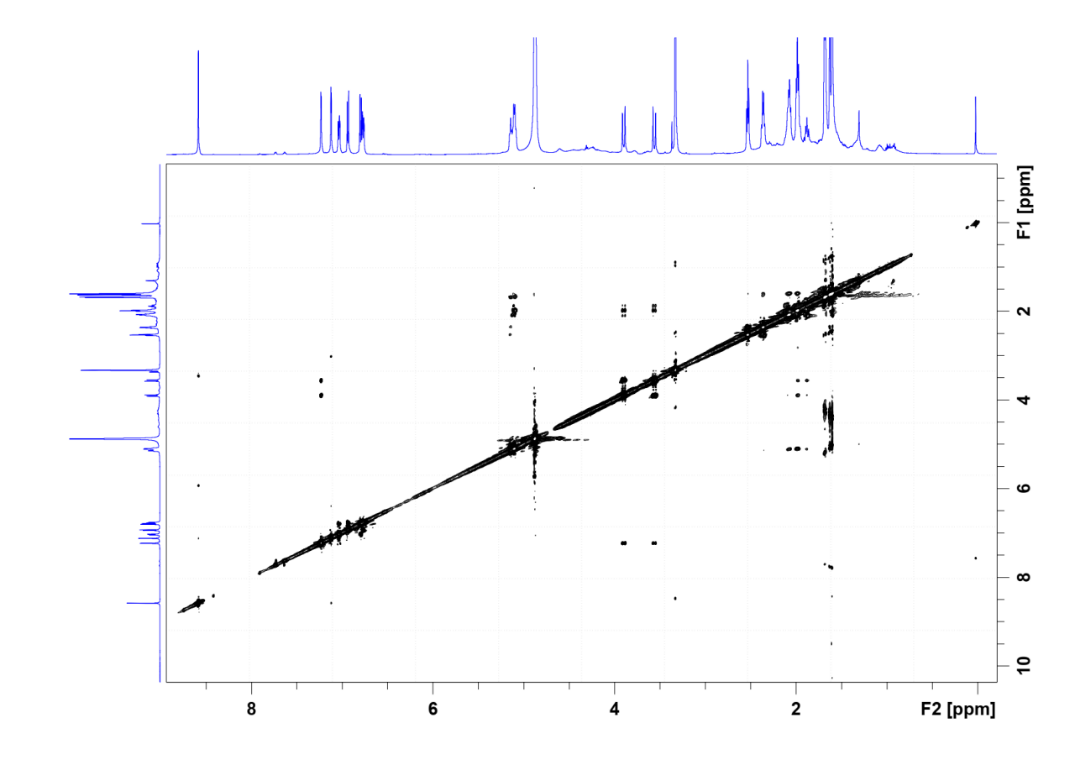


**Figure S12.** ROESY spectrum of compound **1** in methanol-*d*_4_.


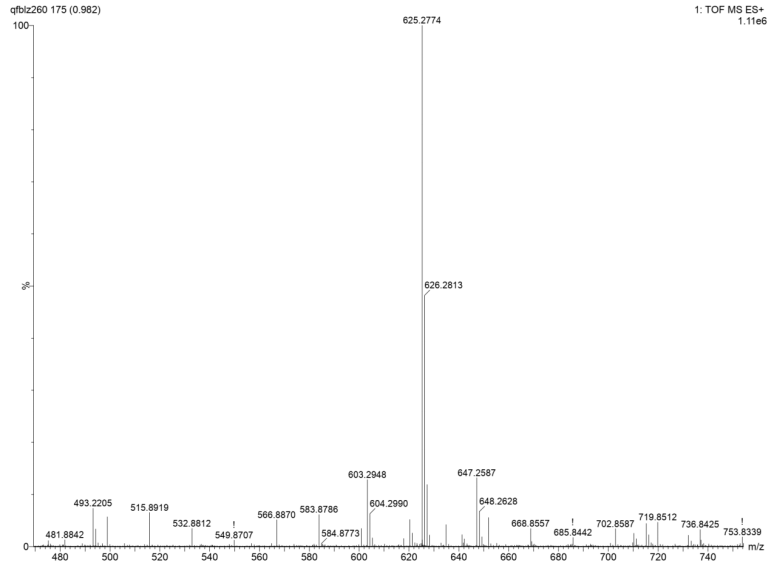


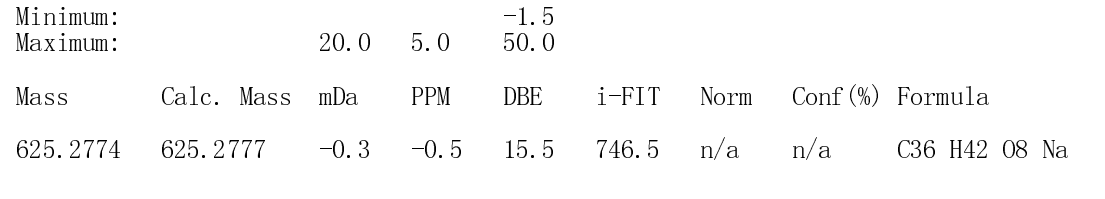


**Figure S13.** HRESIMS data of **1**.


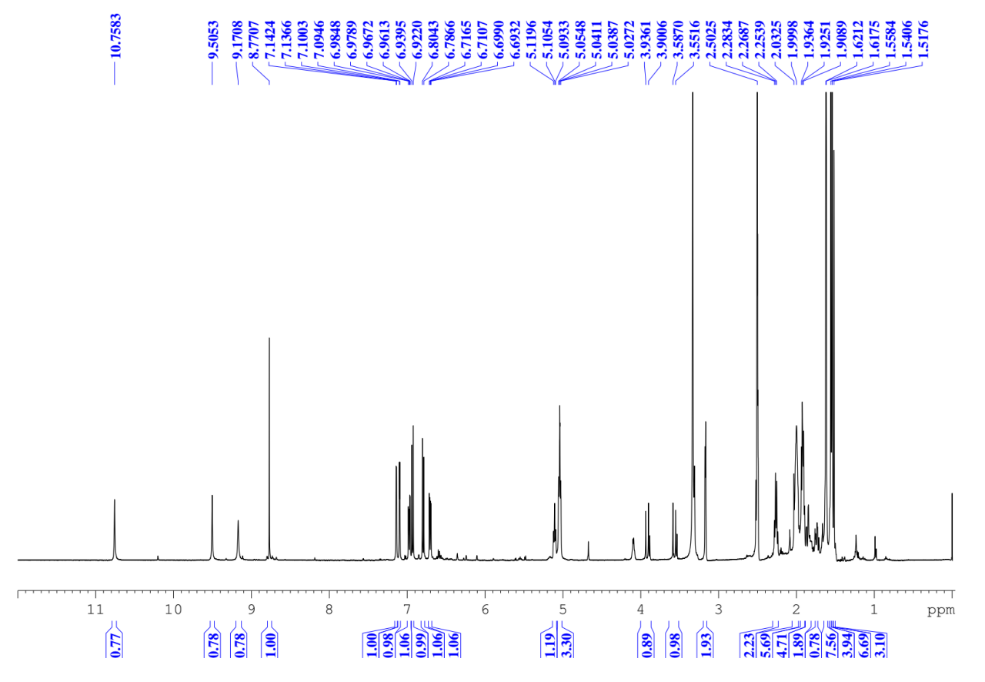


**Figure S14.** ^1^H NMR (500 MHz) spectrum of compound **2** in DMSO-*d*_6_.


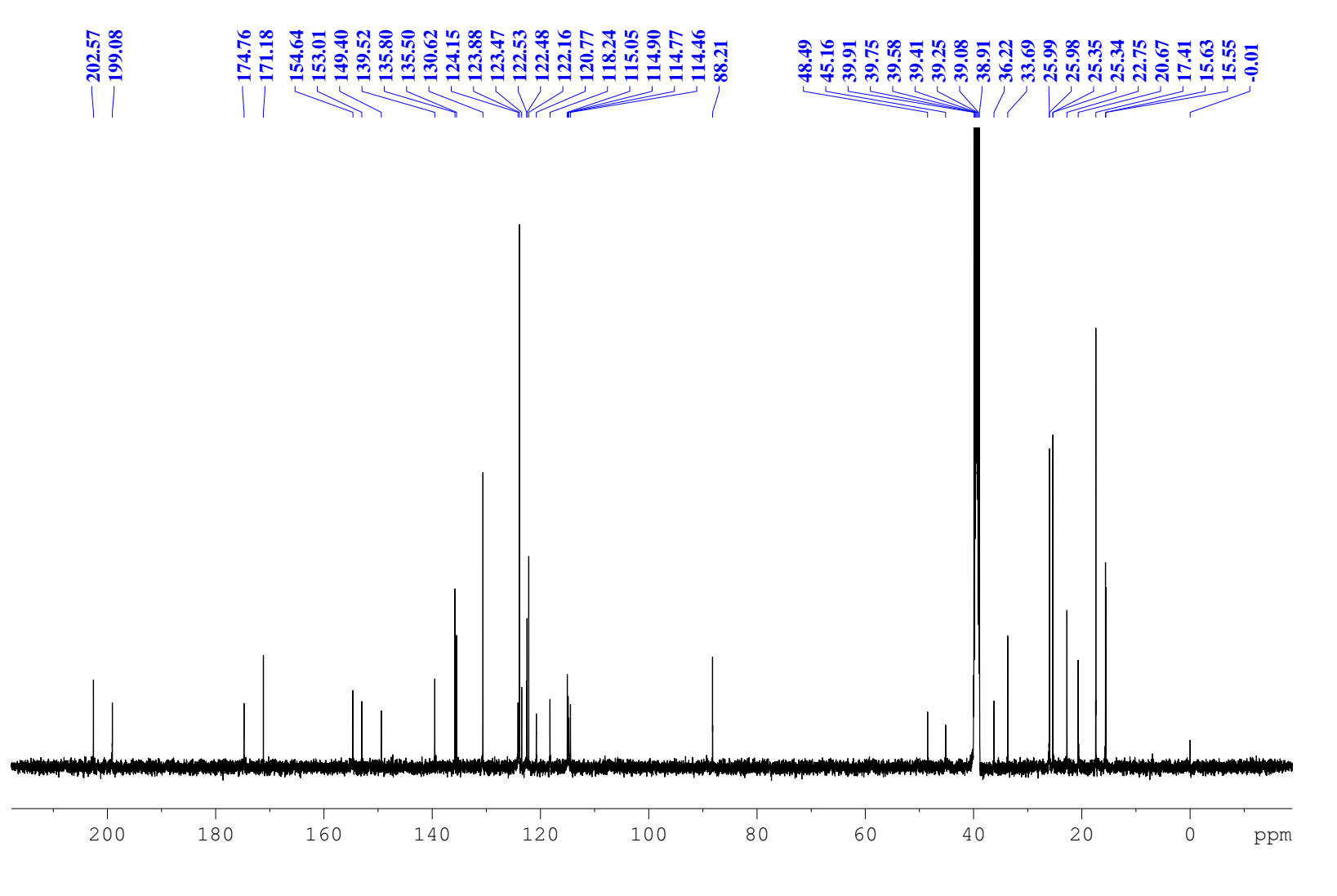


**Figure S15.** ^13^C NMR (125 MHz) spectrum of compound **2** in DMSO-*d*_6_.


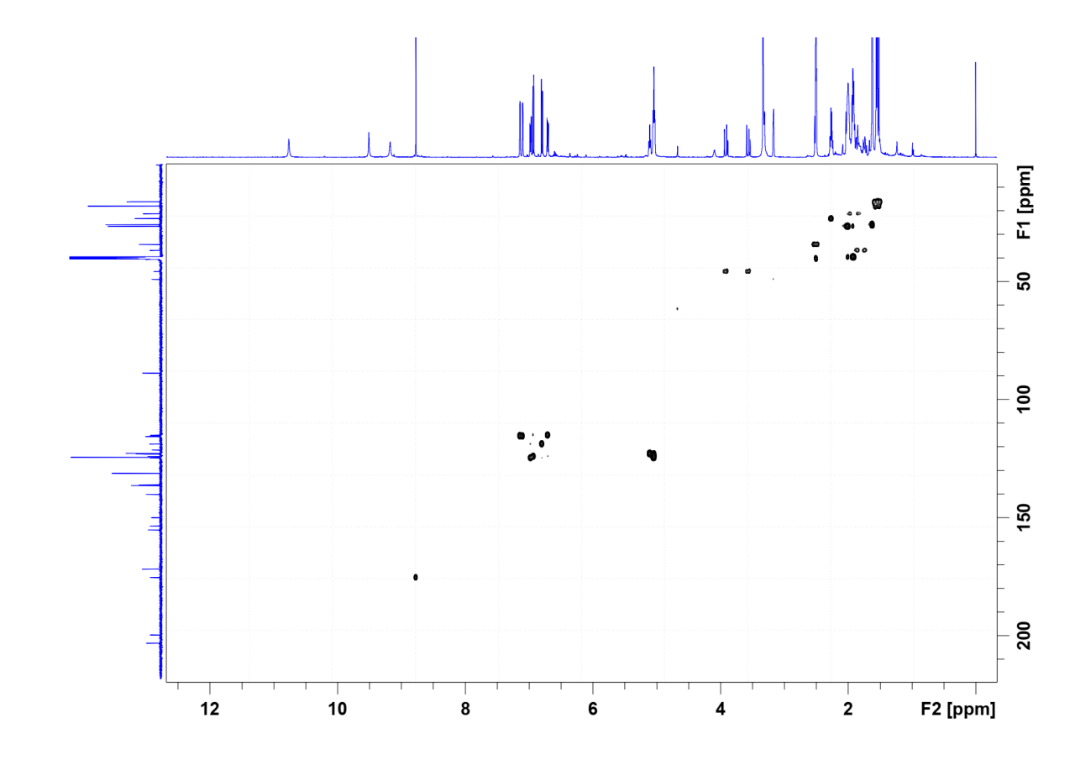


**Figure S16.** HSQC spectrum of compound **2** in DMSO-*d*_6_.


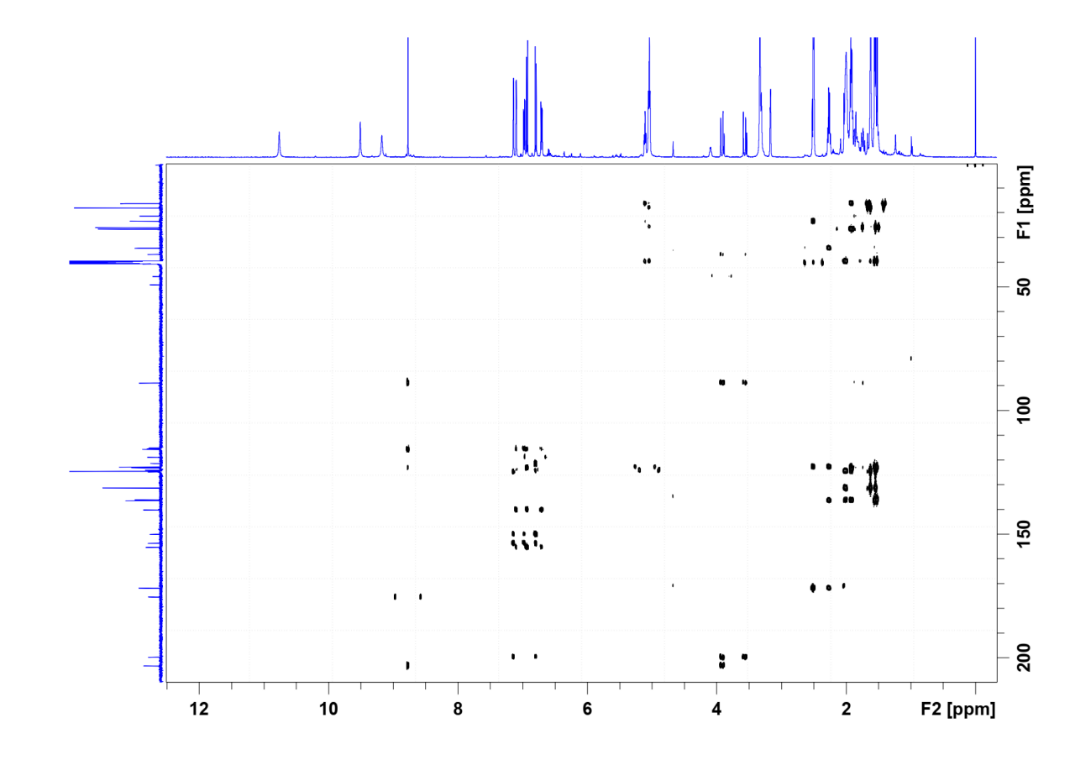


**Figure S17.** HMBC spectrum of compound **2** in DMSO-*d*_6_.


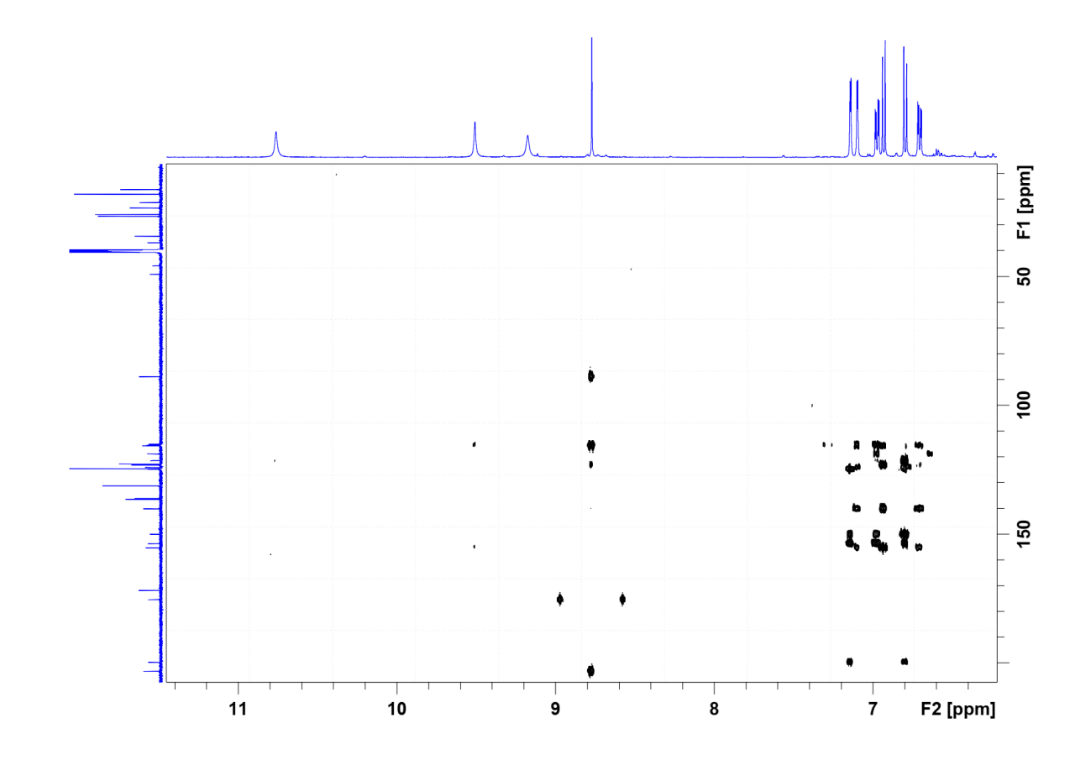


**Figure S18.** Enlarge HMBC spectrum of compound **2** in DMSO-*d*_6_.


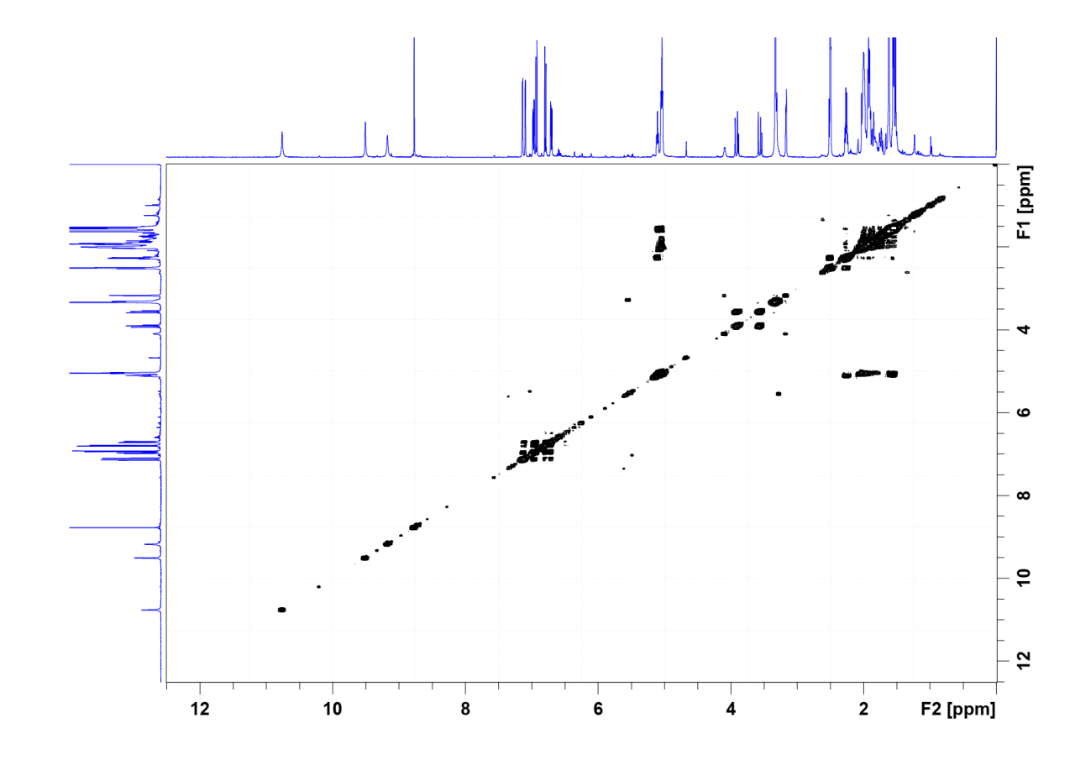


**Figure S19.** ^1^H-^1^H COSY spectrum of compound **2** in DMSO-*d*_6_.


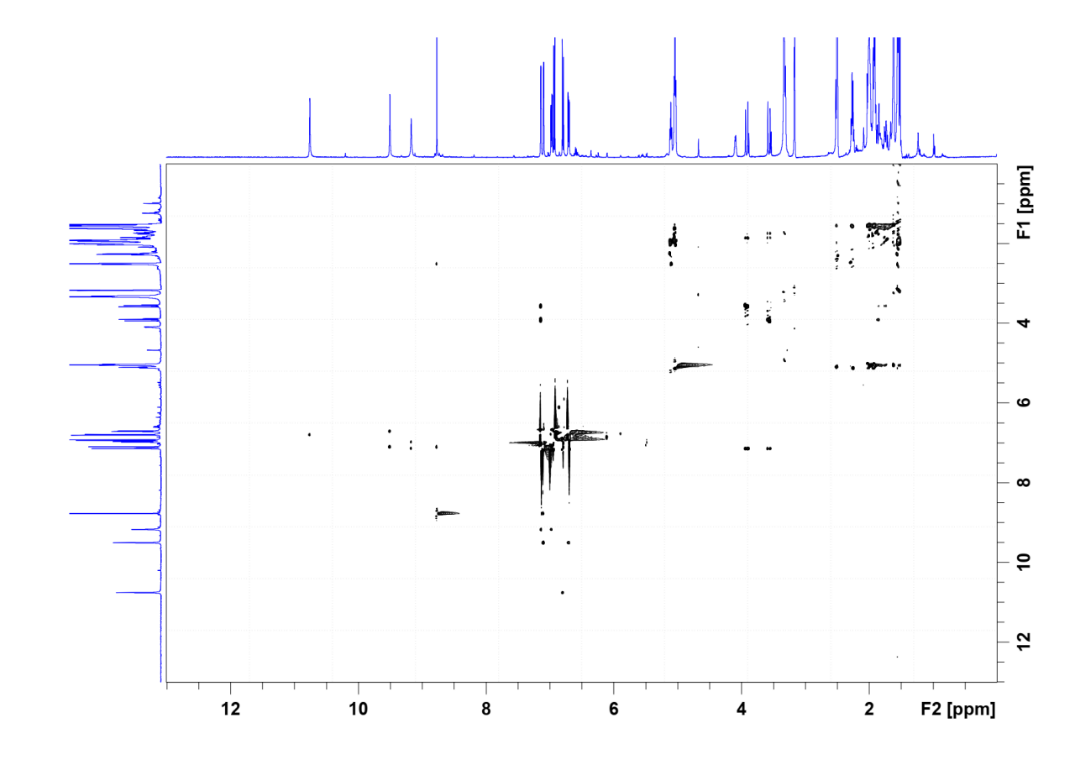


**Figure S20.** ROESY spectrum of compound **2** in DMSO-*d*_6_.


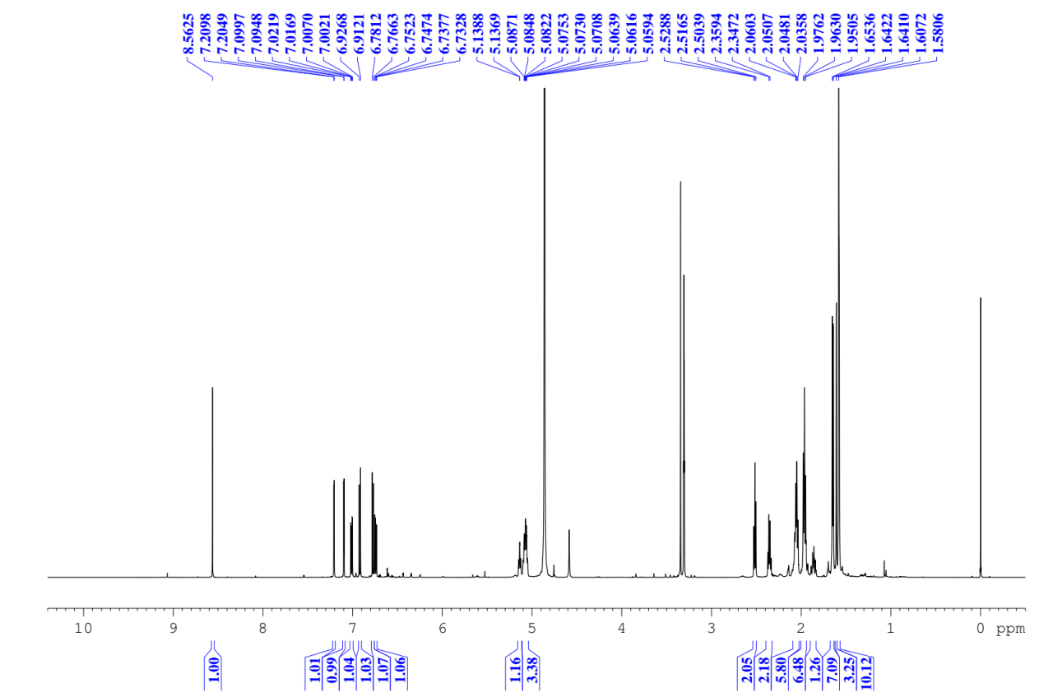


**Figure S21.** ^1^H NMR (600 MHz) spectrum of compound **2** in methanol-*d*_4_.


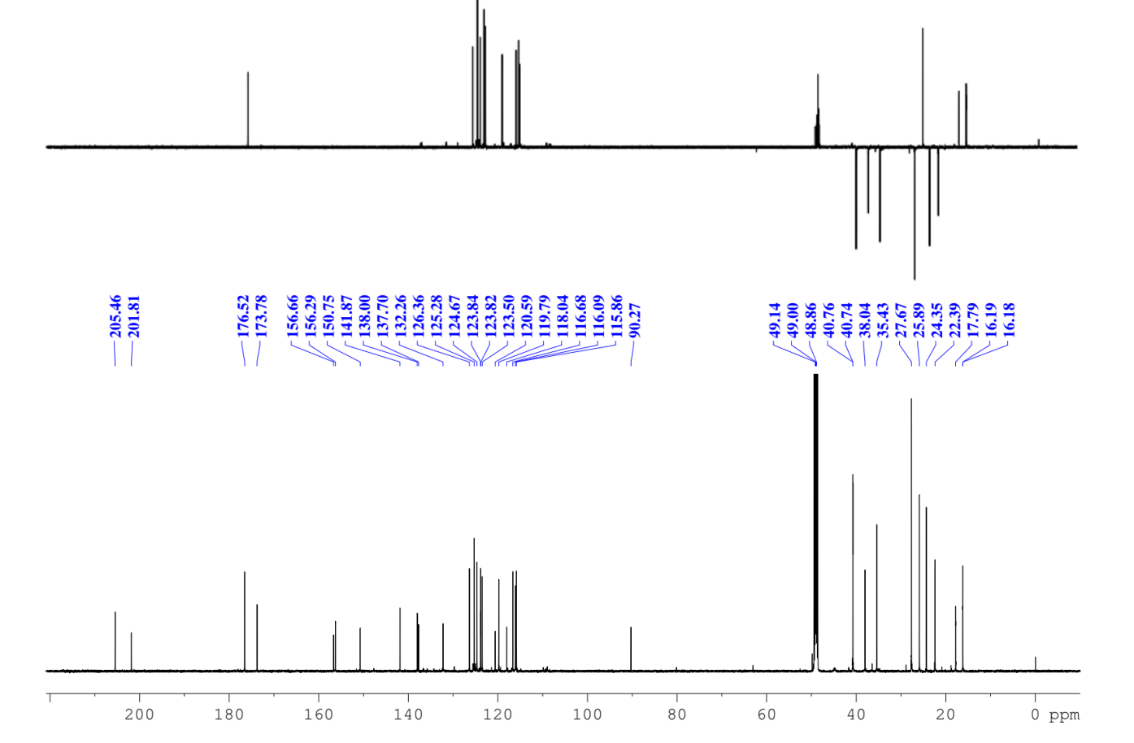


**Figure S22.** ^13^C NMR (150 MHz) and DEPT spectra of compound **2** in methanol-*d*_4_.


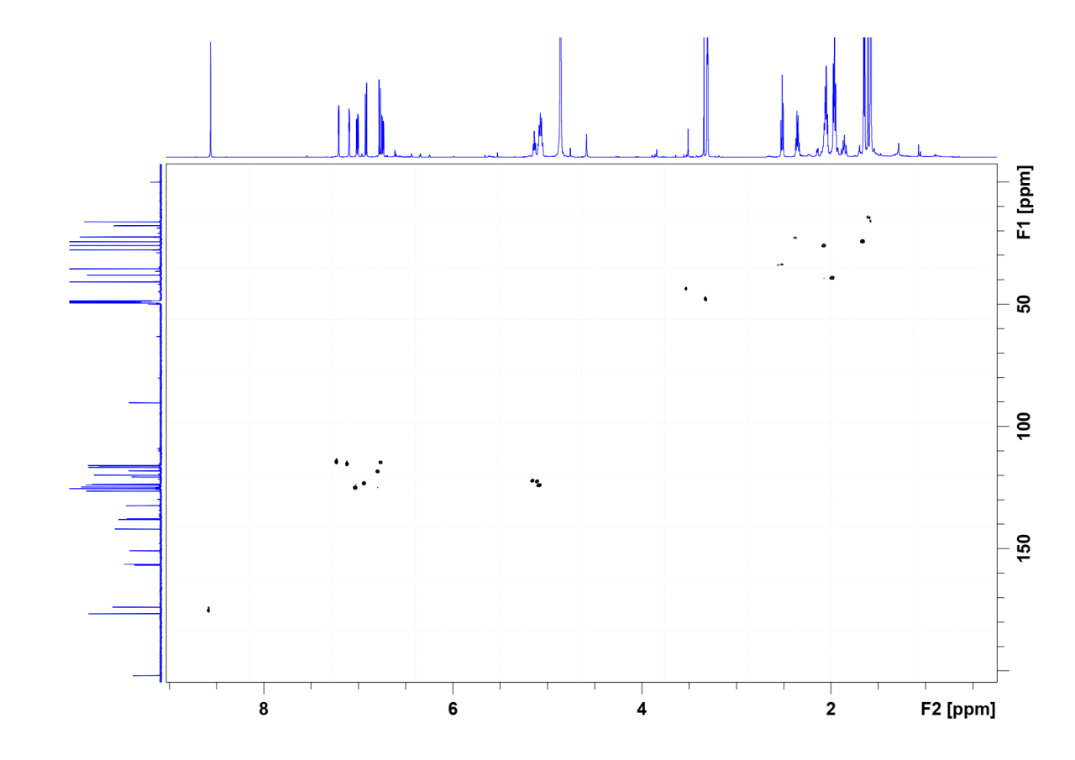


**Figure S23.** HSQC spectrum of compound **2** in methanol-*d*_4_.


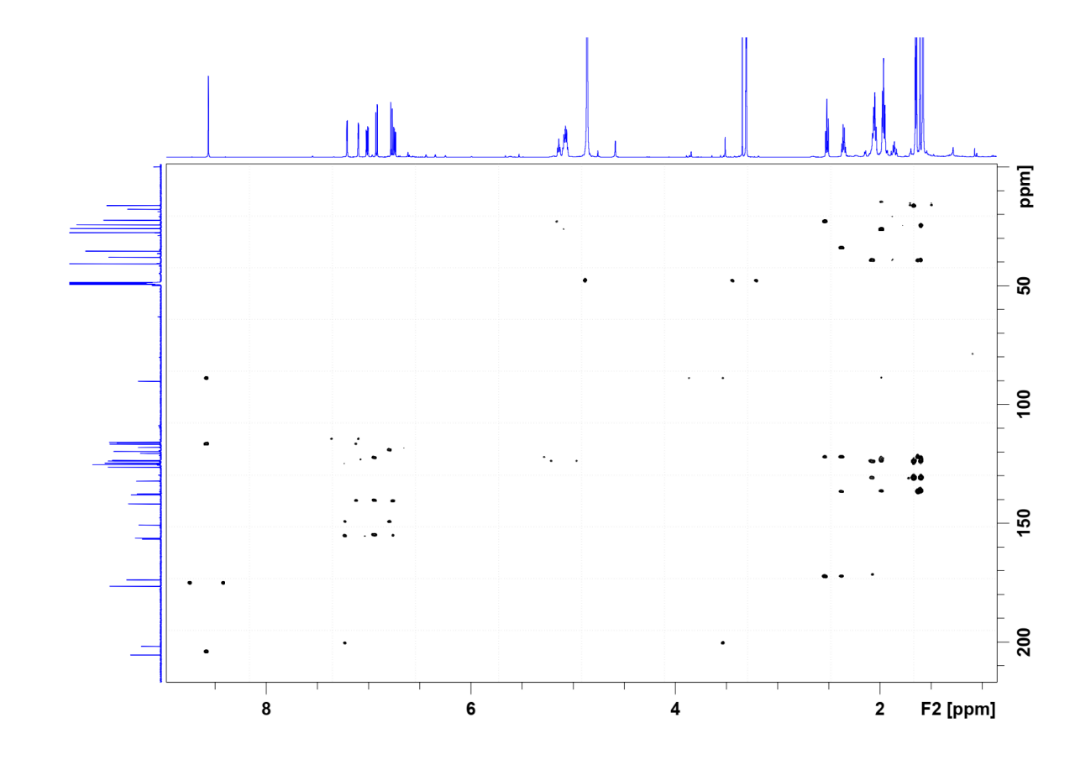


**Figure S24.** HMBC spectrum of compound **2** in methanol-*d*_4_.


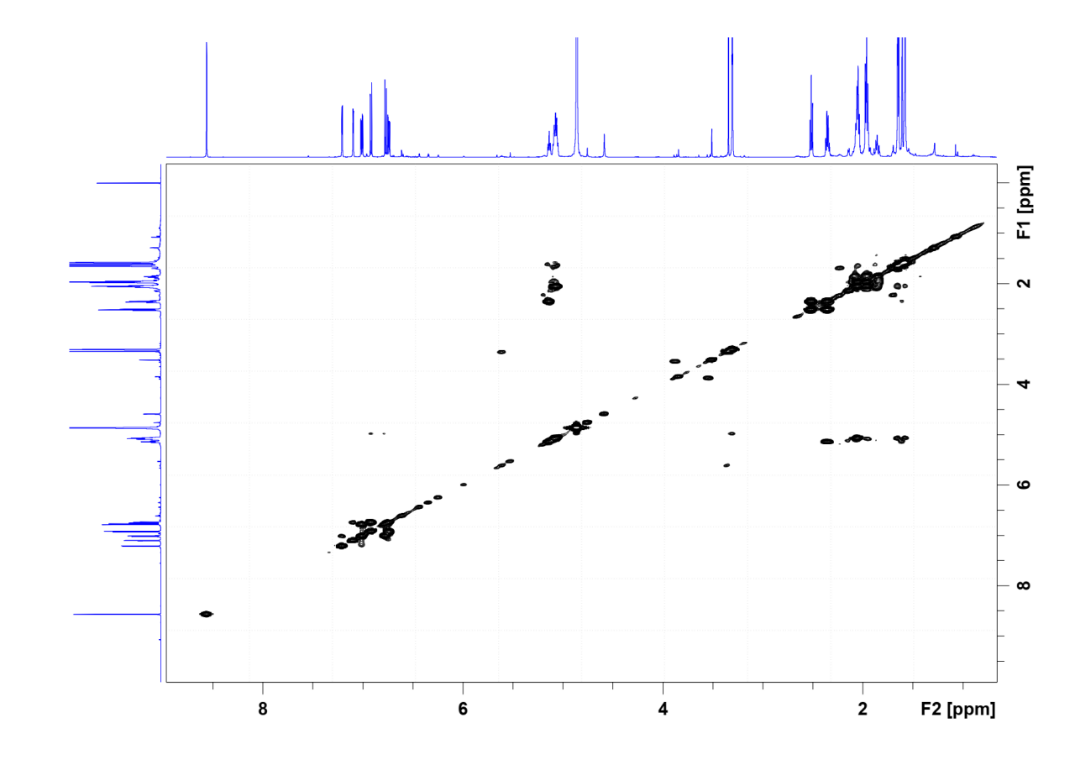


**Figure S25.** COSY spectrum of compound **2** in methanol-*d*_4_.


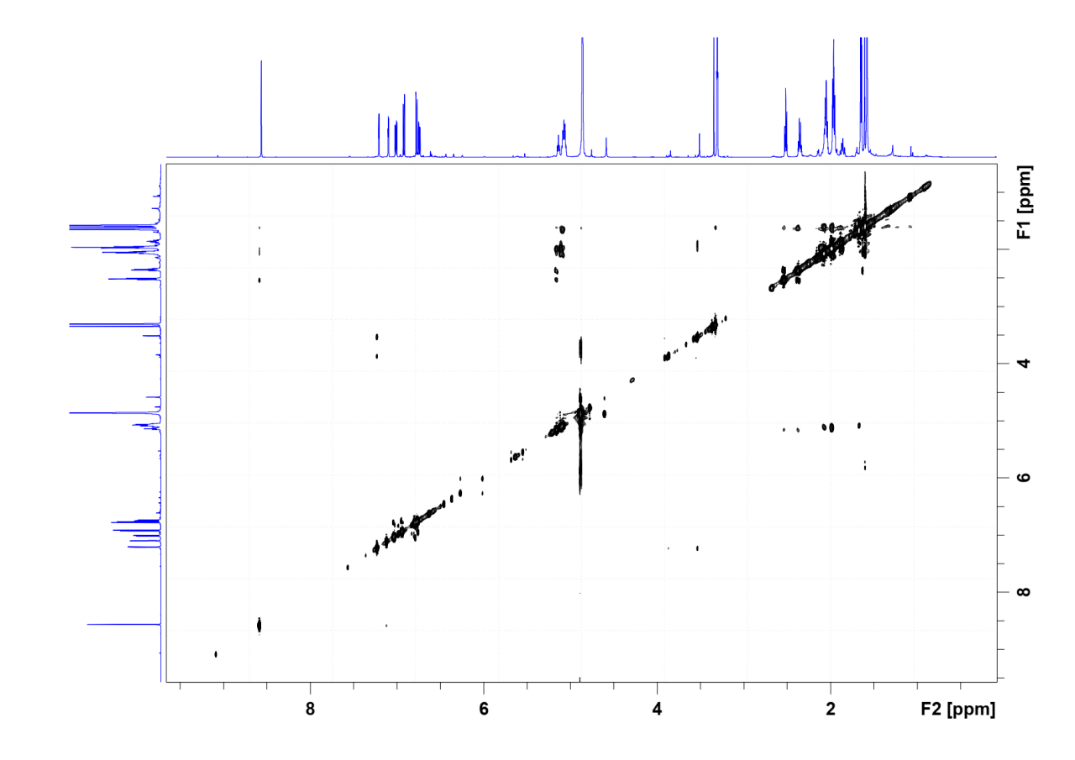


**Figure S26.** ROESY spectrum of compound **2** in methanol-*d*_4_.


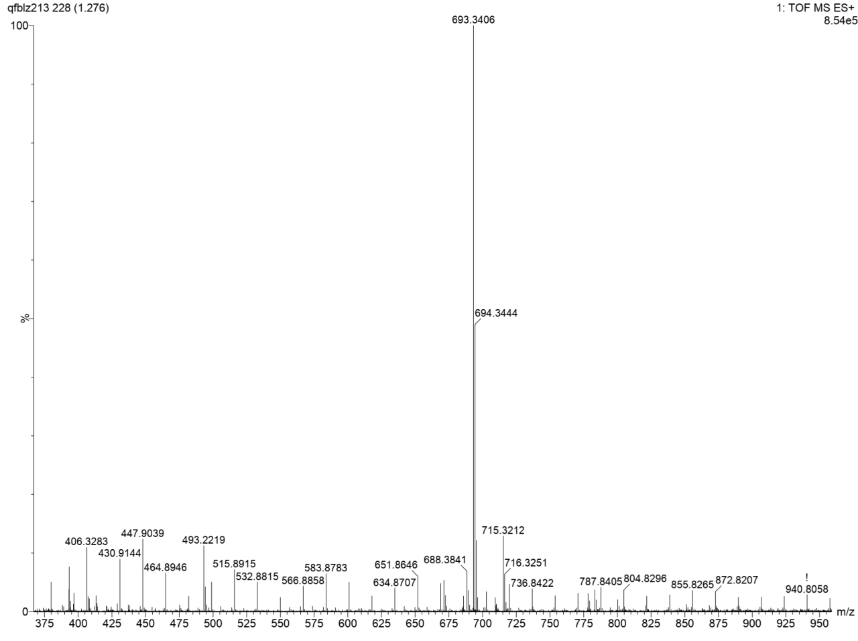


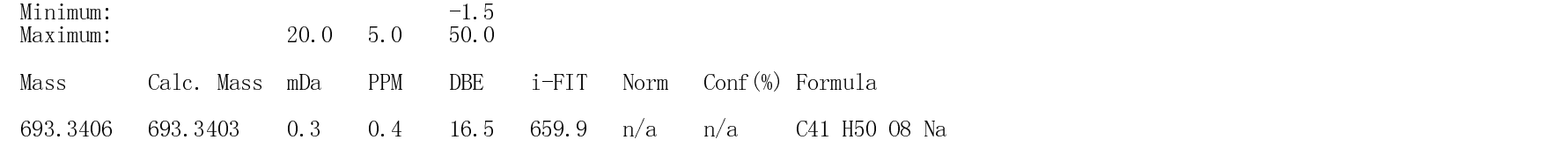


**Figure S27.** HRESIMS of **2**.


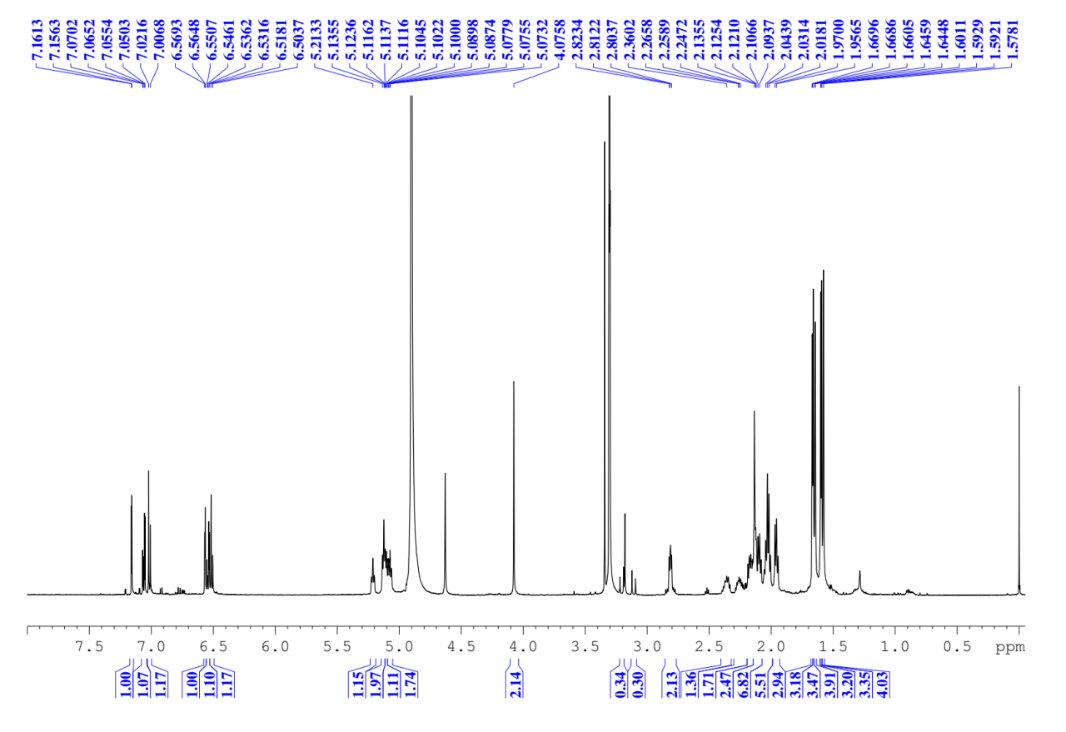


**Figure S28.** ^1^H NMR (600 MHz) spectrum of compound **3** in methanol-*d*_4_.


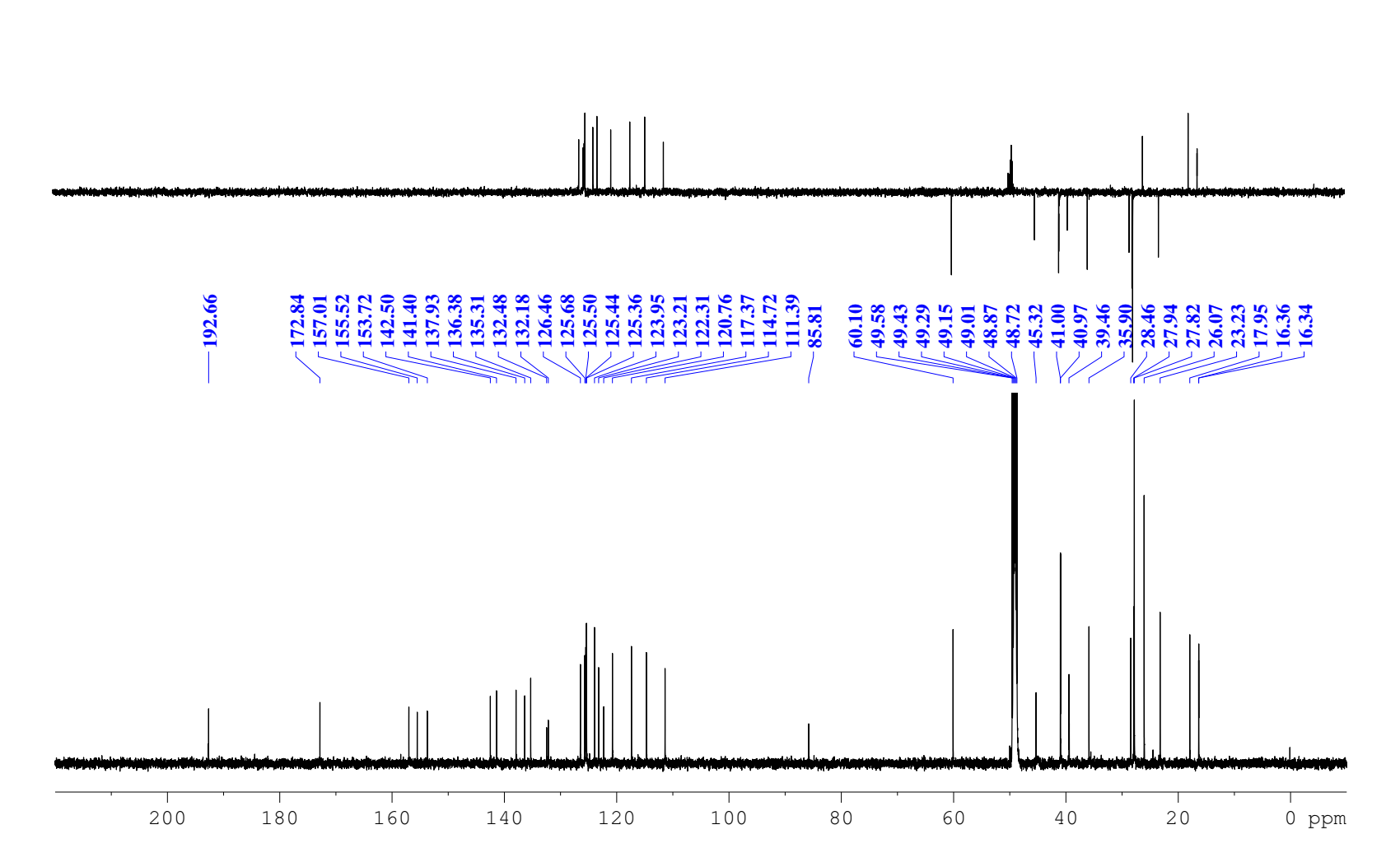


**Figure S29.** ^13^C NMR (150 MHz) and DEPT spectra of compound **3** in methanol-*d*_4_.


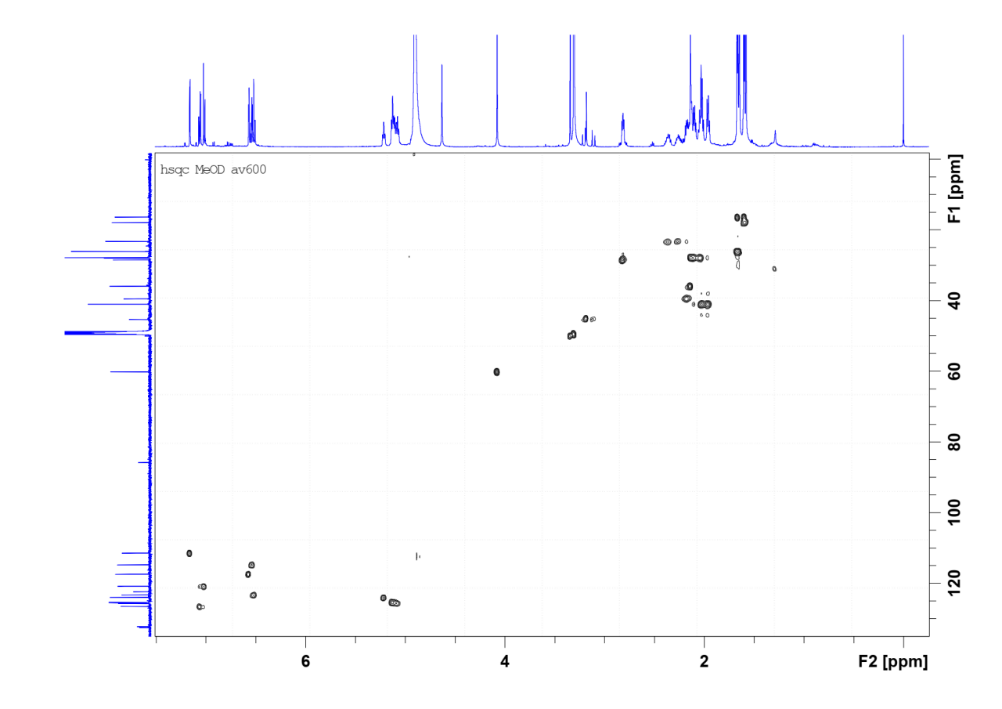


**Figure S30.** HSQC spectrum of compound **3** in methanol-*d*_4_.


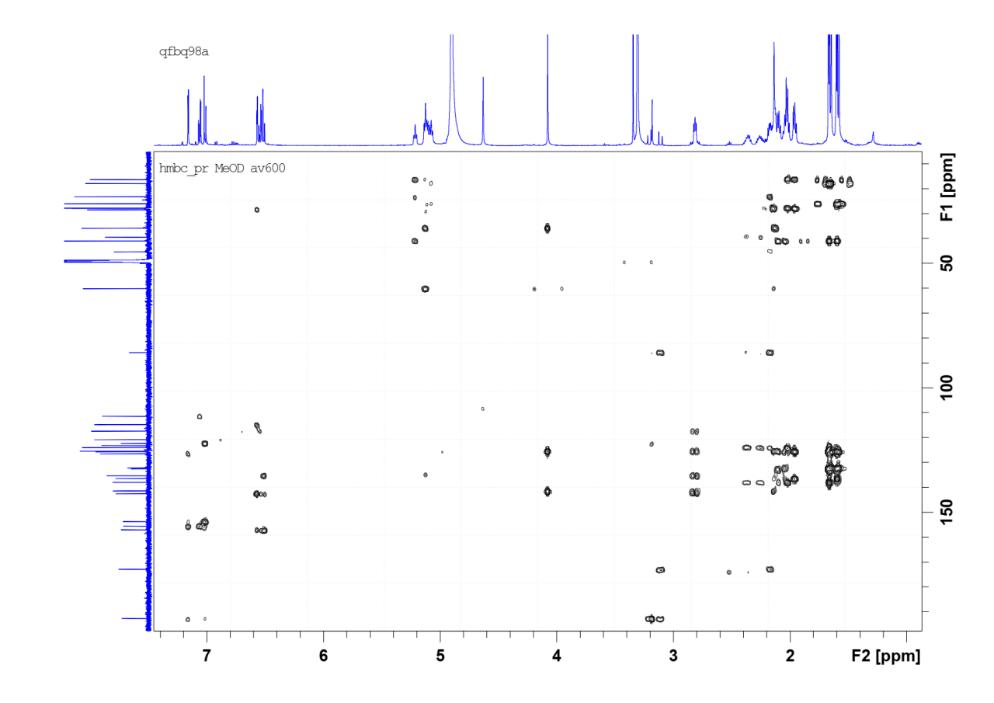


**Figure S31.** HMBC spectrum of compound **3** in methanol-*d*_4_.


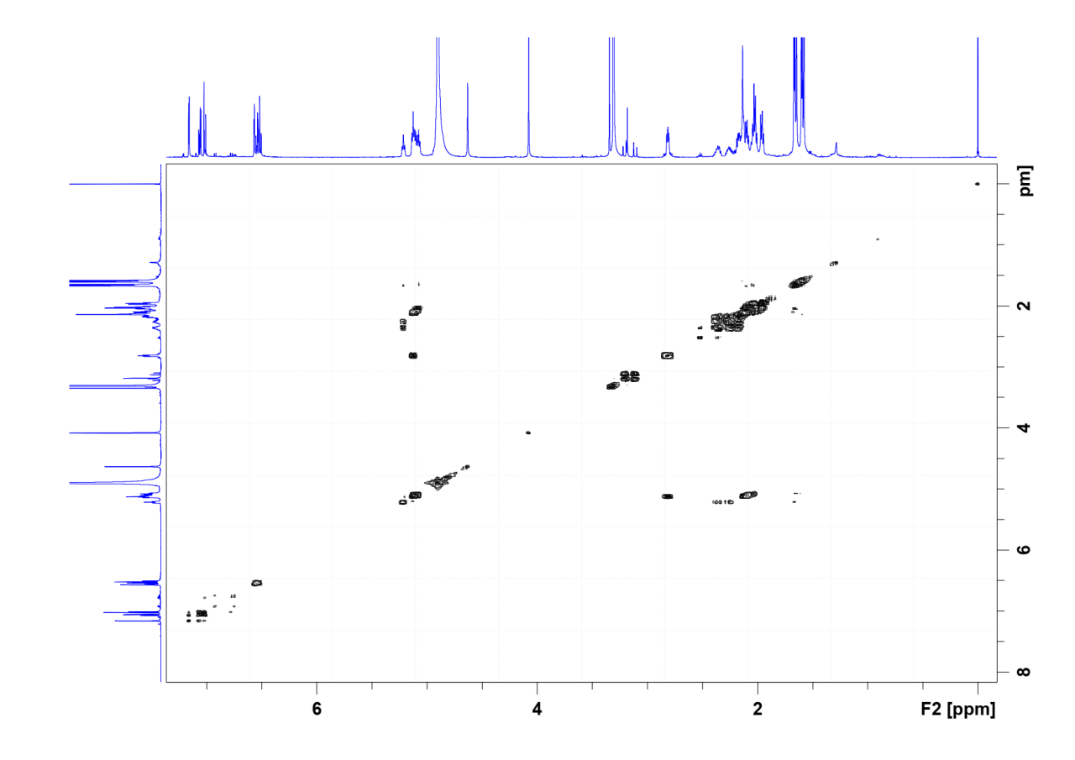


**Figure S32.** ^1^H-^1^H COSY spectrum of compound **3** in methanol-*d*_4_.


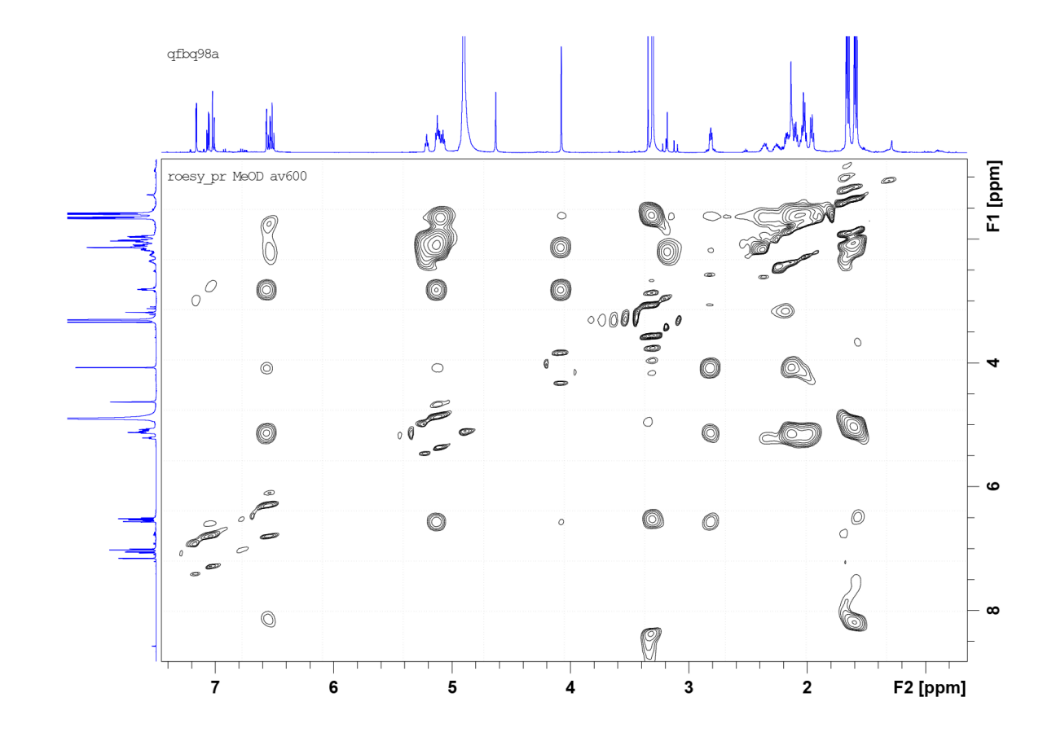


**Figure S33.** ROESY spectrum of compound **3** in methanol-*d*_4_.


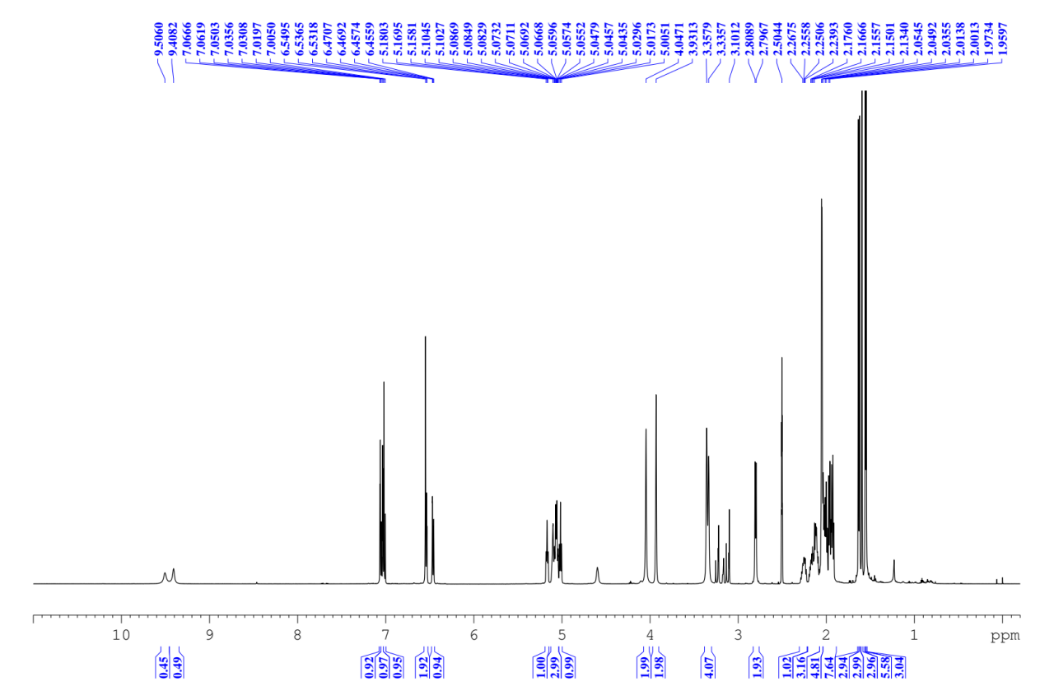


**Figure S34.** ^1^H NMR (600 MHz) spectrum of compound **3** in DMSO-*d*_6_.


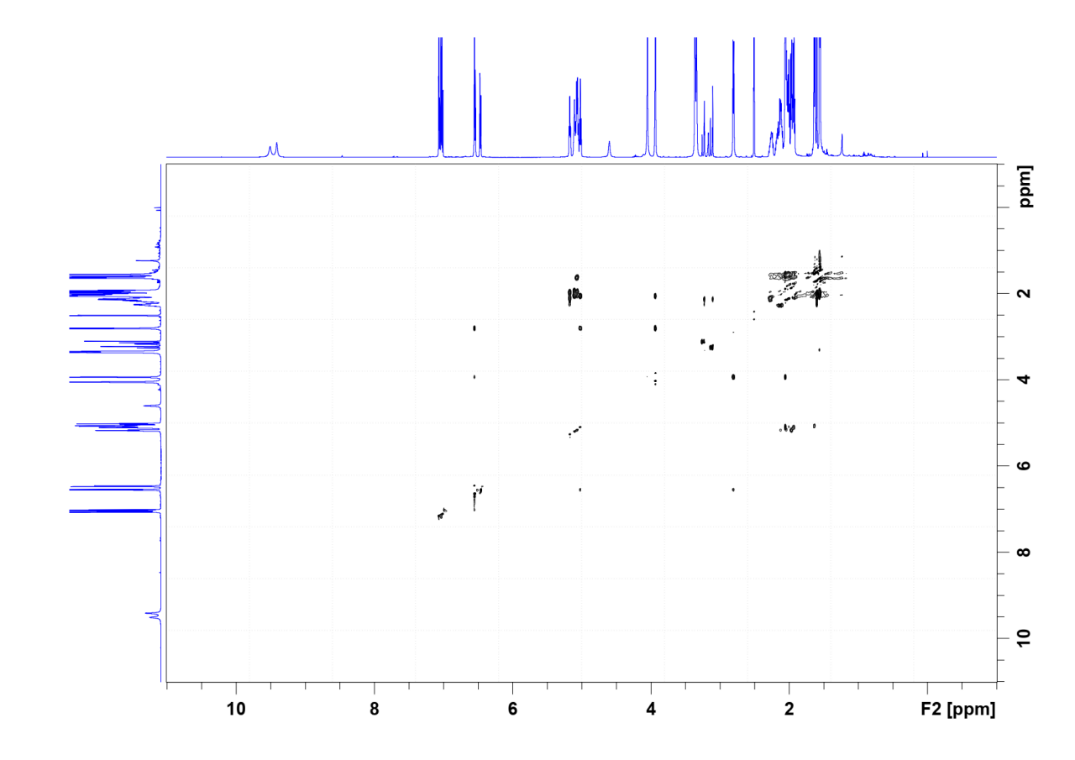


**Figure S35.** ROESY spectrum of compound **3** in DMSO-*d*_6_.


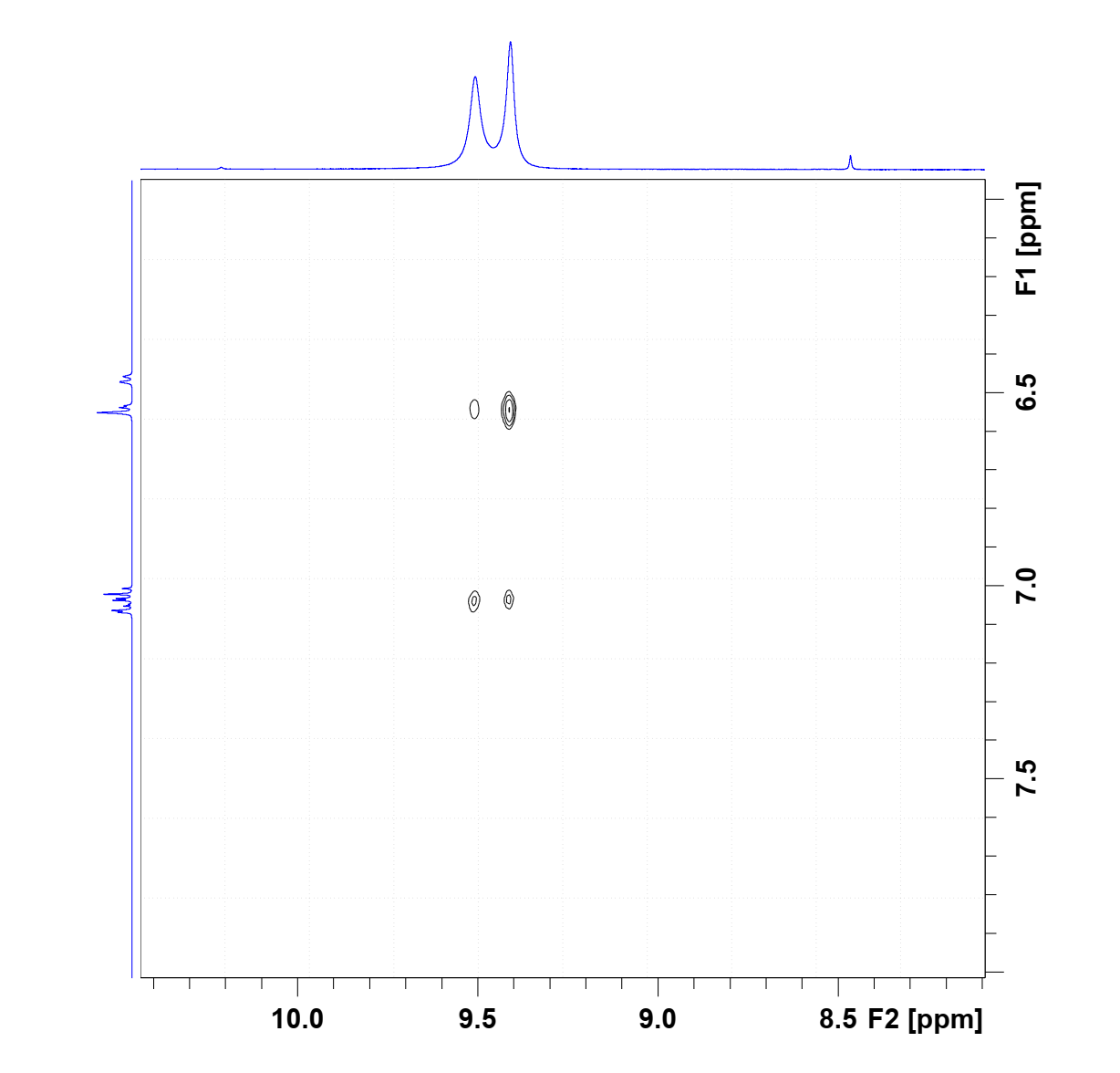


**Figure S36.** Enlarge ROESY spectrum of compound **3** in DMSO-*d*_6_.

[M-H]^-^ m/z 669.3777

| Hit | Formula | m/z | RDB | ppm |
| --- | --- | --- | --- | --- |
| 1 | C_42_H_53_O_7_ | 669.3797 | 16.0 | -3.0 |

**Figure S37.** HRESIMS of **3**.


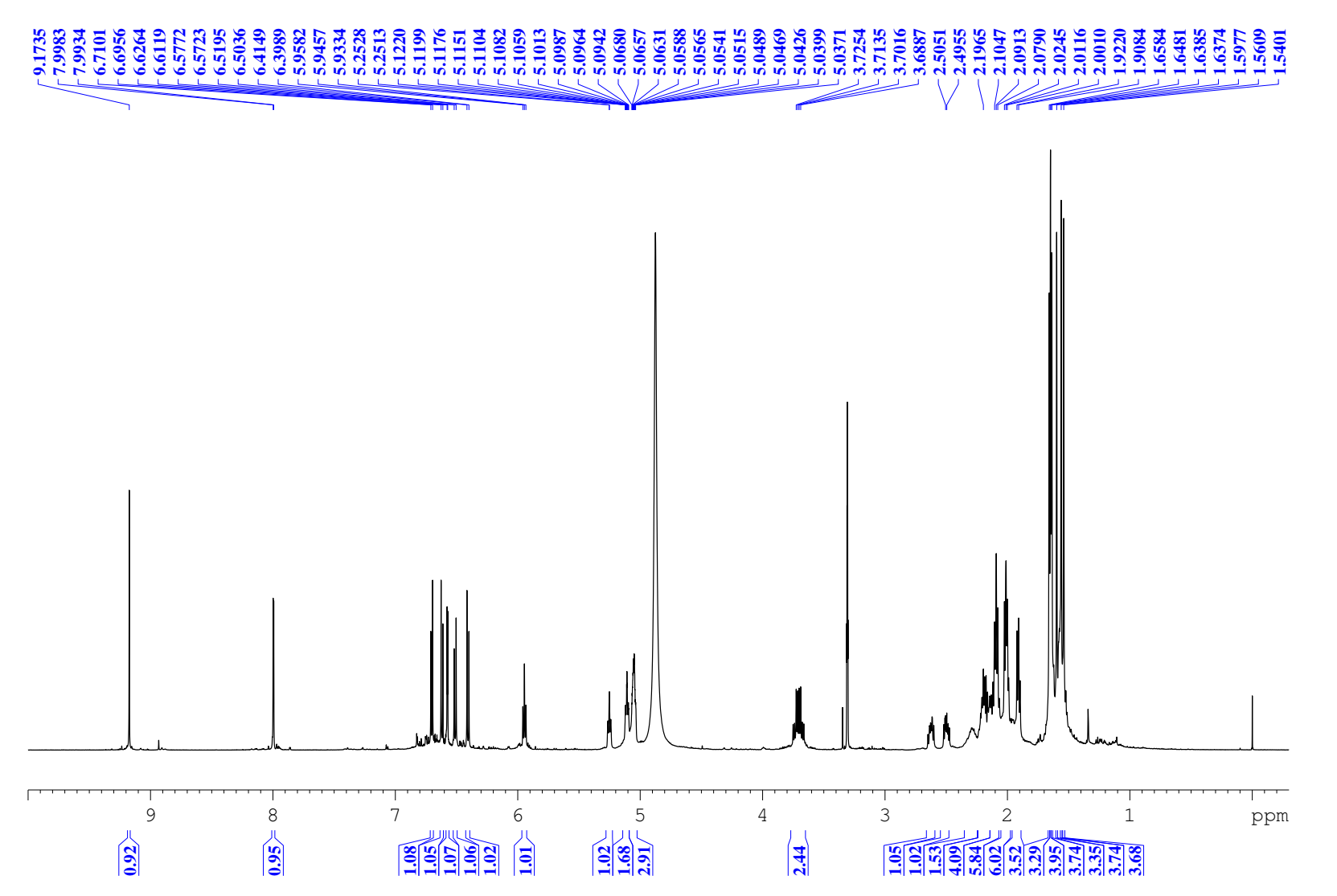


**Figure S38.** ^1^H NMR (600 MHz) spectrum of compound **4** in methanol-*d*_4_.


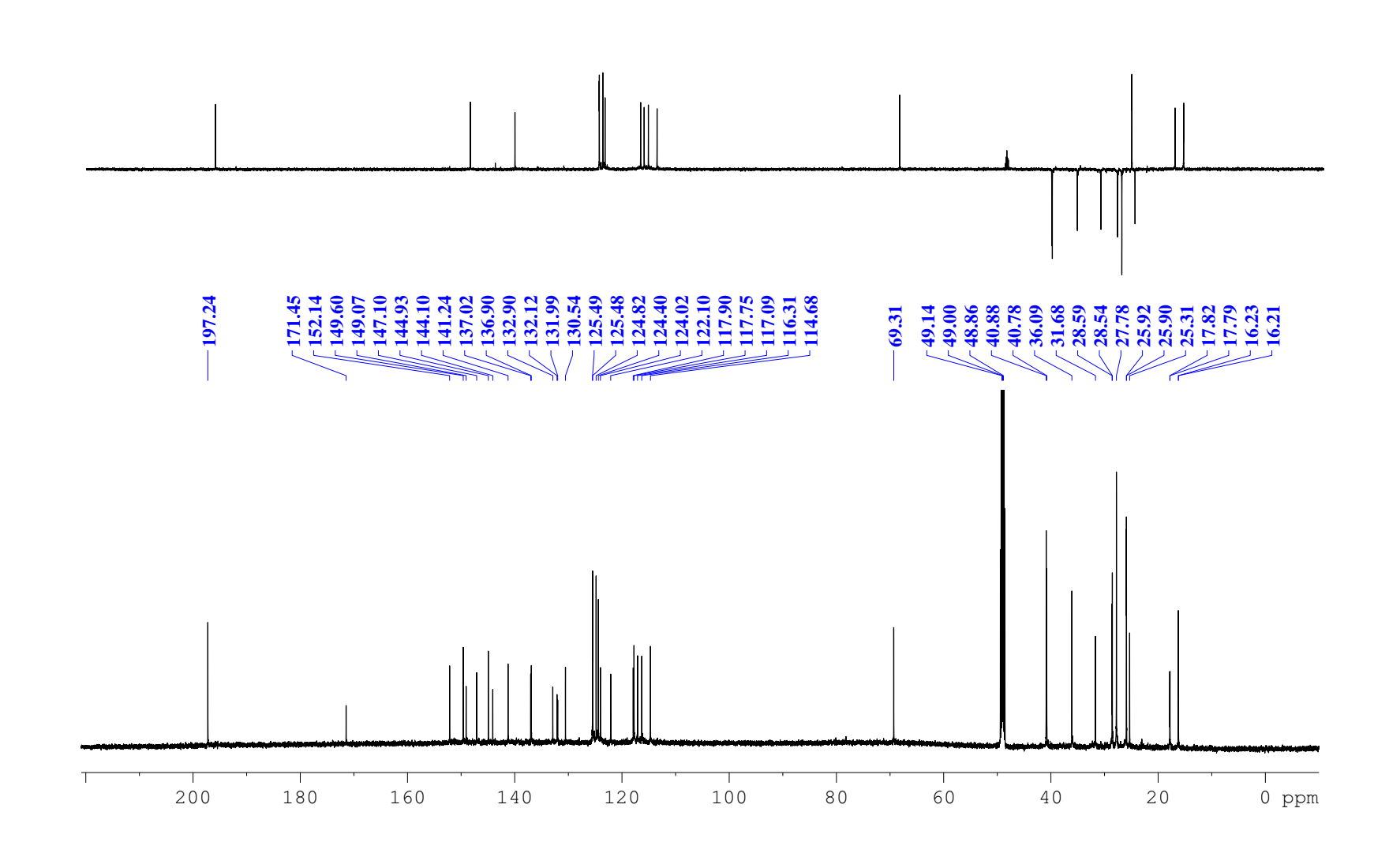


**Figure S39.** ^13^C NMR (150 MHz) spectrum of compound **4** in methanol-*d*_4_.


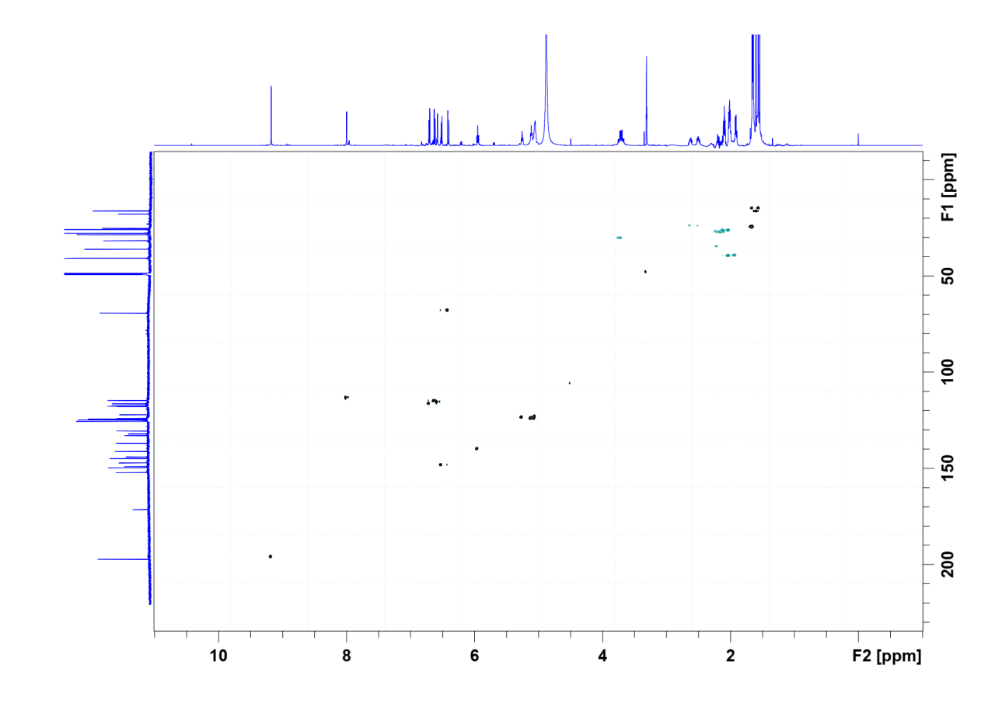


**Figure S40.** HSQC spectrum of compound **4** in methanol-*d*_4_.


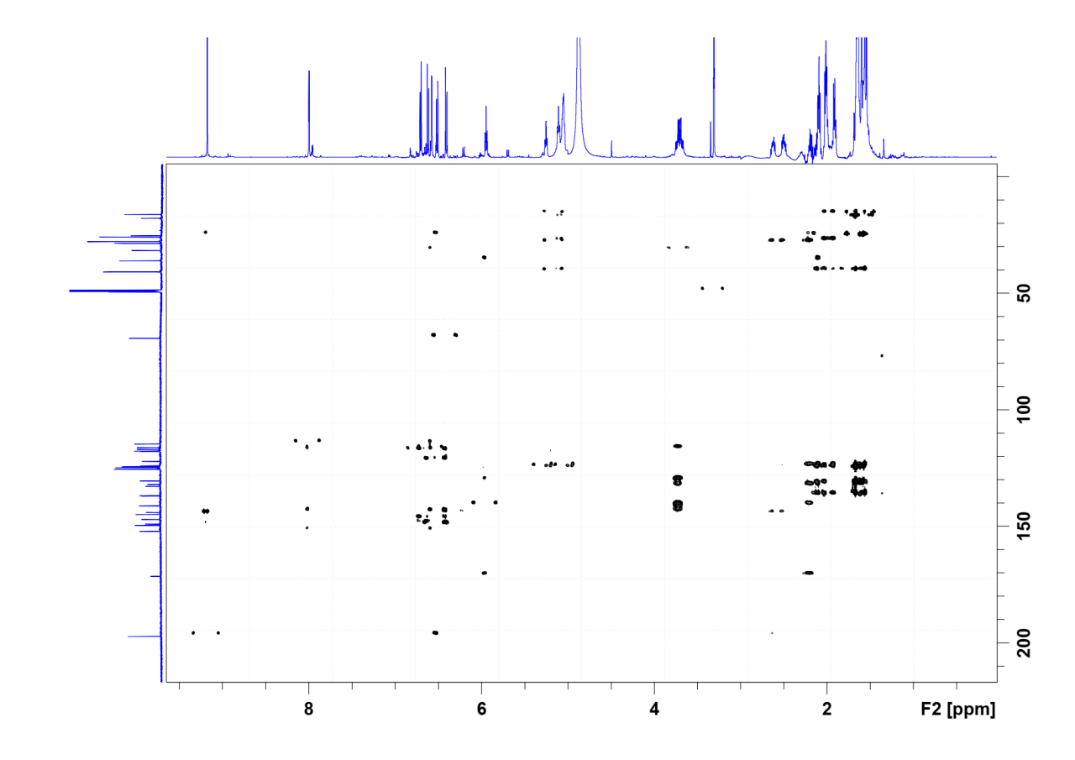


**Figure S41.** HMBC spectrum of compound **4** in methanol-*d*_4_.


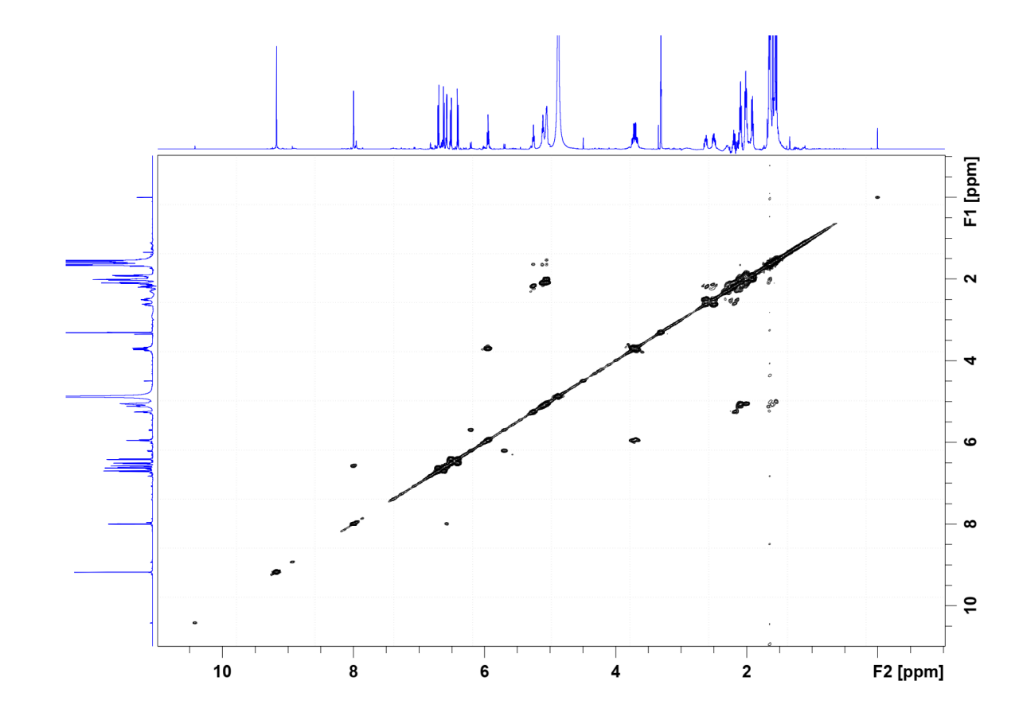


**Figure S42.** COSY spectrum of compound **4** in methanol-*d*_4_.


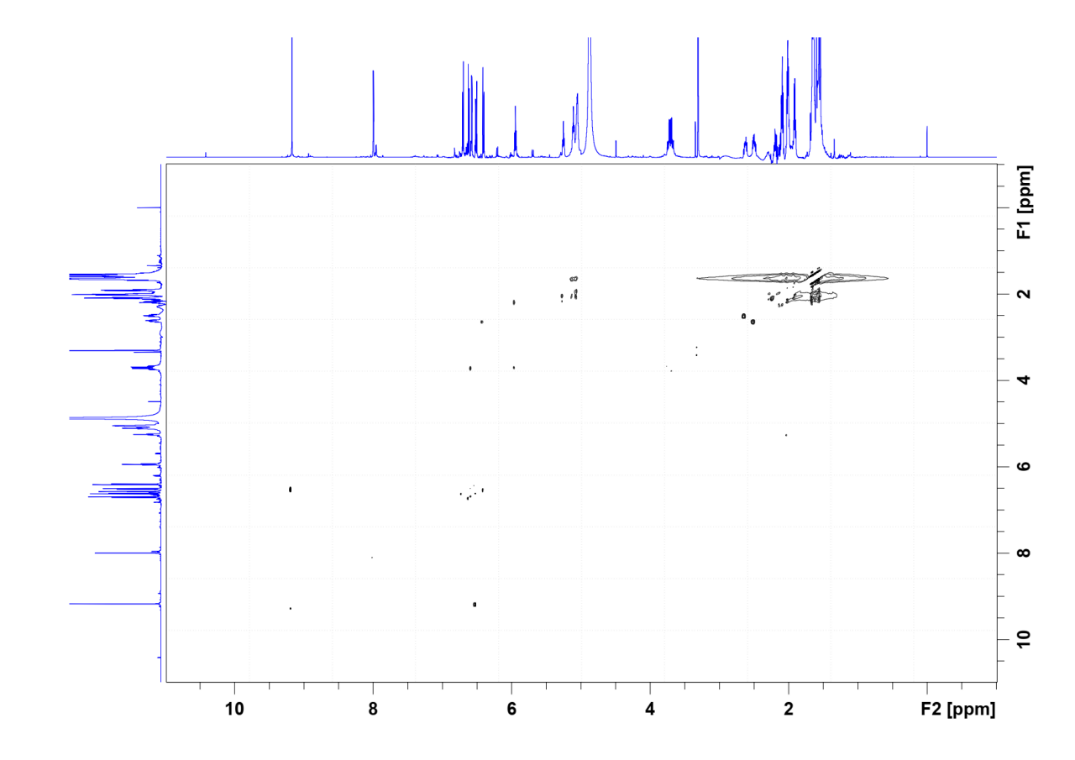


**Figure S43.** ROESY spectrum of compound **4** in methanol-*d*_4_.

[M-H]^-^ m/z 667.3620

| Hit | Formula | m/z | RDB | ppm |
| --- | --- | --- | --- | --- |
| 1 | C_42_H_51_O_7_ | 667.3640 | 17.0 | -3.0 |

**Figure S44.** HRESIMS of **4**.


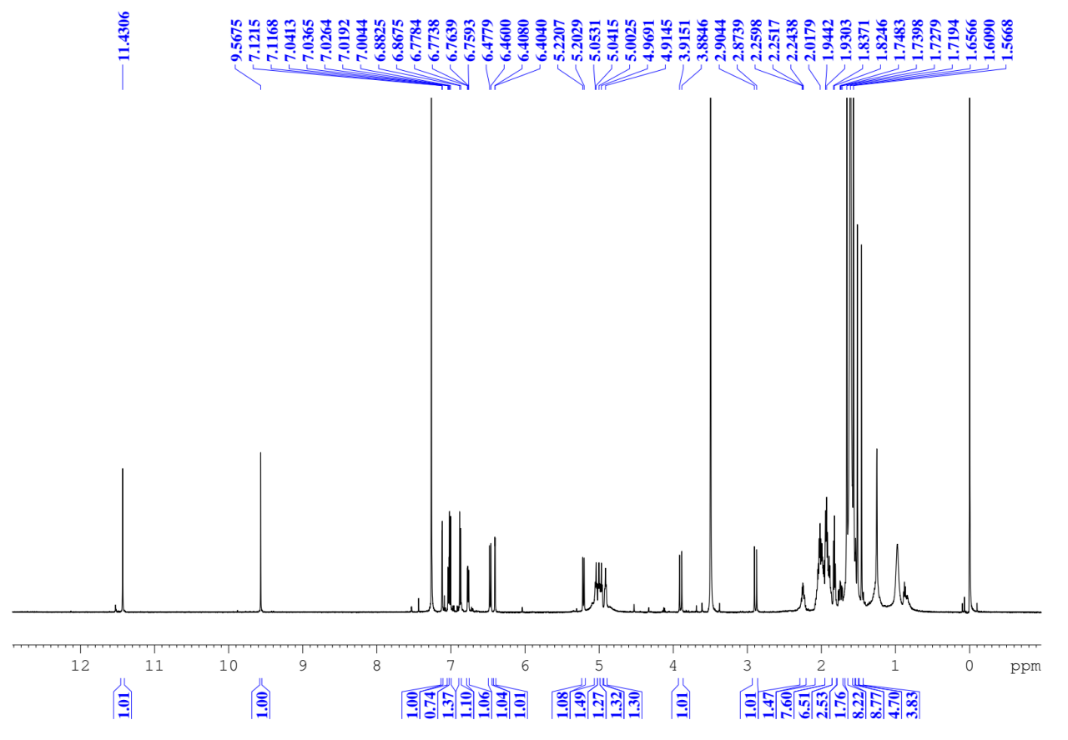


**Figure S45.** ^1^H NMR (600 MHz) spectrum of compound **5** in CDCl_3_.


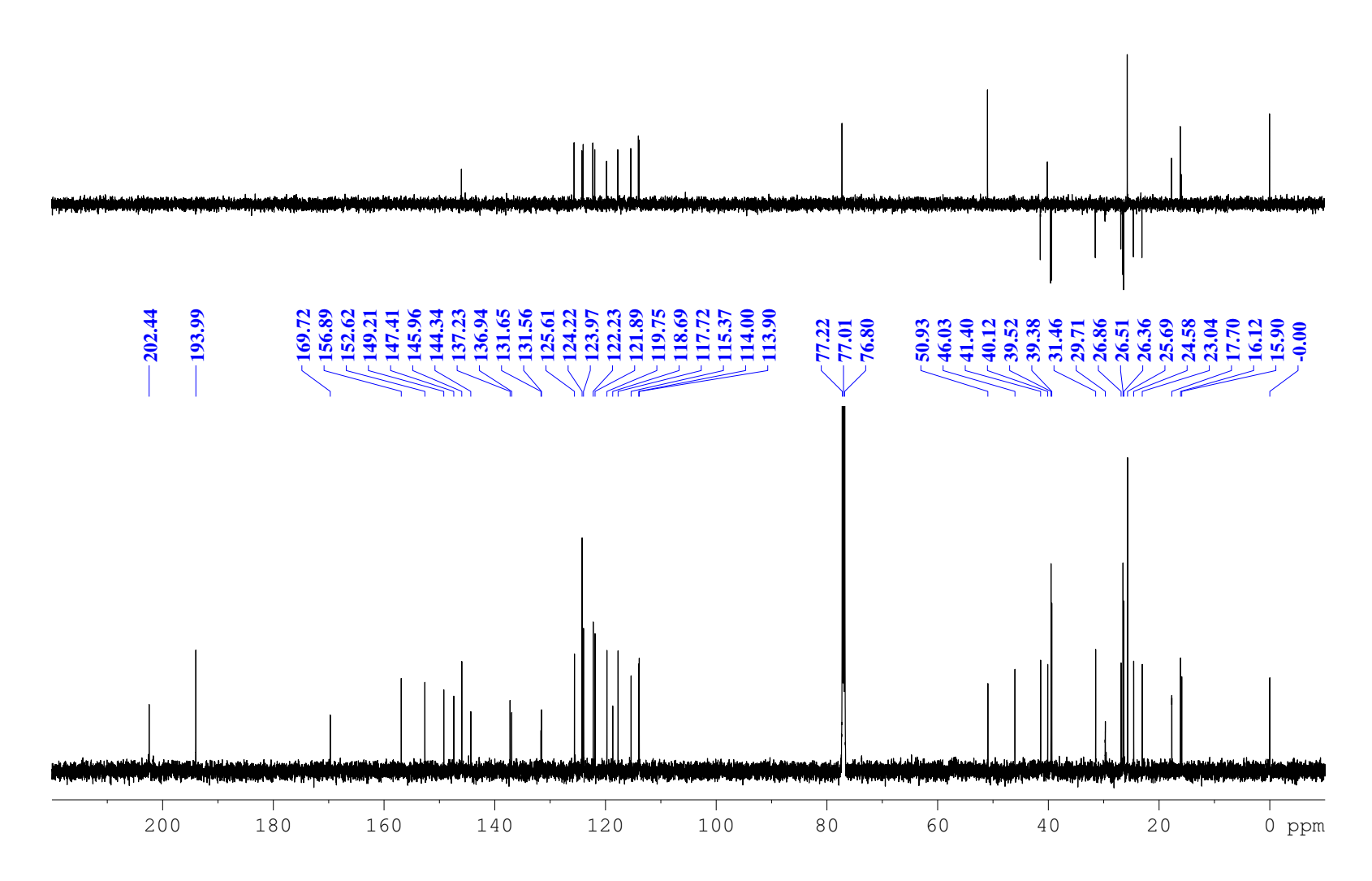


**Figure S46.** ^13^C NMR (150 MHz) and DEPT spectra of compound **5** in CDCl_3_.


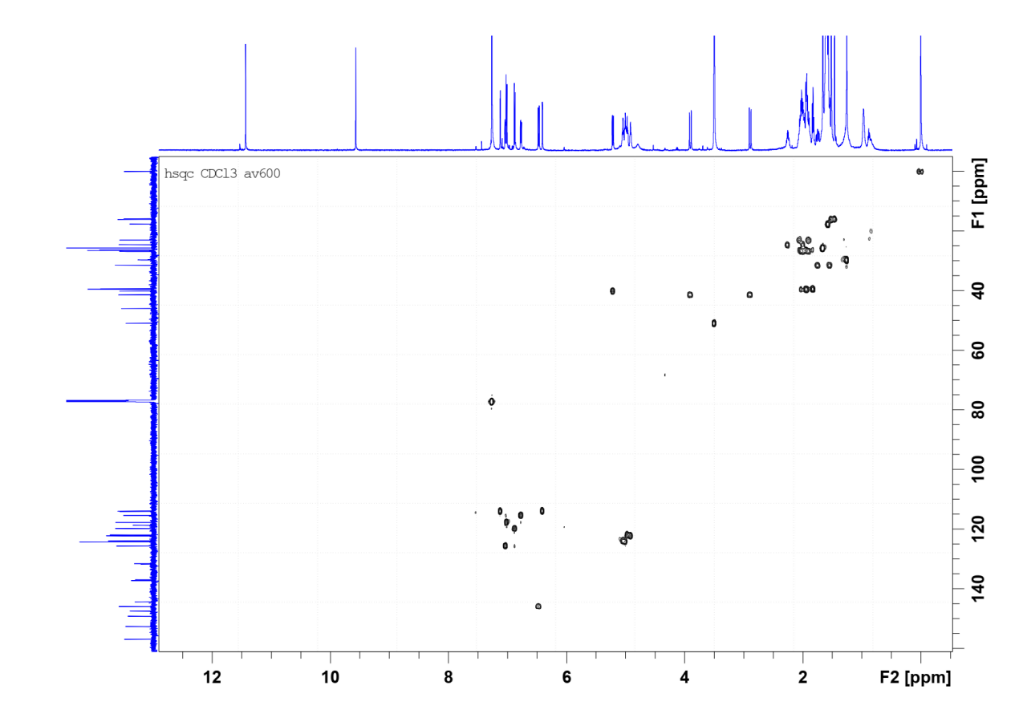


**Figure S47.** HSQC spectrum of compound **5** in CDCl_3_.


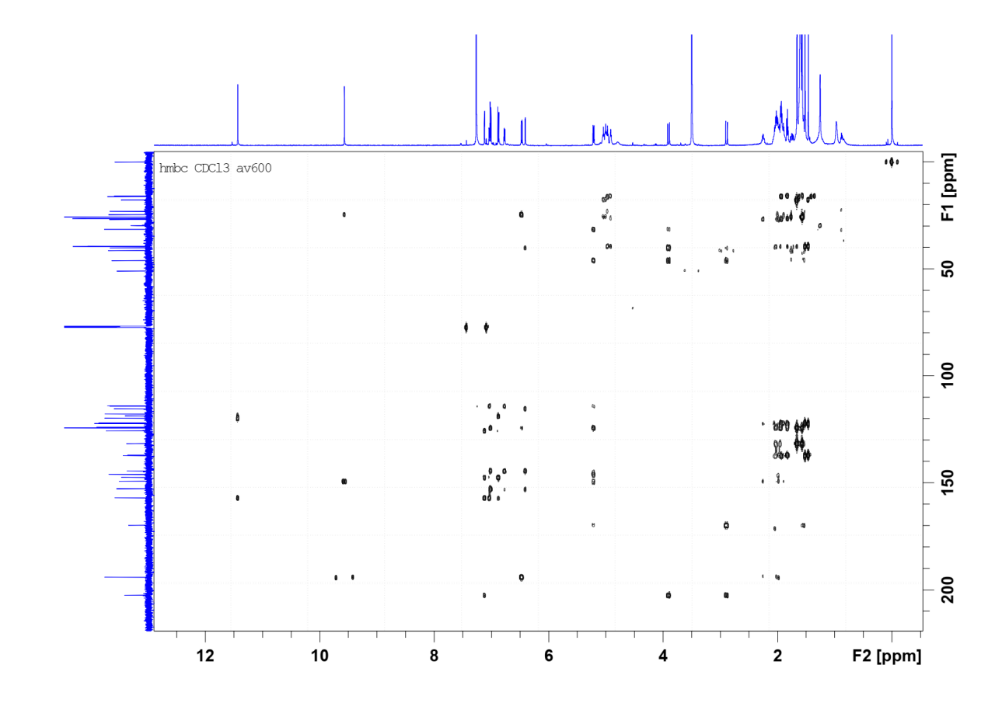


**Figure S48.** HMBC spectrum of compound **5** in CDCl_3_.


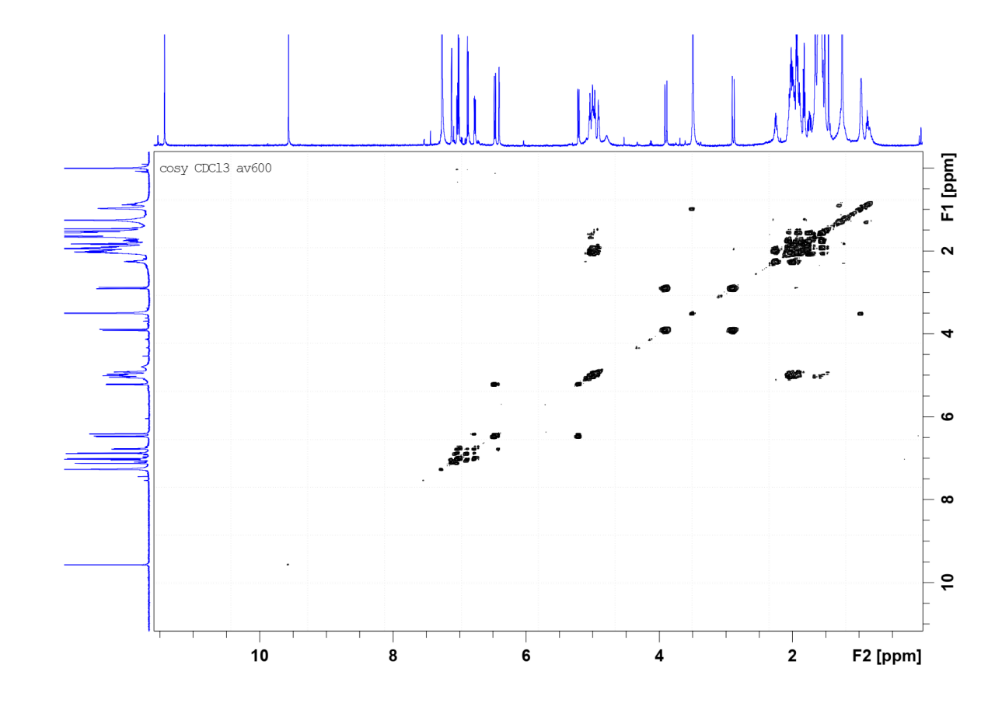


**Figure S49.** COSY spectrum of compound **5** in CDCl_3_.


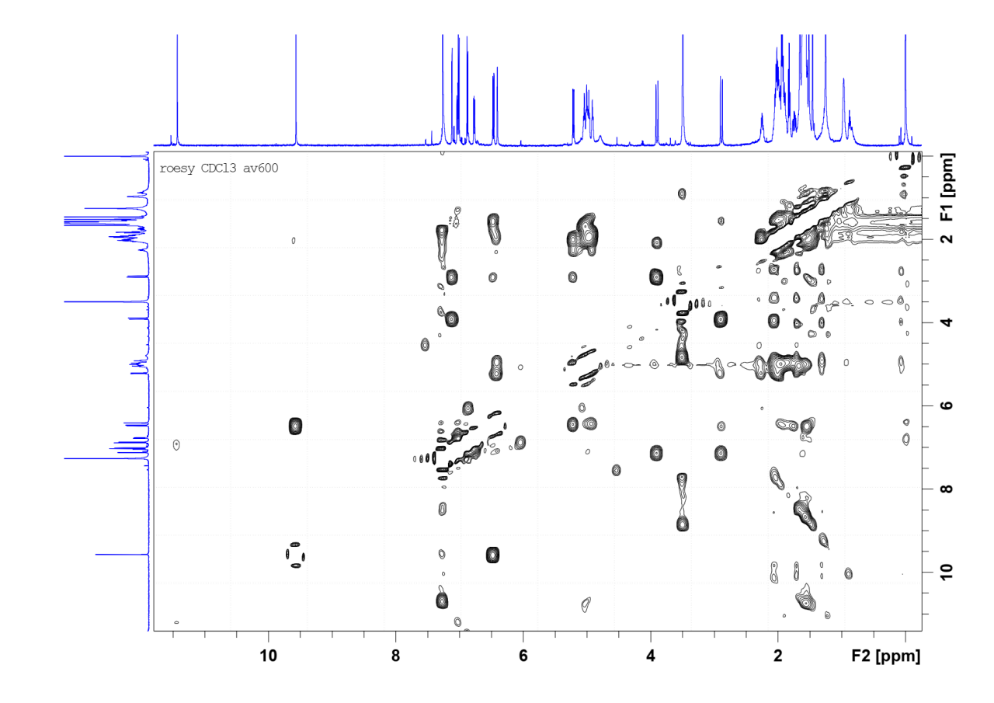


**Figure S50.** ROESY spectrum of compound **5** in CDCl_3_.


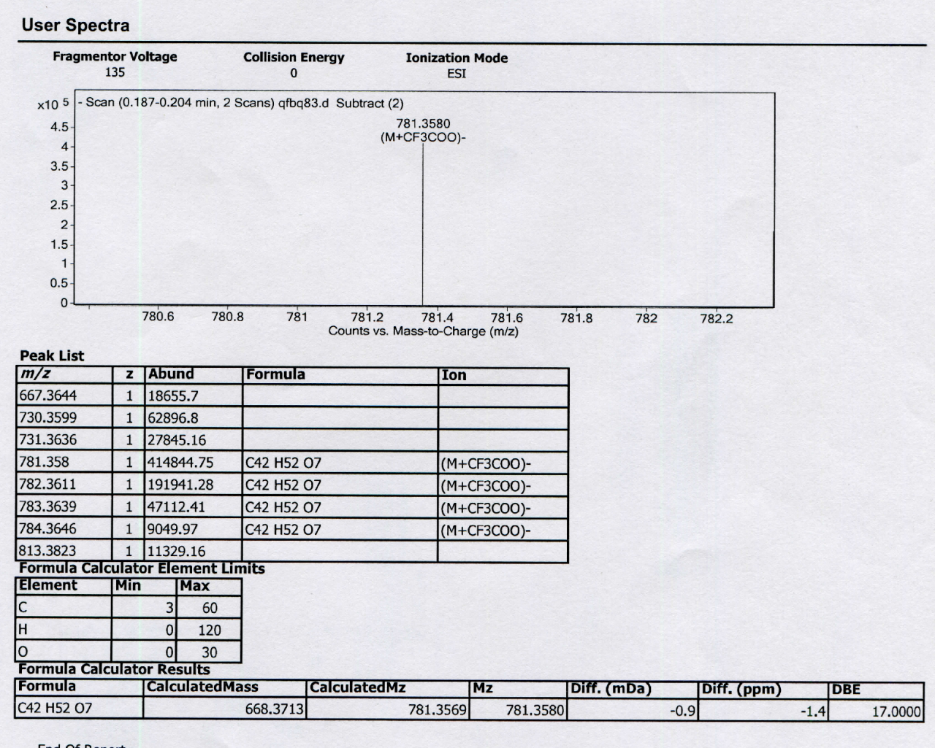


**Figure S51.** HRESIMS of **5**.


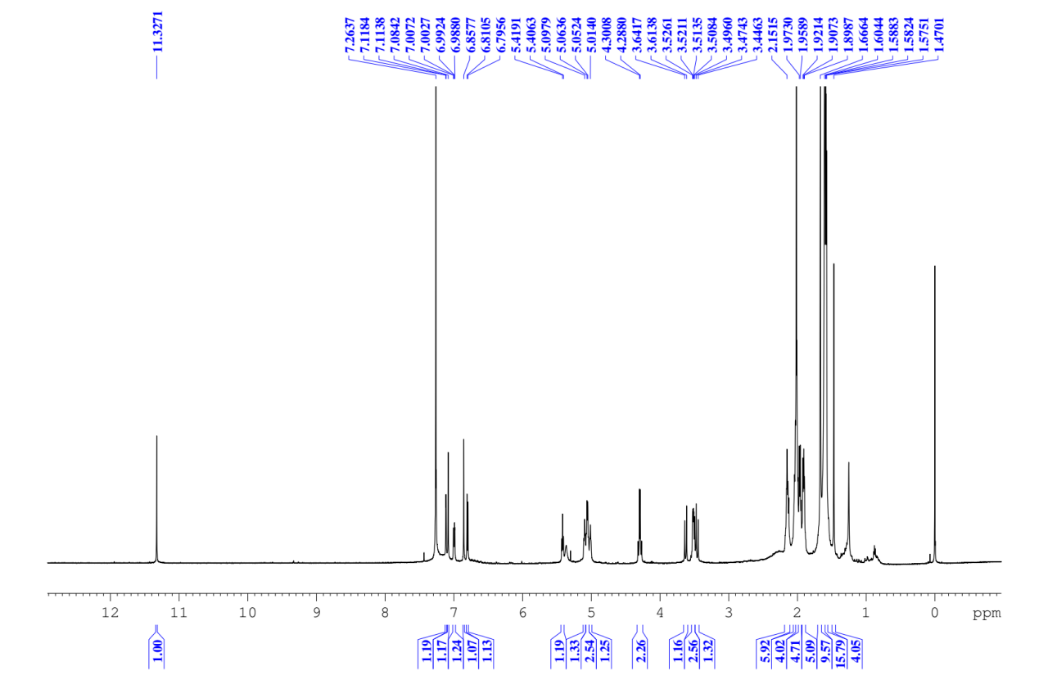


**Figure S52.** ^1^H NMR (600 MHz) spectrum of compound **6** in CDCl_3_.


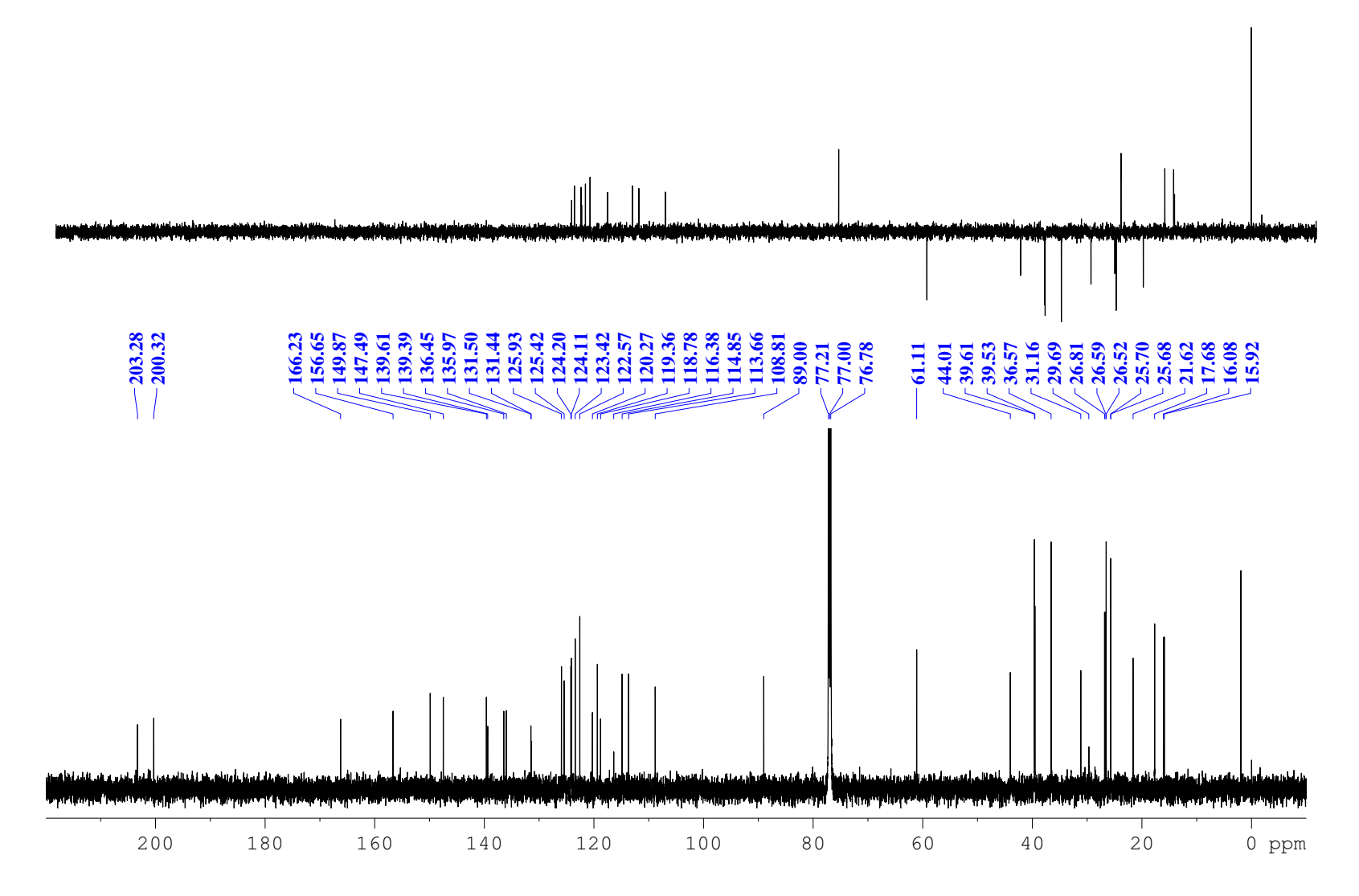


**Figure S53.** ^13^C NMR (150 MHz) and DEPT spectra of compound **6** in CDCl_3_.


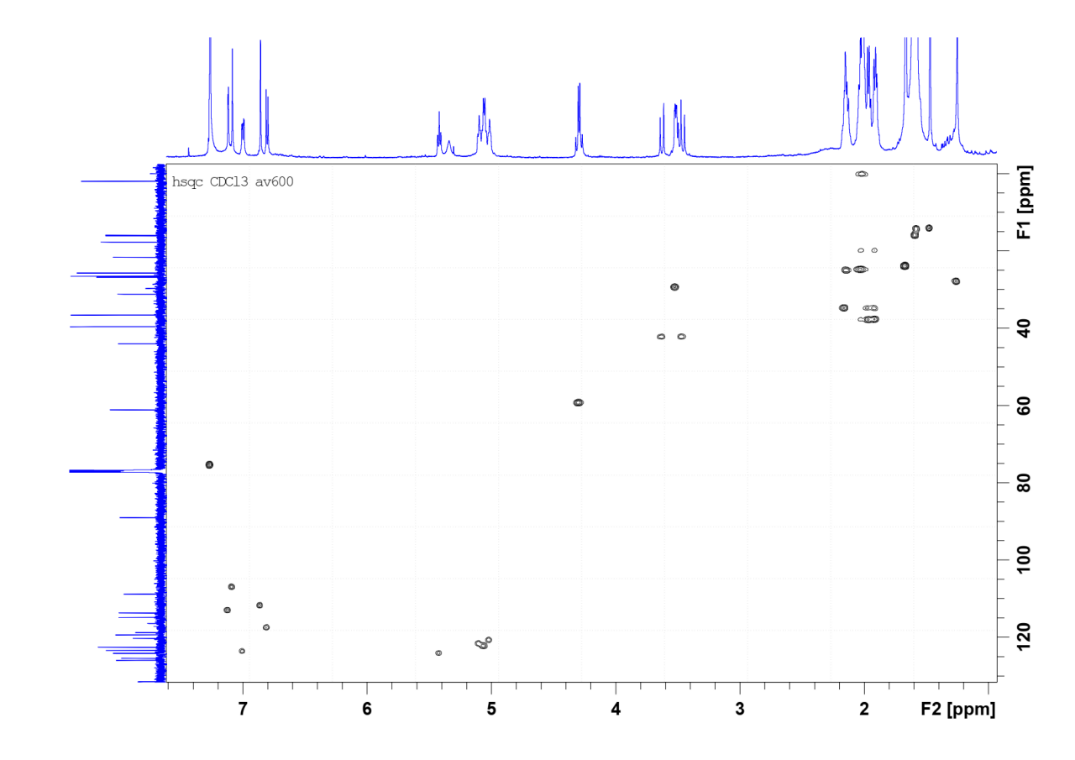


**Figure S54.** HSQC spectrum of compound **6** in CDCl_3_.


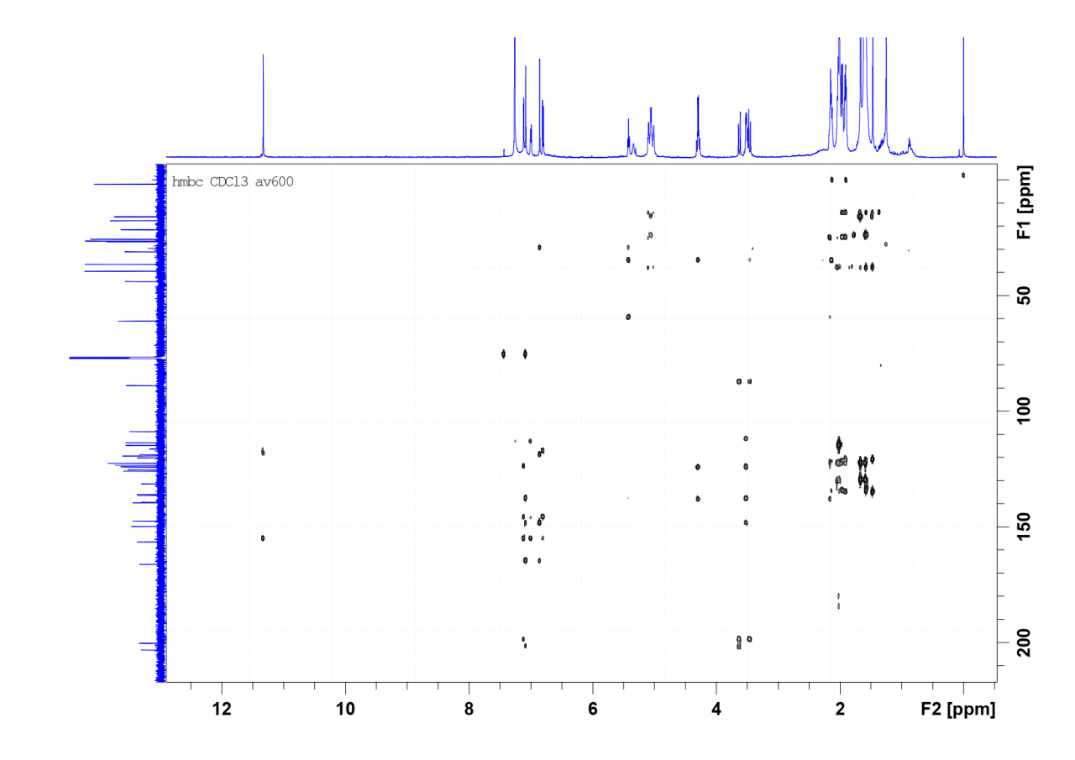


**Figure S55.** HMBC spectrum of compound **6** in CDCl_3_.


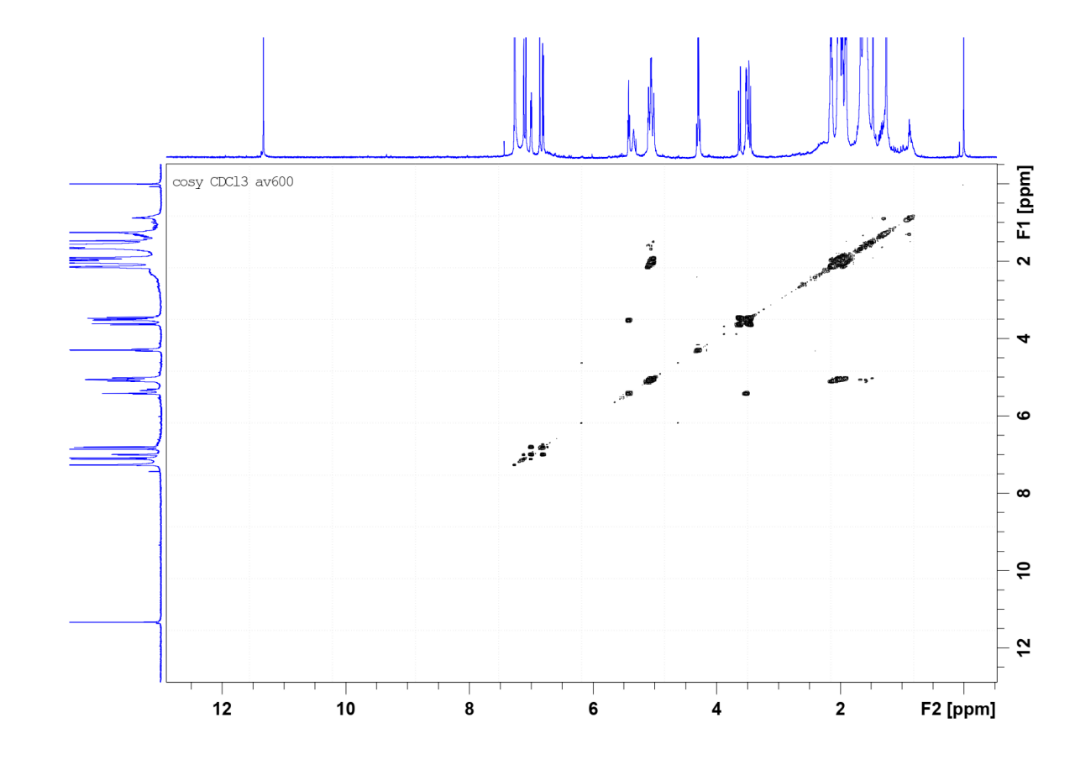


**Figure S56.** COSY spectrum of compound **6** in CDCl_3_.


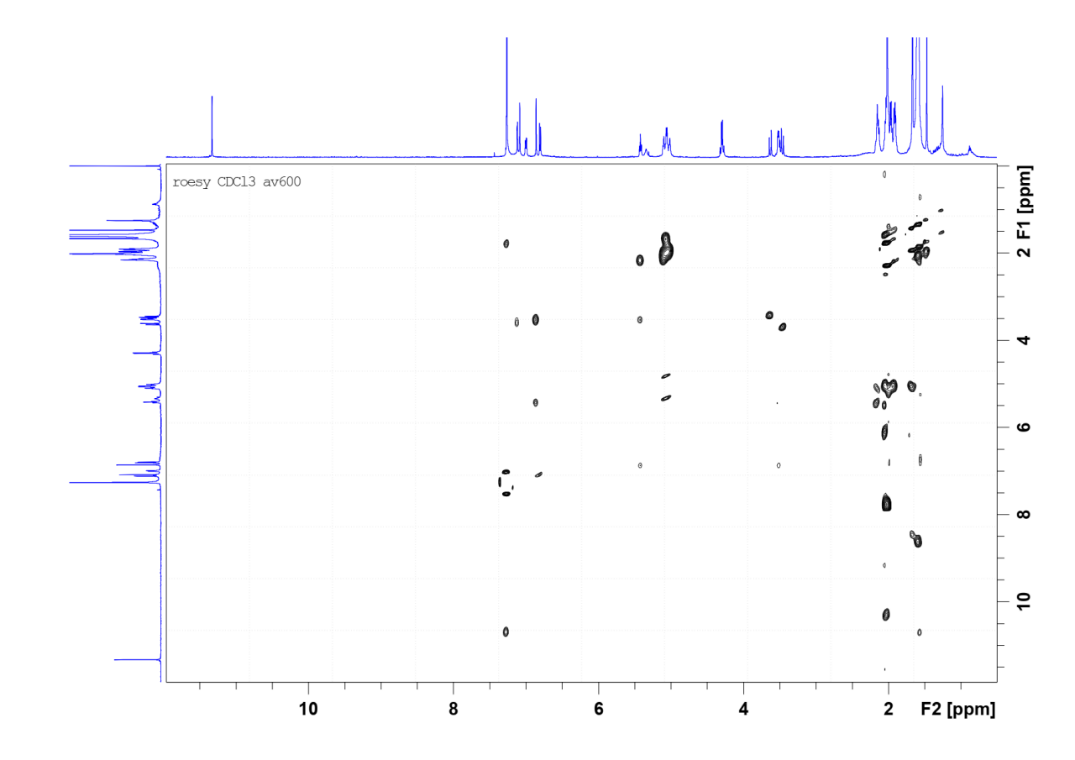


**Figure S57.** ROESY spectrum of compound **6** in CDCl_3_.


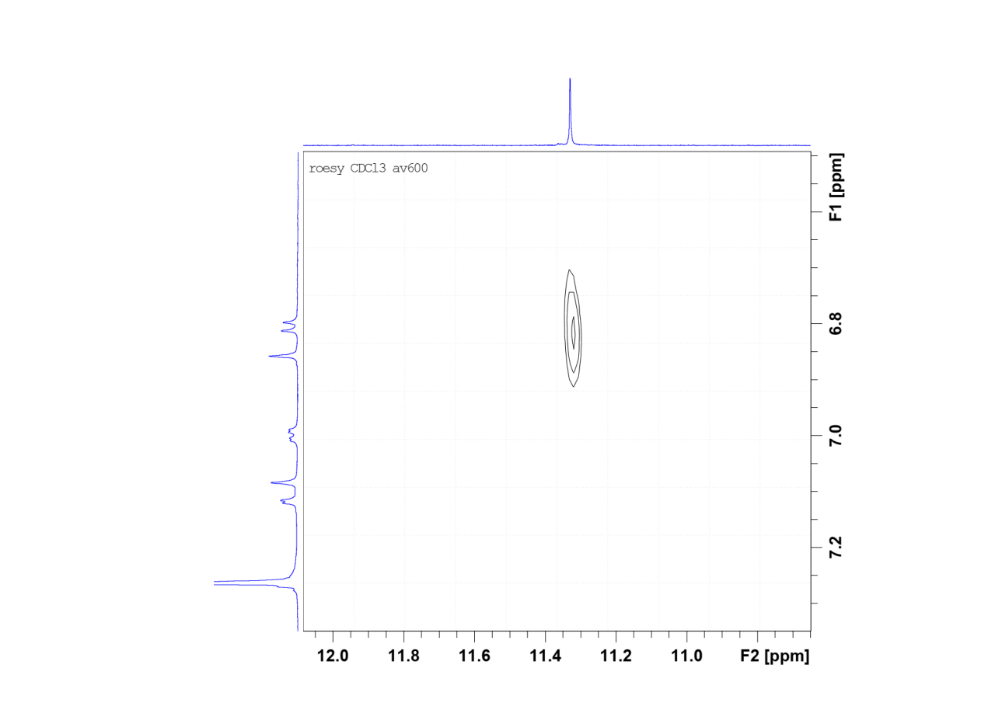


**Figure S58.** Enlarge ROESY spectrum of compound **6** in CDCl_3_.


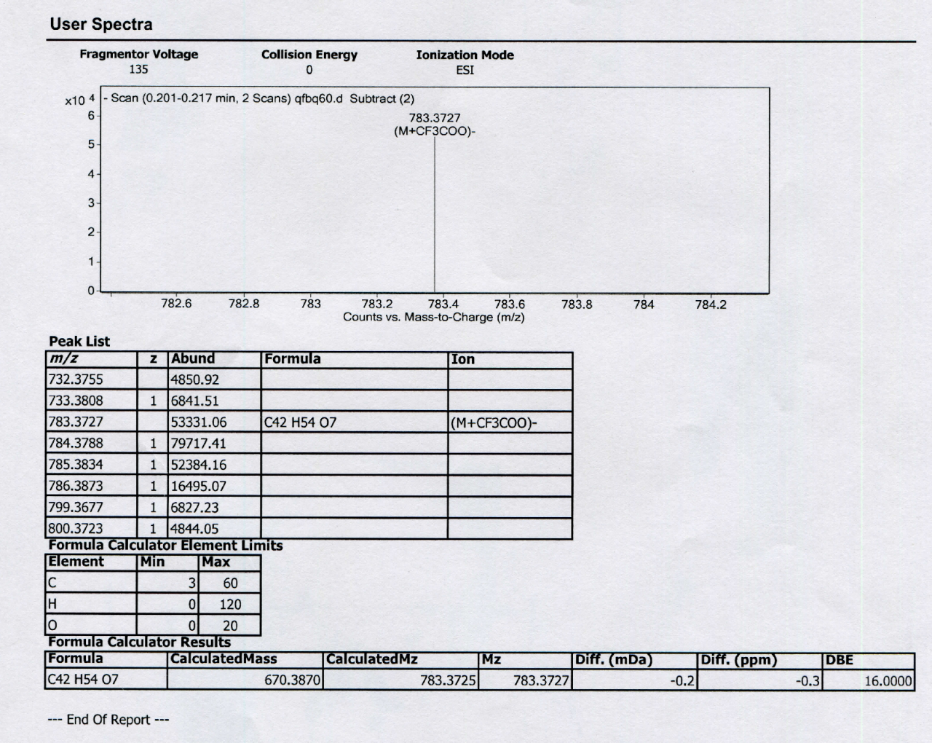


**Figure S59.** HRESIMS of **6**.


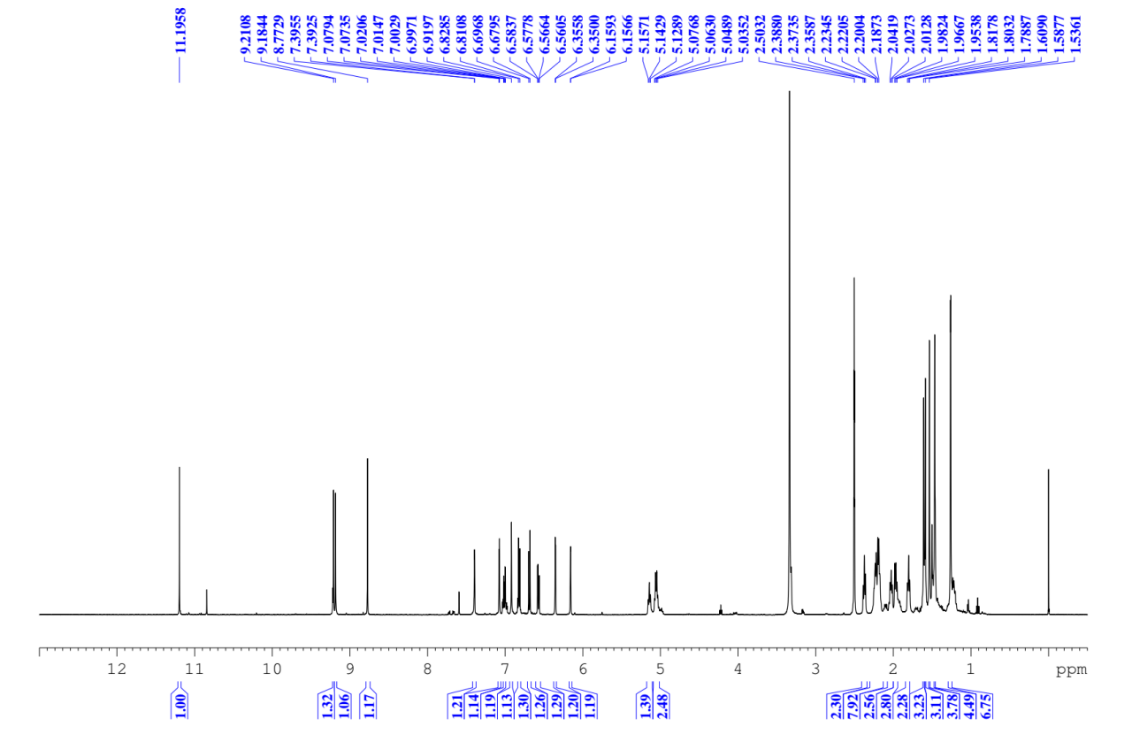


**Figure S60.** ^1^H NMR (500 MHz) spectrum of compound **7** in DMSO-*d*_6_.


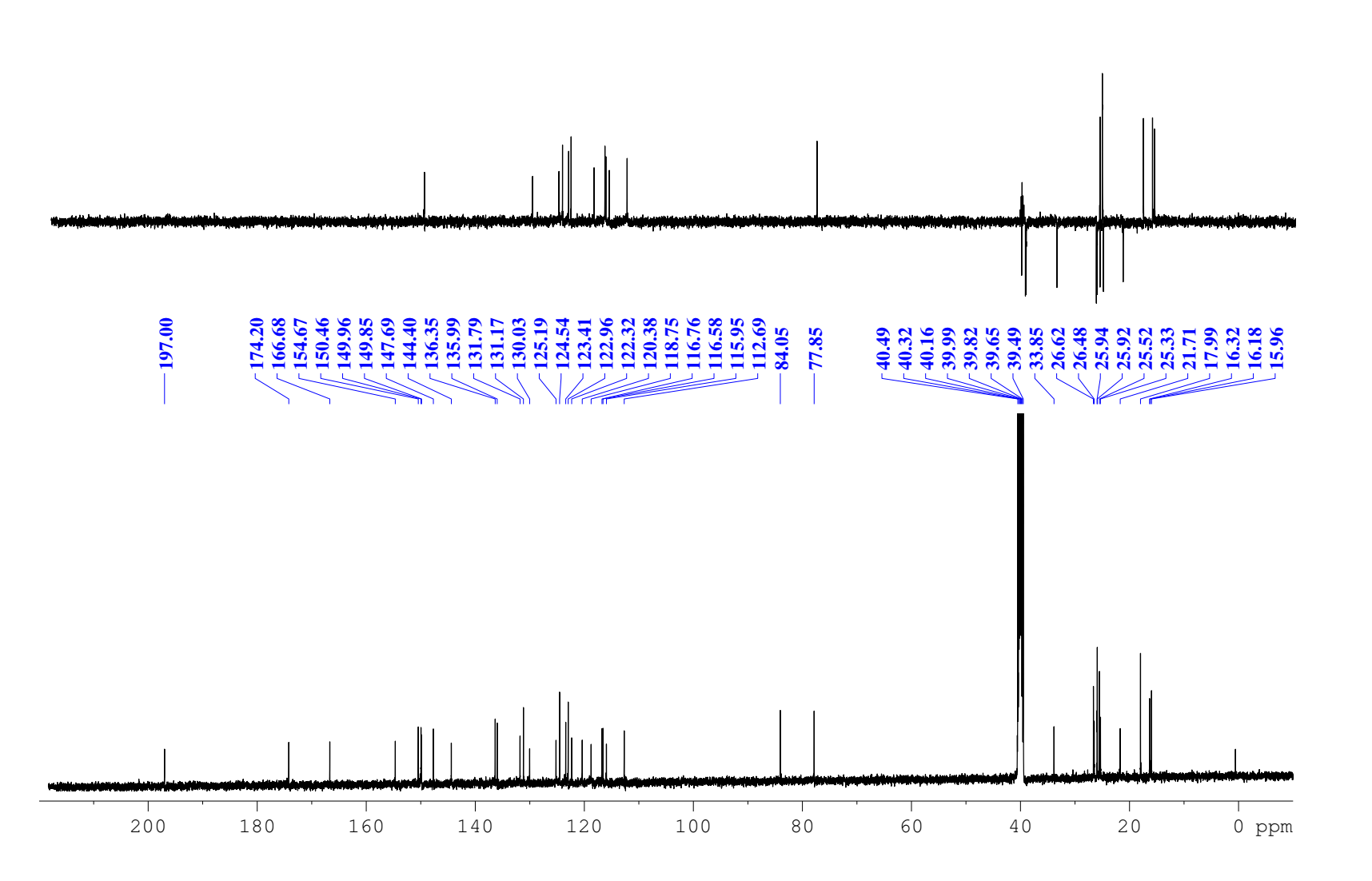


**Figure S61.** ^13^C NMR (125 MHz) and DEPT spectra of compound **7** in DMSO-*d*_6_.


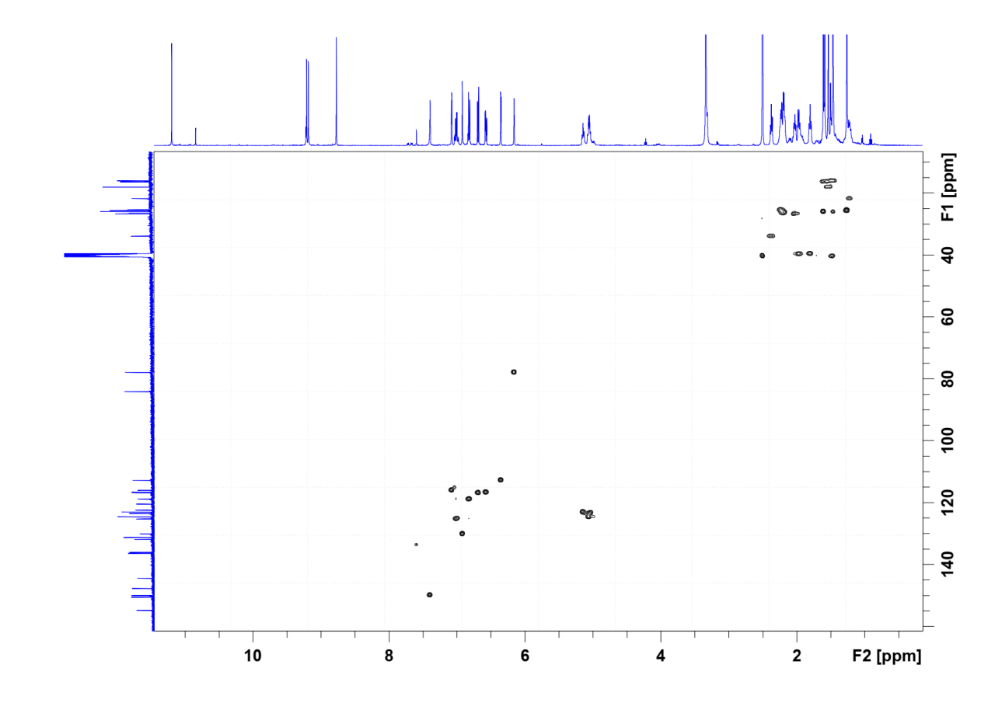


**Figure S62.** HSQC spectrum of compound **7** in DMSO-*d*_6_.


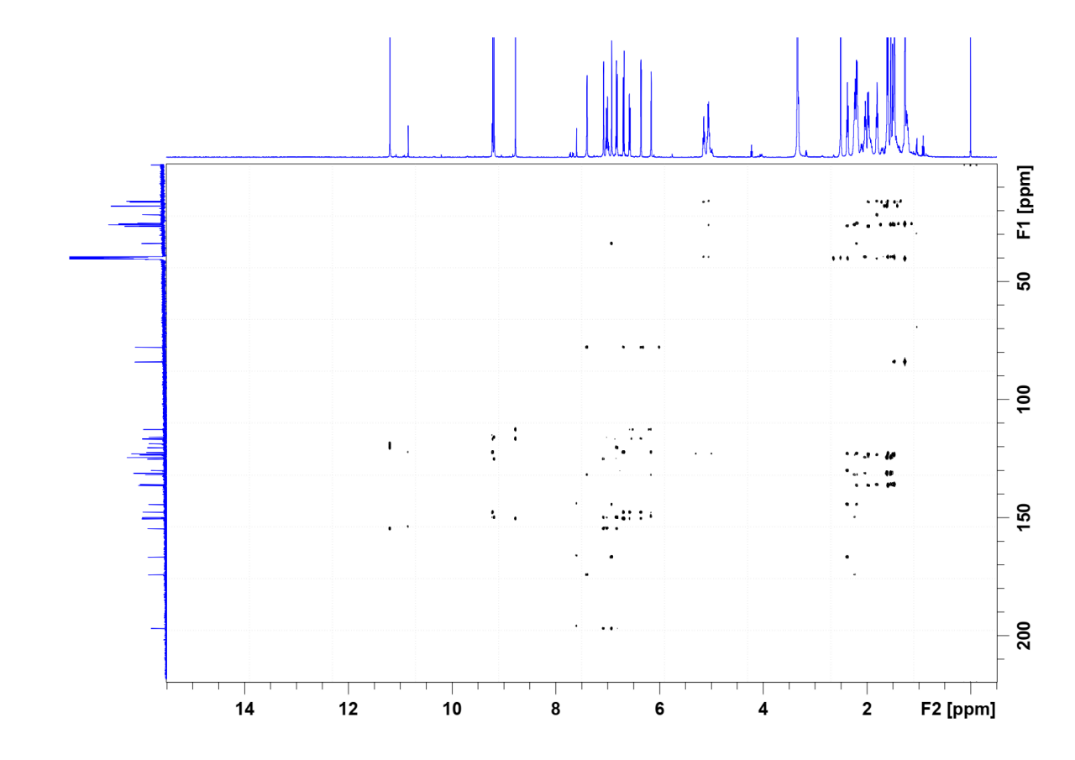


**Figure S63.** HMBC spectrum of compound **7** in DMSO-*d*_6_.


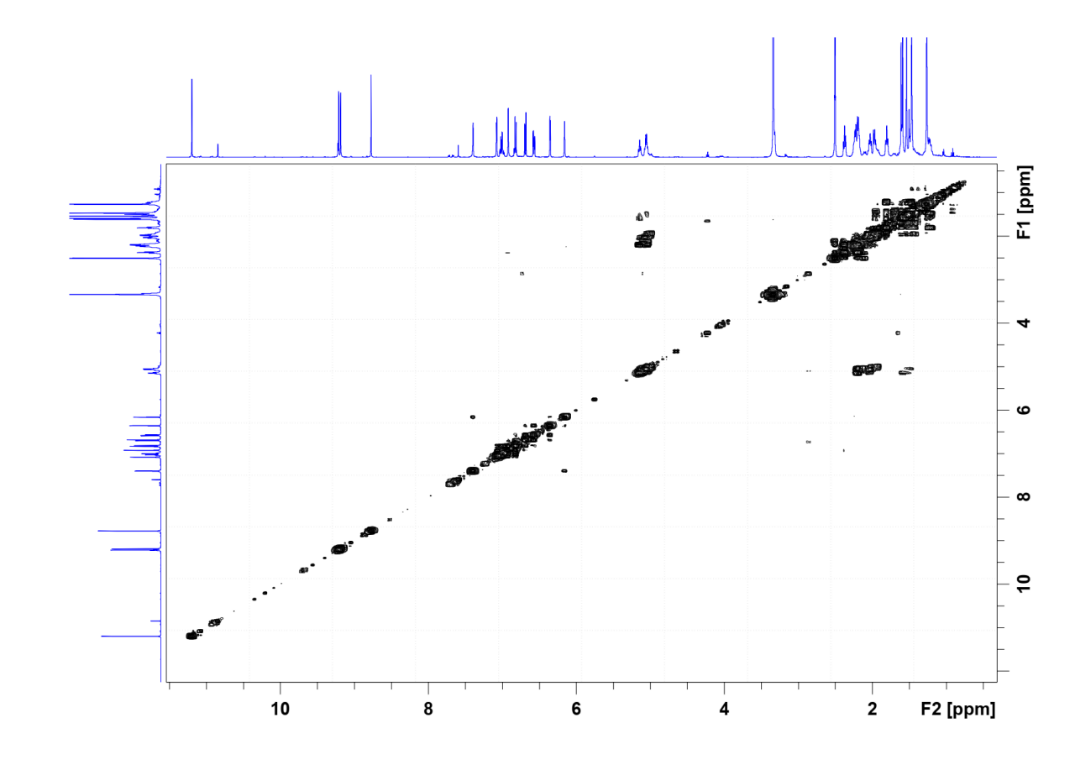


**Figure S64.** COSY spectrum of compound **7** in DMSO-*d*_6_.


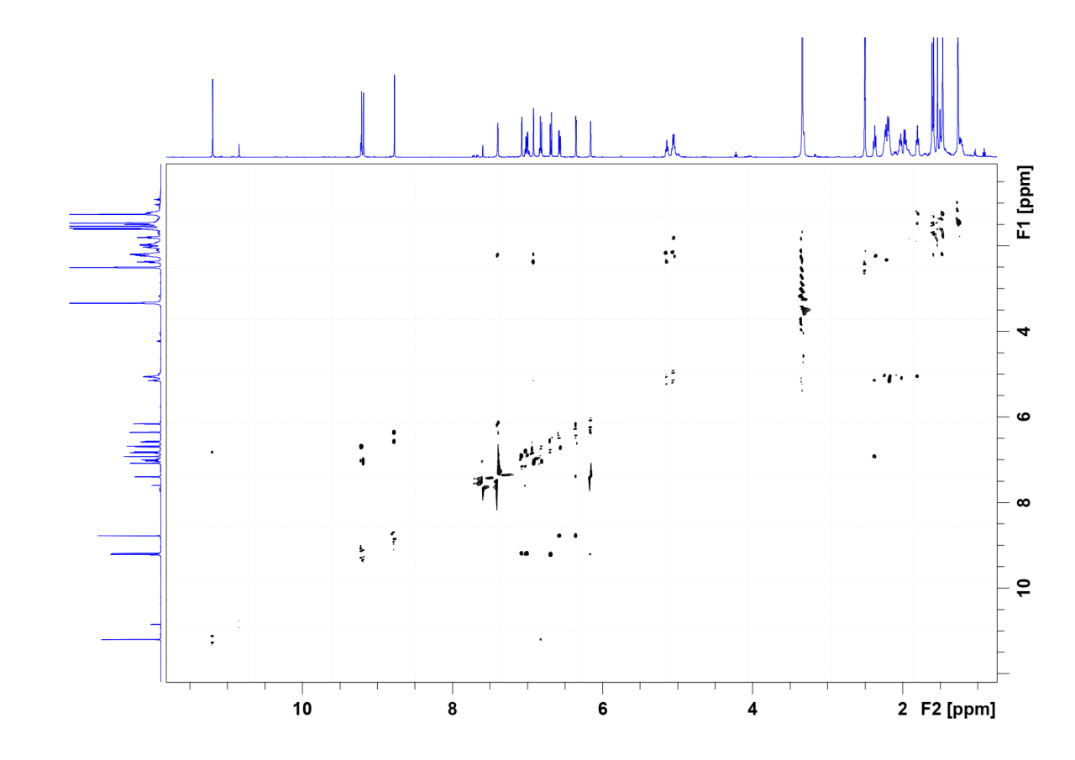


**Figure S65.** ROESY spectrum of compound **7** in DMSO-*d*_6_.


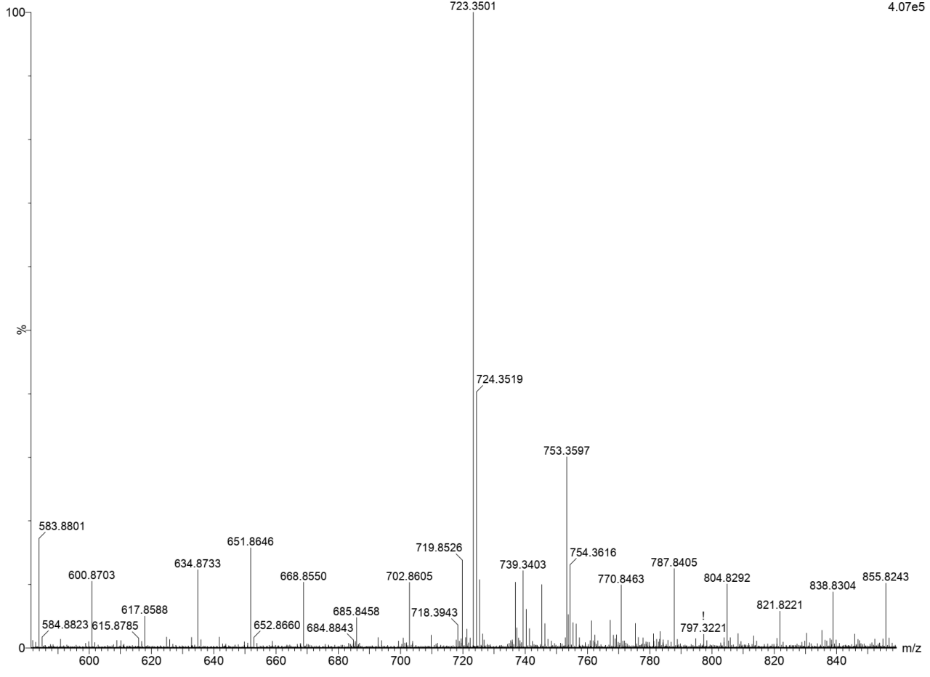


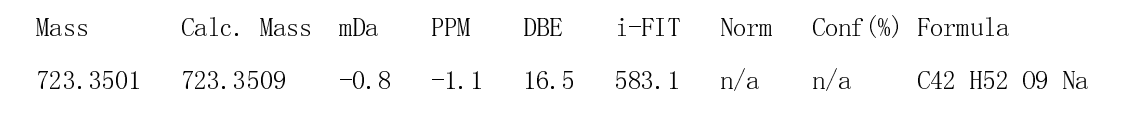


**Figure S66.** HRESIMS of **7**.

**Figure S67**. CD spectrum of (+)-**7**.

**Figure S68**. CD spectrum of (–)-**7**.


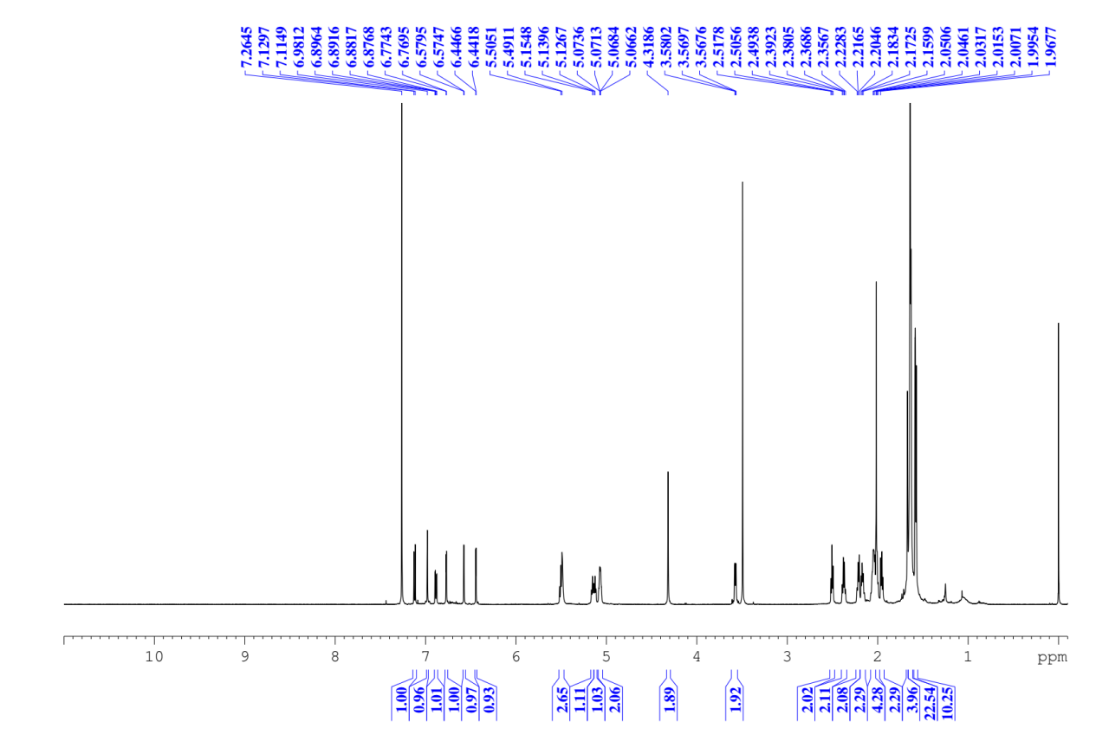


**Figure S69.** ^1^H NMR (600 MHz) spectrum of compound **8** in CDCl_3_.


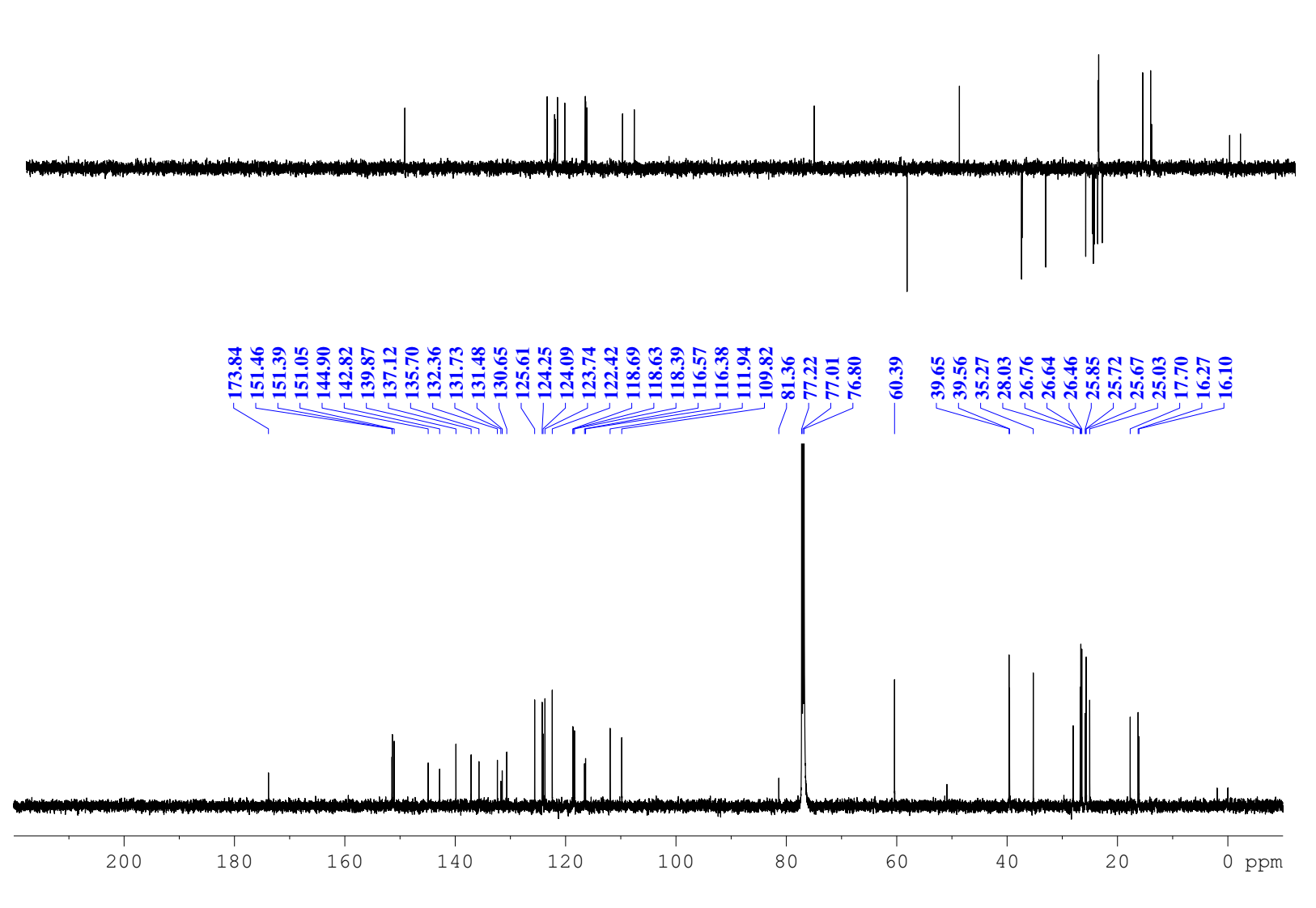


**Figure S70.** ^13^C NMR (150 MHz) and DEPT spectra of compound **8** in CDCl_3_.


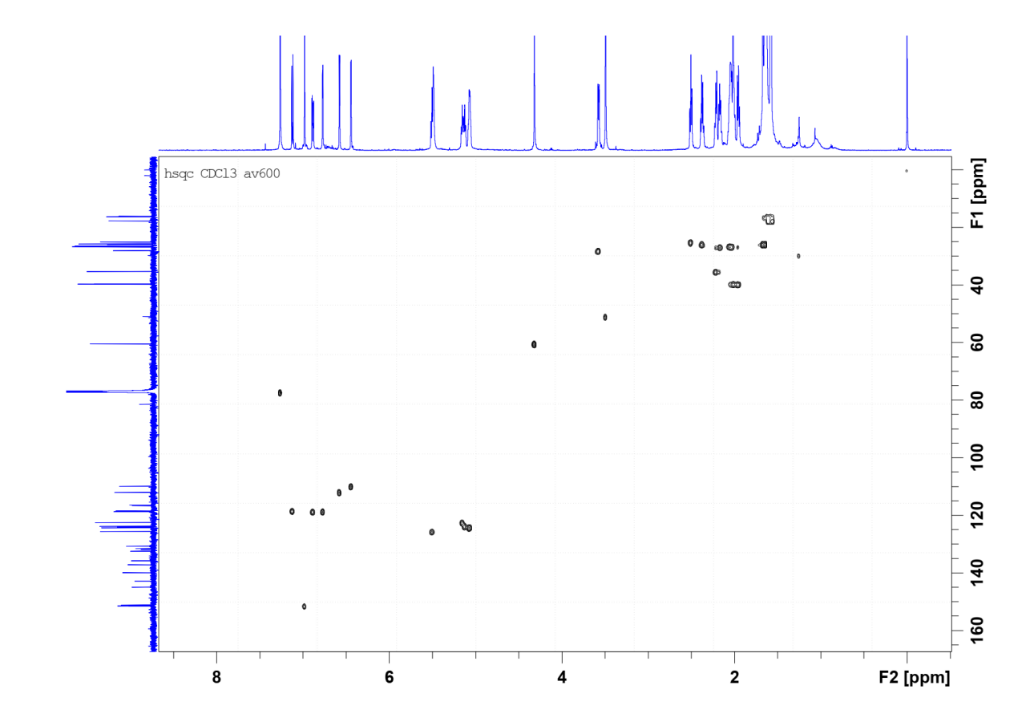


**Figure S71.** HSQCspectrum of compound **8** in CDCl_3_.


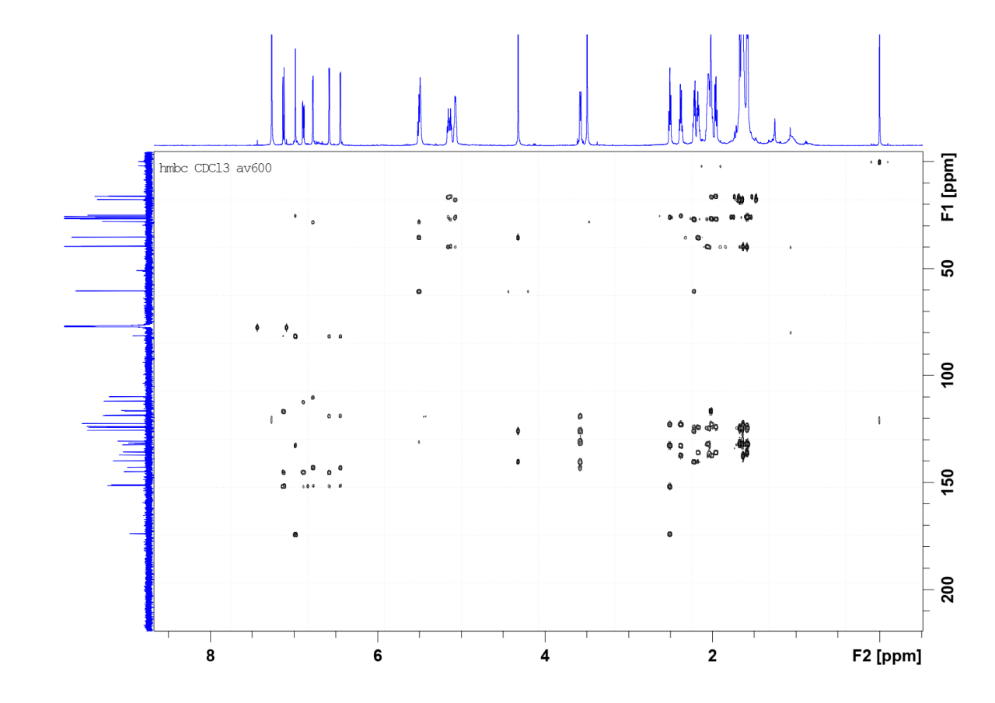


**Figure S72.** HMBC spectrum of compound **8** in CDCl_3_.


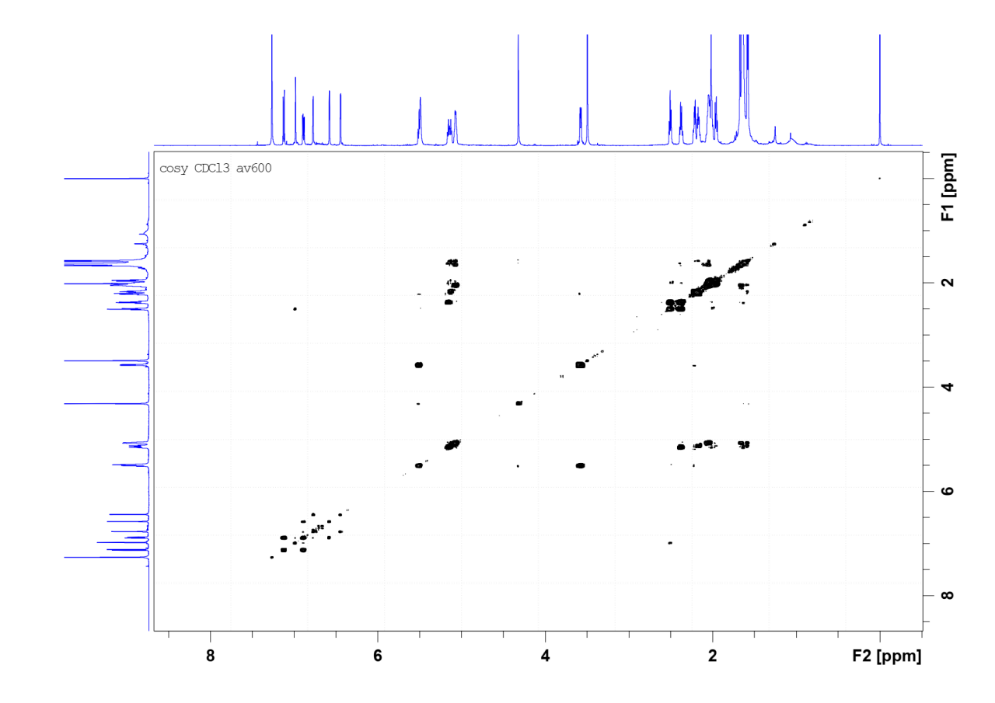


**Figure S73.** COSY spectrum of compound **8** in CDCl_3_.


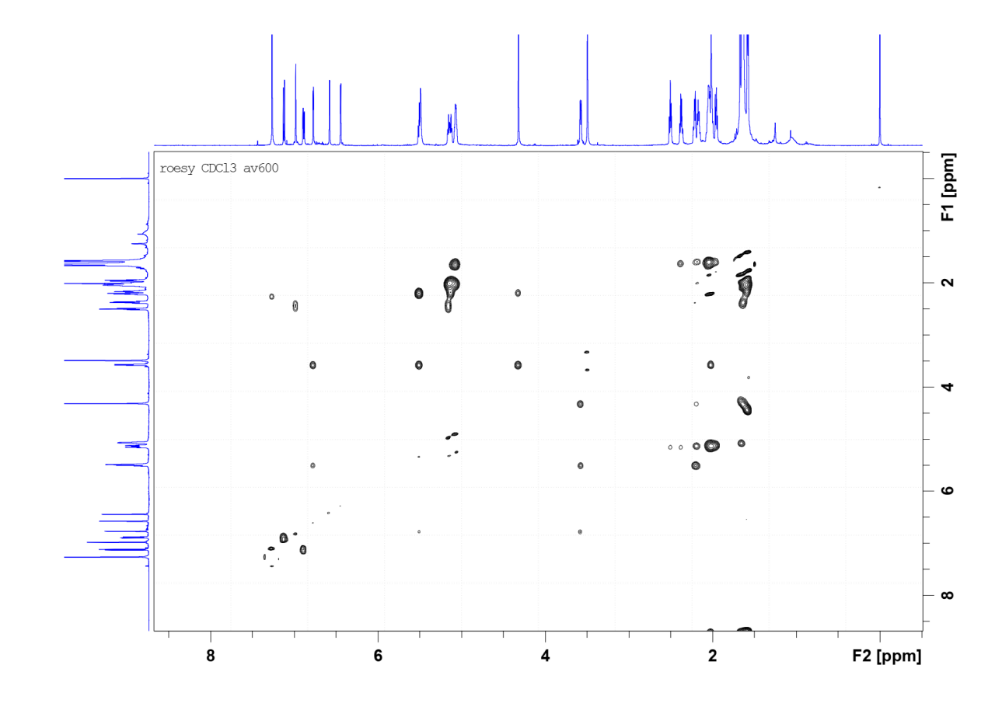


**Figure S74.** ROESY spectrum of compound **8** in CDCl_3_.


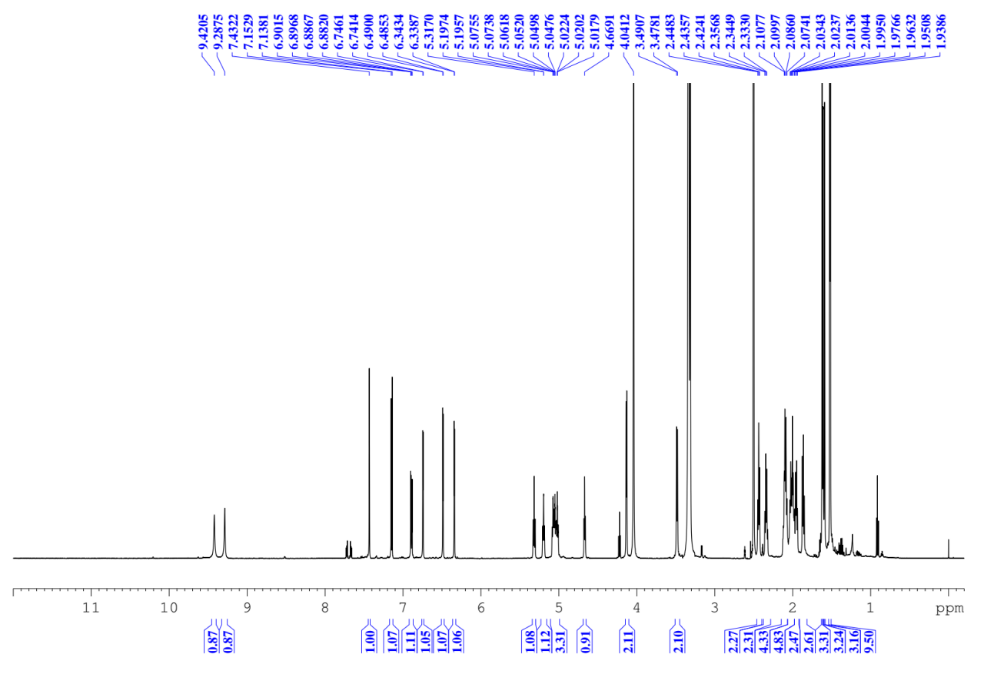


**Figure S75.** ^1^H NMR spectrum of compound **8** in DMSO-*d*_6_.


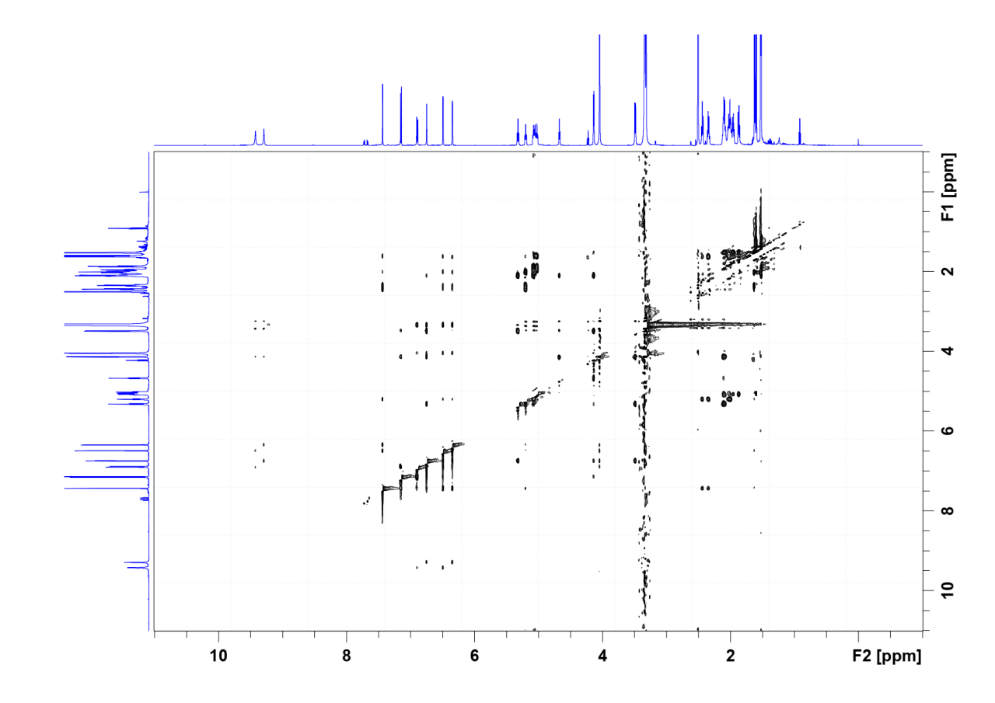


**Figure S76.** ROESY spectrum of compound **8** in DMSO-*d*_6_.


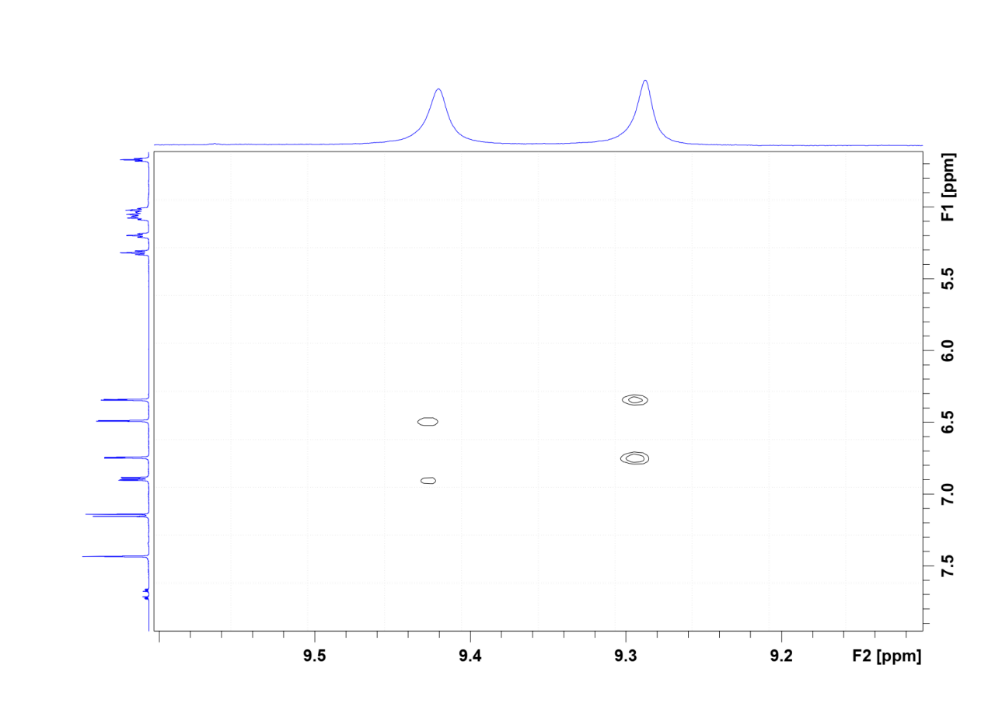


**Figure S77.** Enlarge ROESY spectrum of compound **8** in DMSO-*d*_6_.


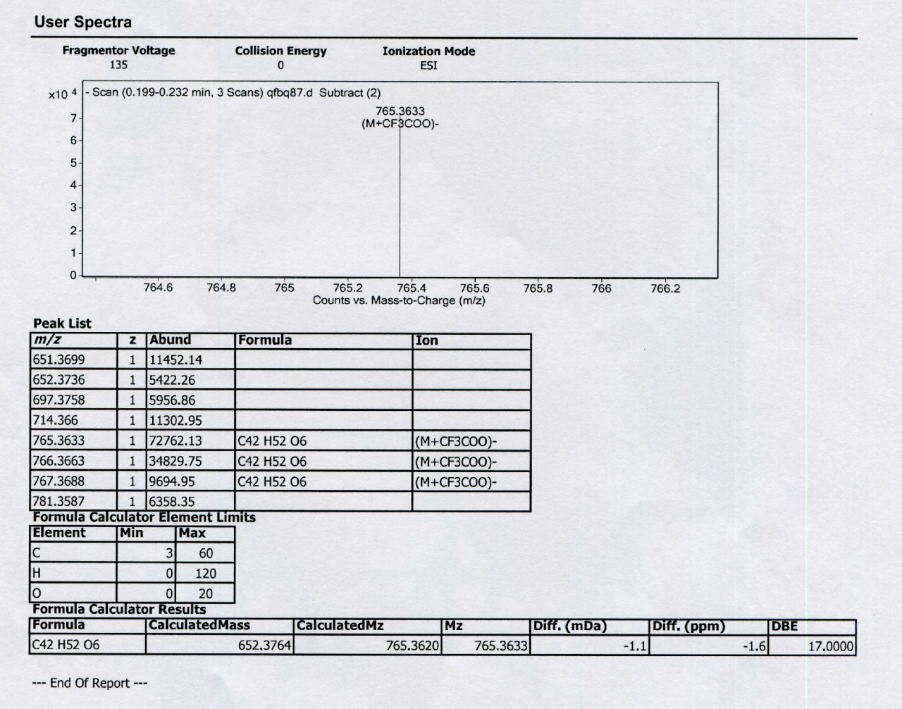


**Figure S78**. HRESIMS of **8**.


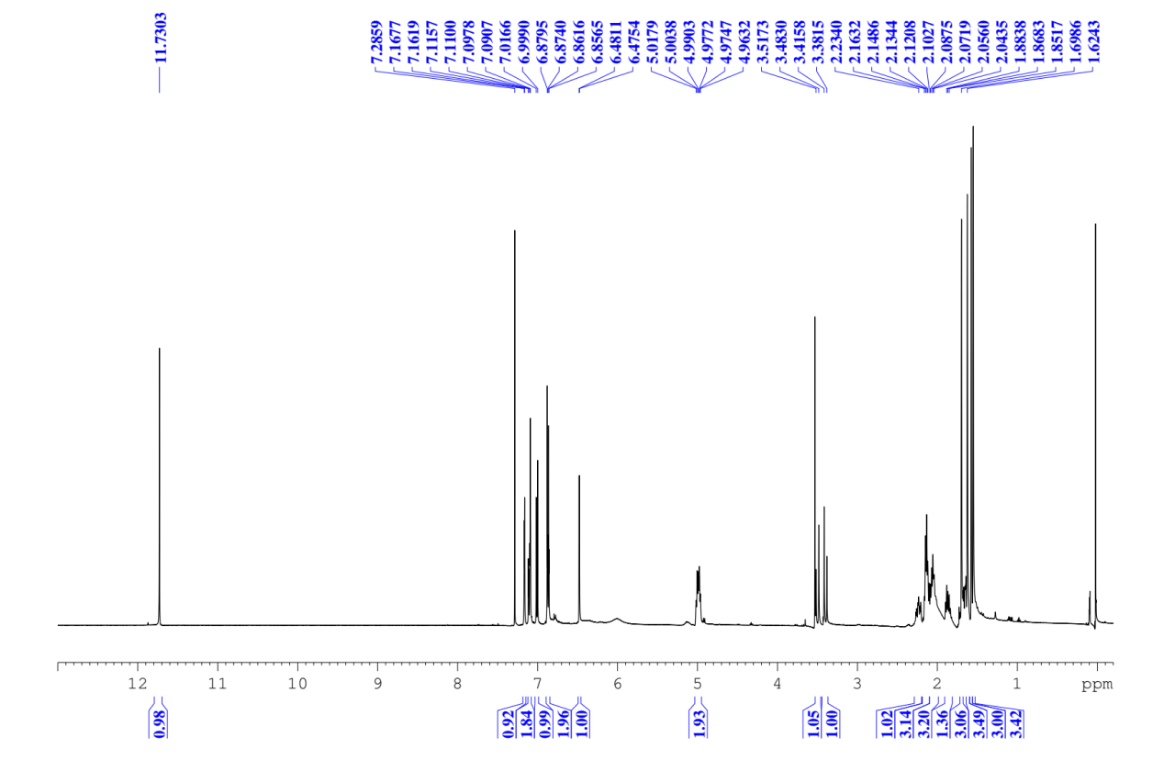


**Figure S79.** ^1^H NMR (500 MHz) spectrum of compound **9** in CDCl_3_.


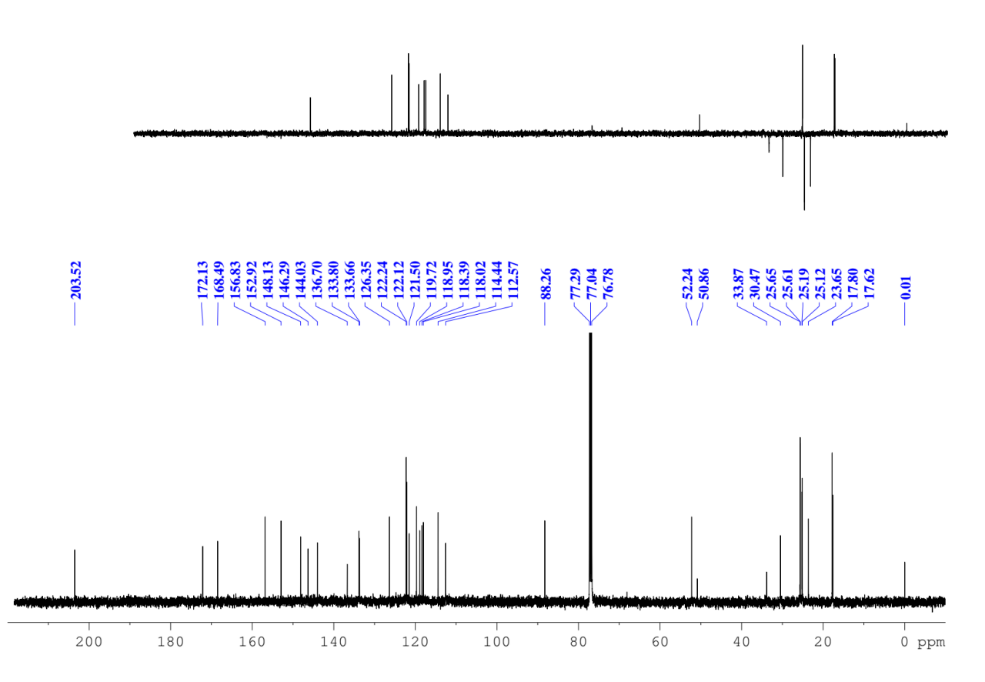


**Figure S80.** ^13^C NMR (125 MHz) and DEPT spectra of compound **9** in CDCl_3_.


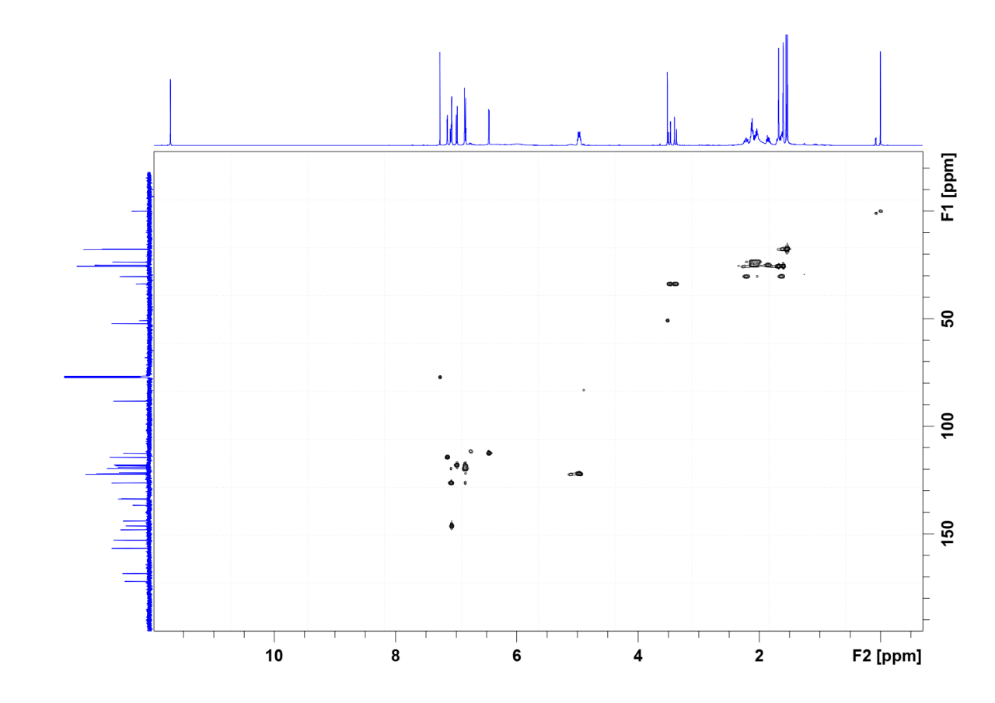


**Figure S81.** HSQC spectrum of compound **9** in CDCl_3_.


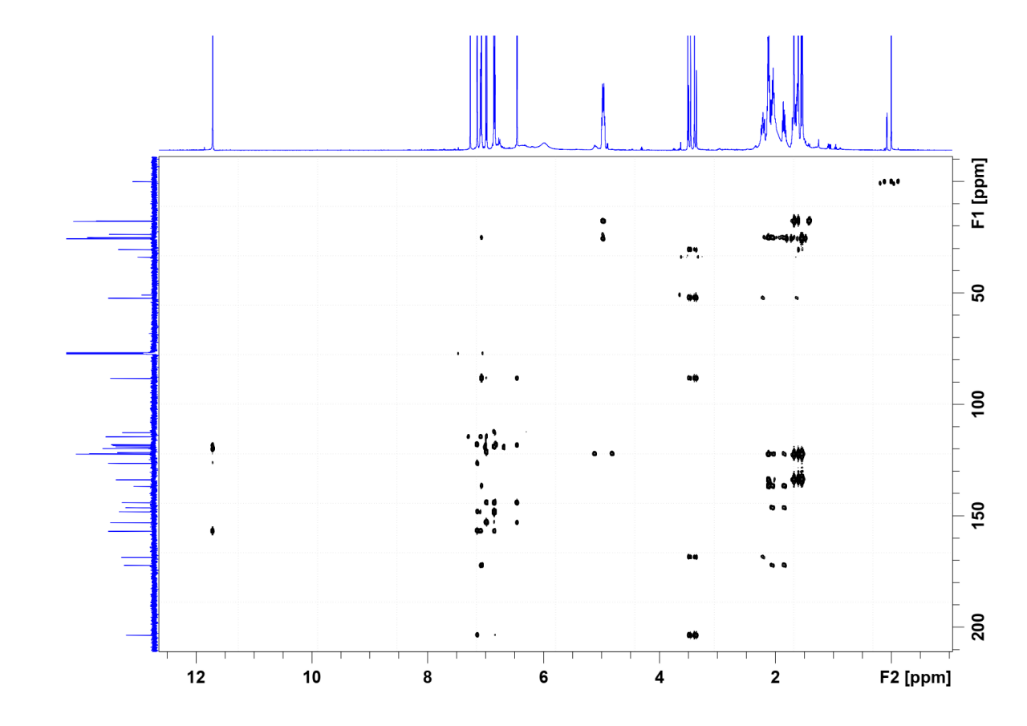


**Figure S82.** HMBC spectrum of compound **9** in CDCl_3_.


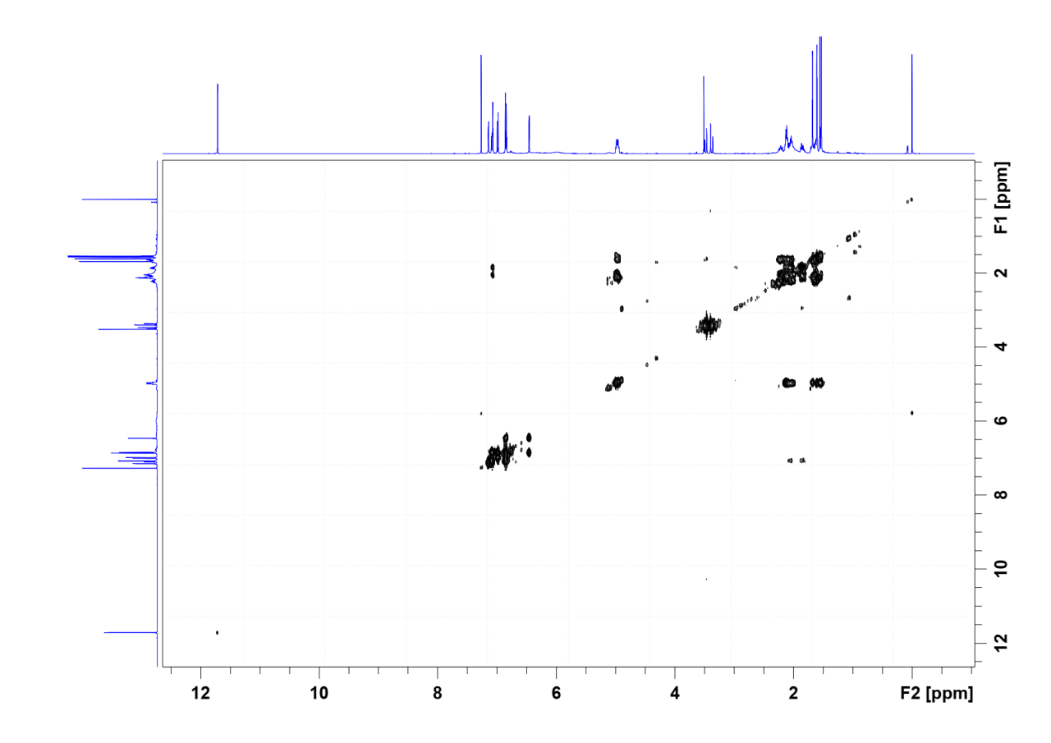


**Figure S83.** COSY spectrum of compound **9** in CDCl_3_.


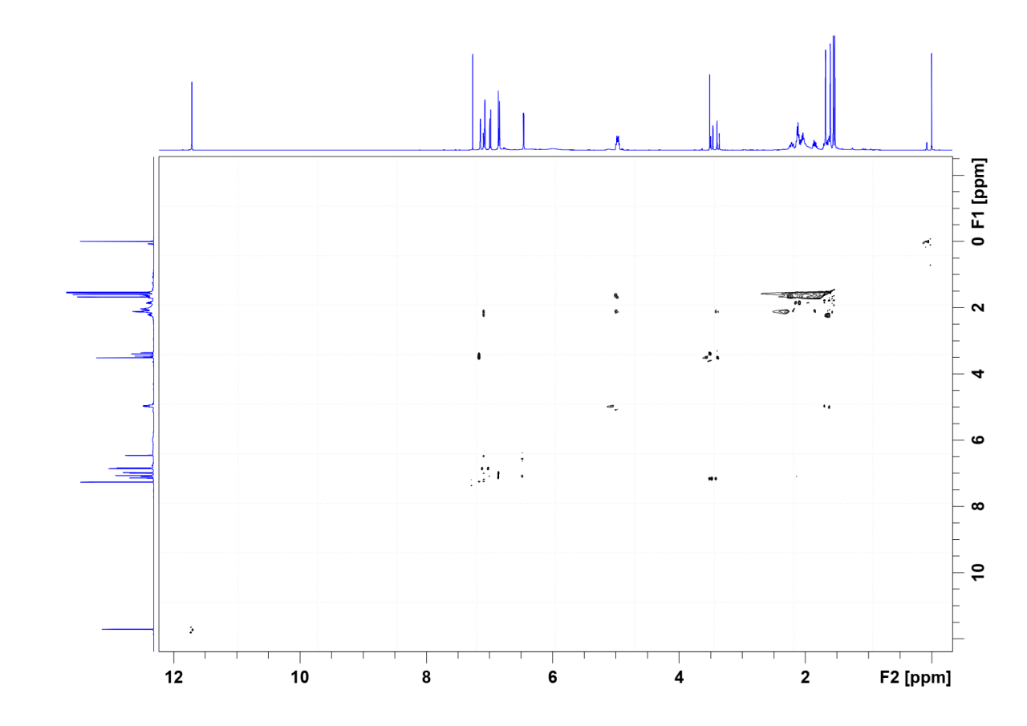


**Figure S84.** ROESY spectrum of compound **9** in CDCl_3_.


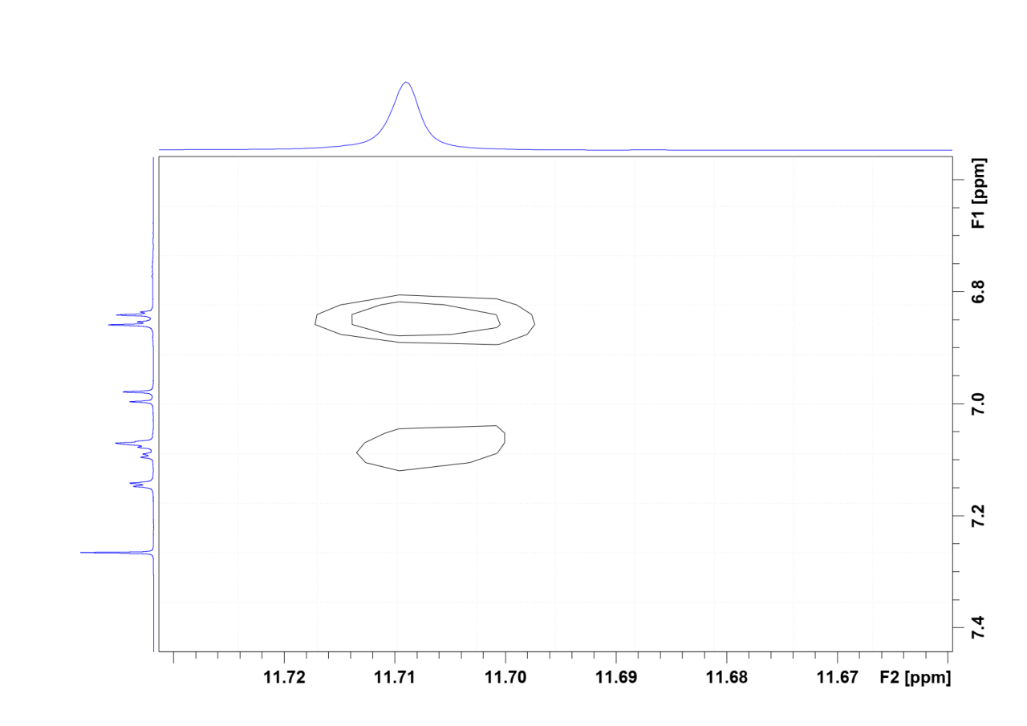


**Figure S85.** Enlarge ROESY spectrum of compound **9** in CDCl_3_.


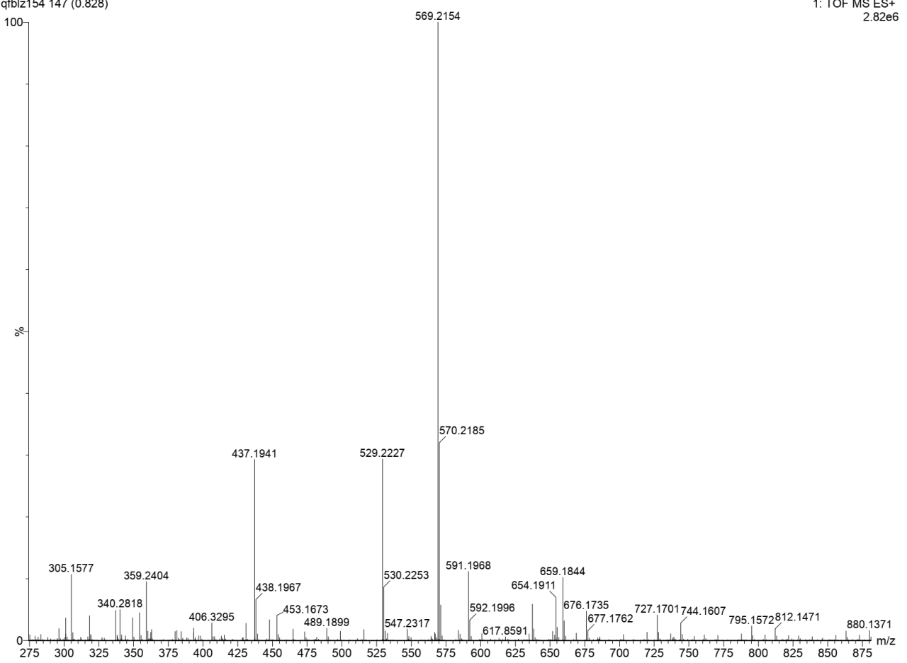


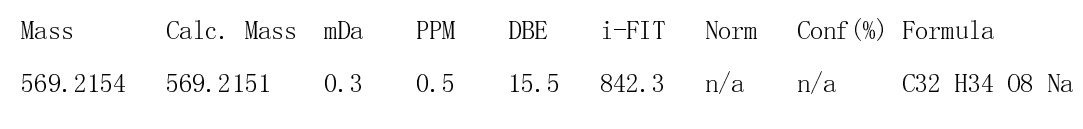


**Figure S86.** HRESIMS of **9**.

**Figure S87.** CD spectrum of (+)-**9**.

**Figure S88.** CD spectrum of (–)-**9**.


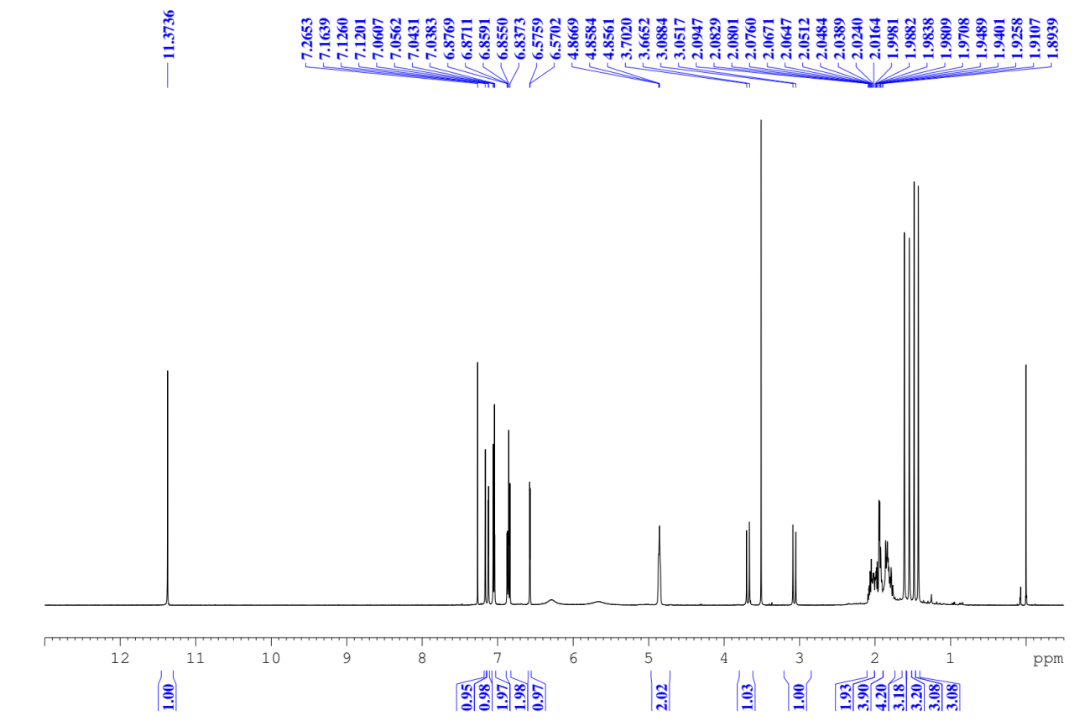


**Figure S89.** ^1^H NMR (500 MHz) spectrum of compound **10** in CDCl_3_.


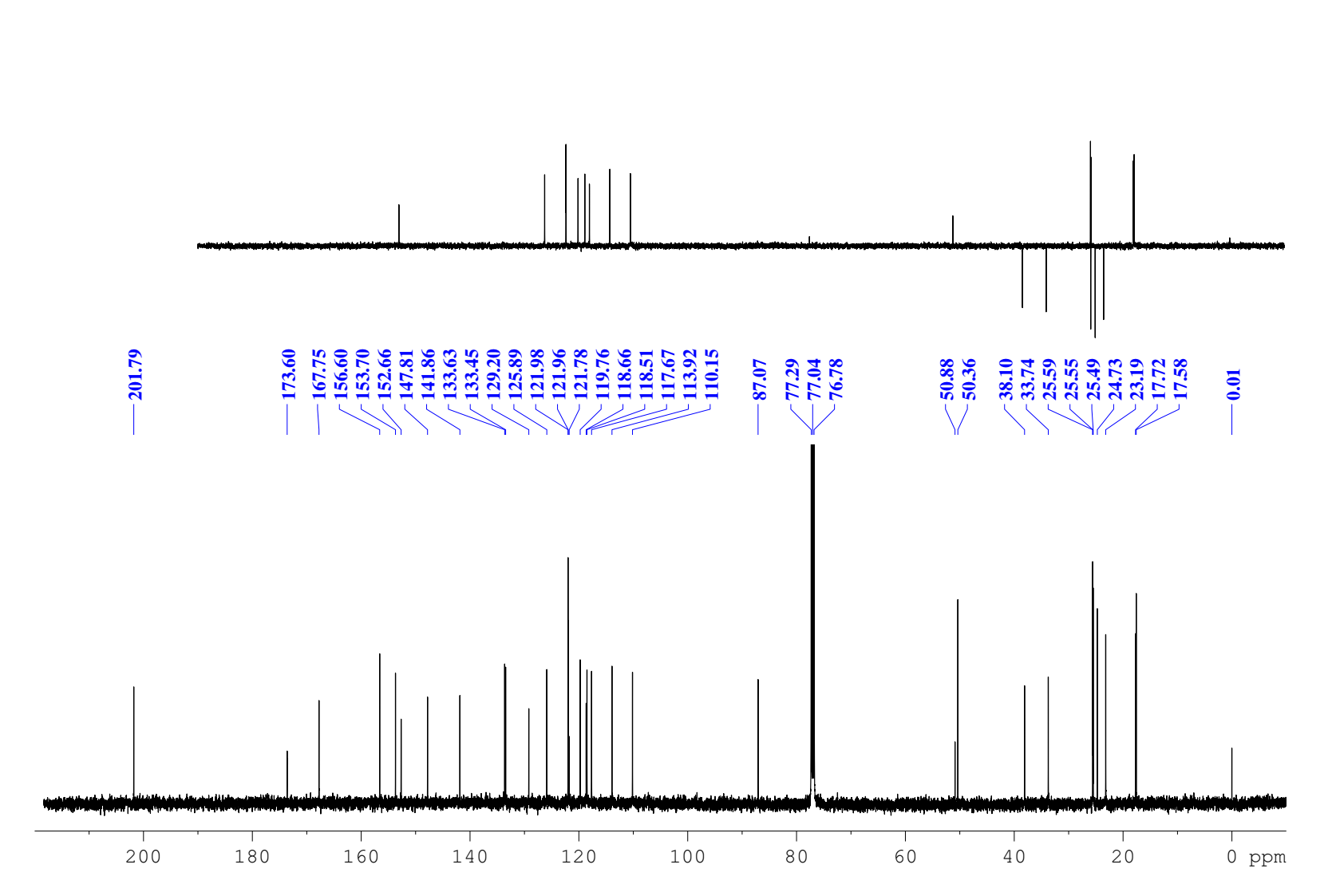


**Figure S90.** ^13^C NMR (125 MHz) spectrum of compound **10** in CDCl_3_.


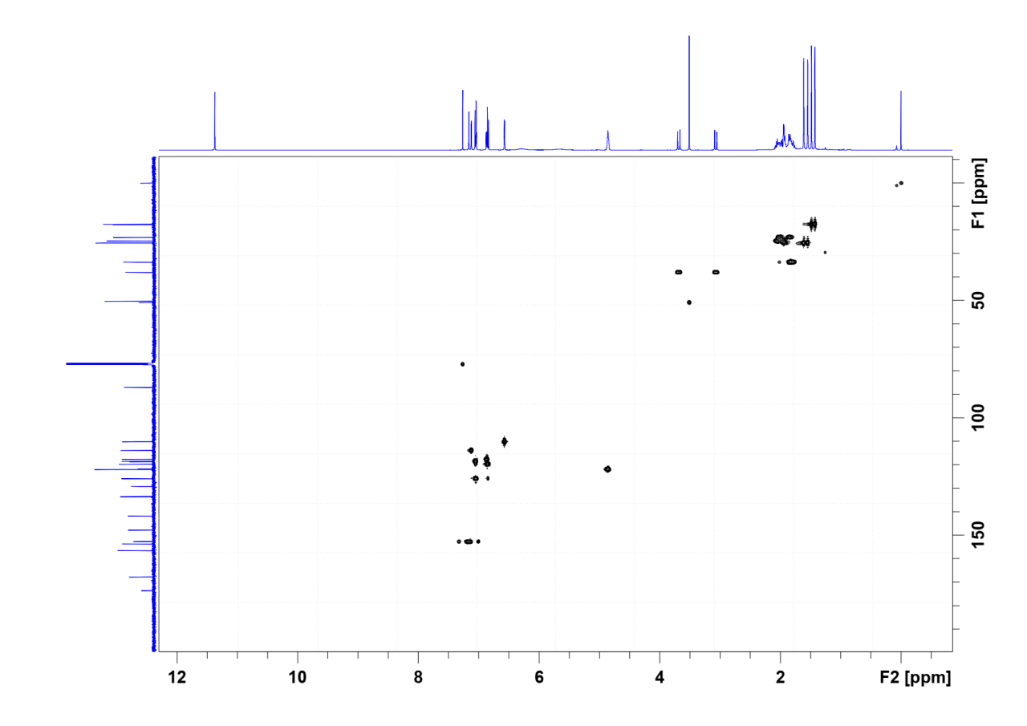


**Figure S91.** HSQC spectrum of compound **10** in CDCl_3_.


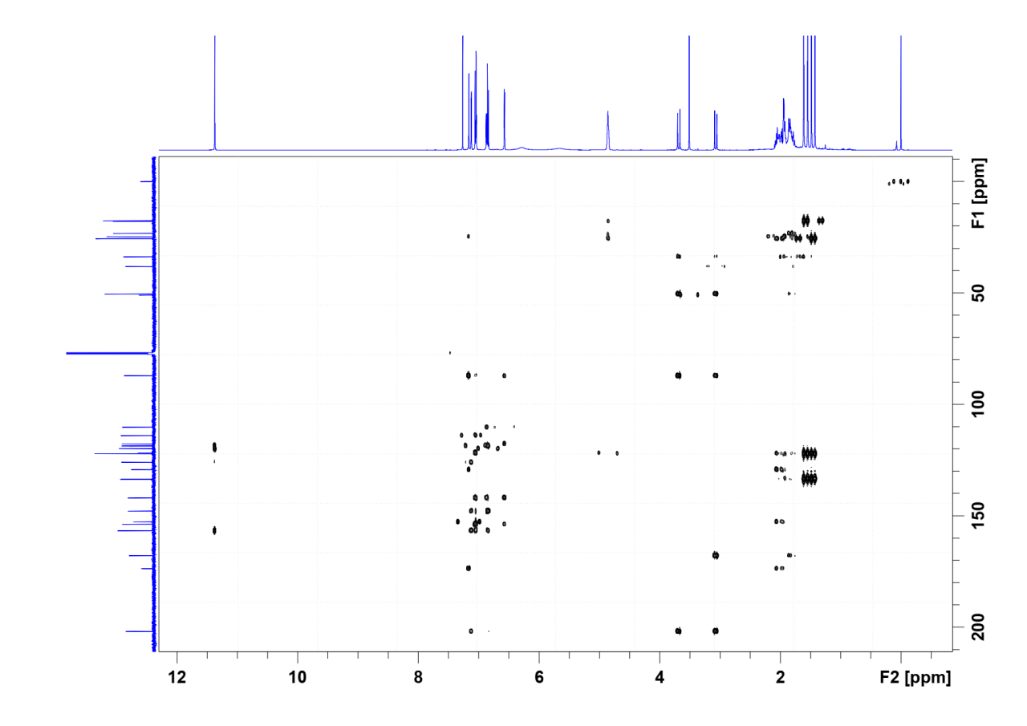


**Figure S92.** HMBC spectrum of compound **10** in CDCl_3_.


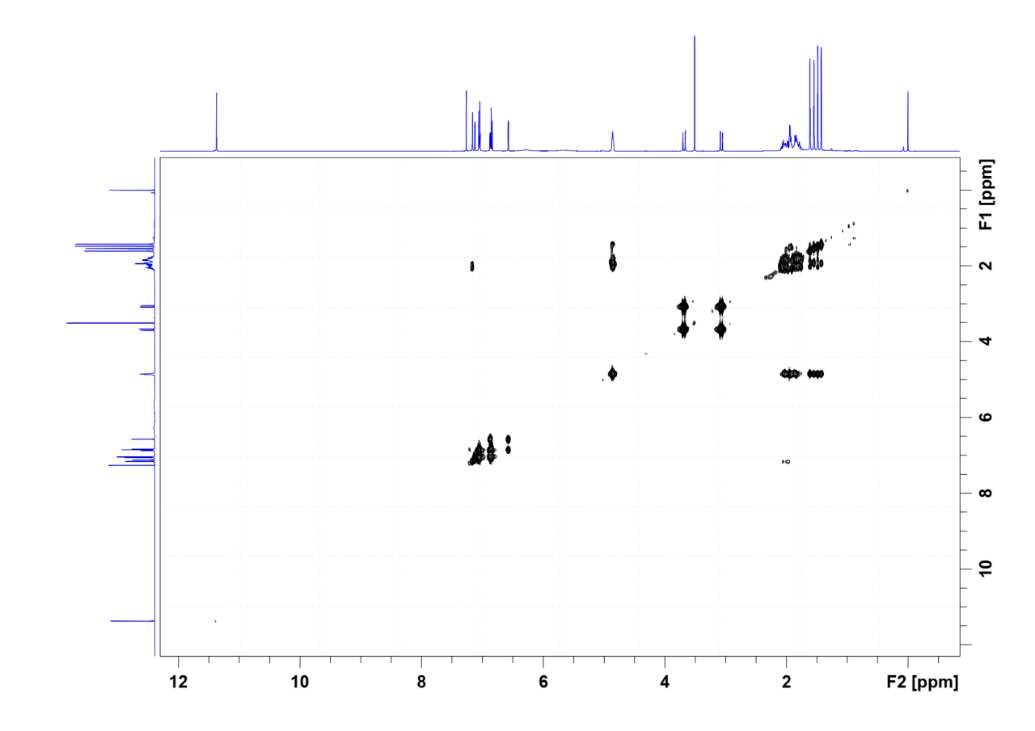


**Figure S93.** COSY spectrum of compound **10** in CDCl_3_.


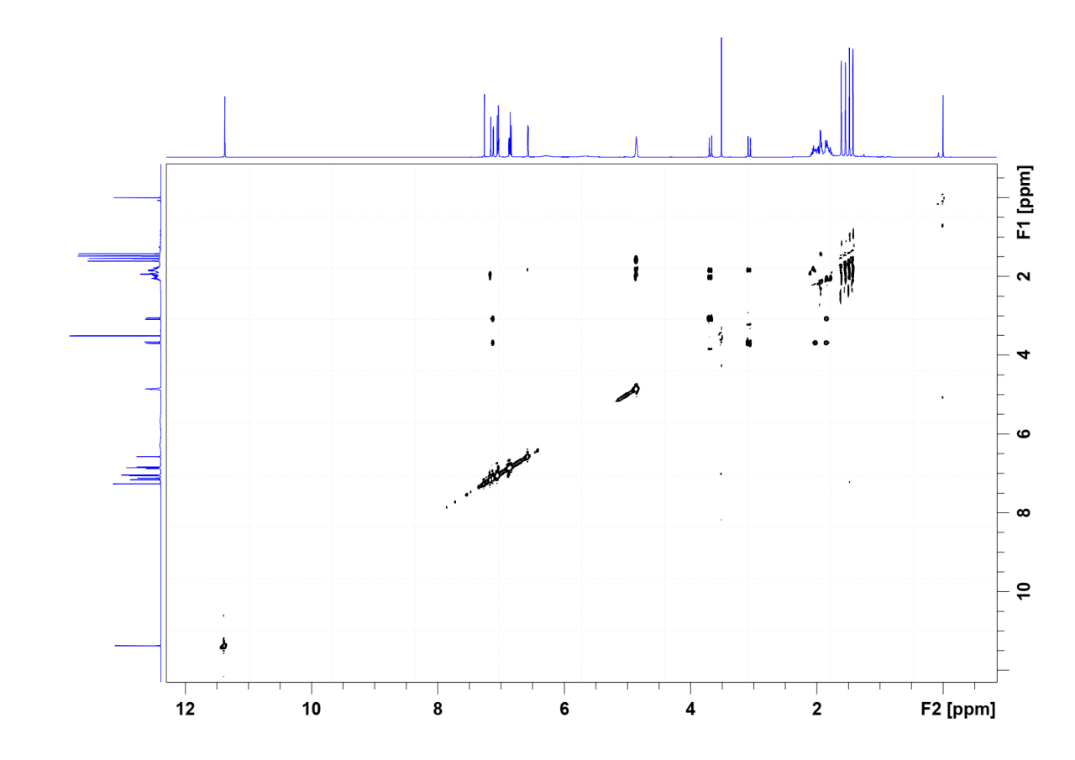


**Figure S94.** ROESY spectrum of compound **10** in CDCl_3_.


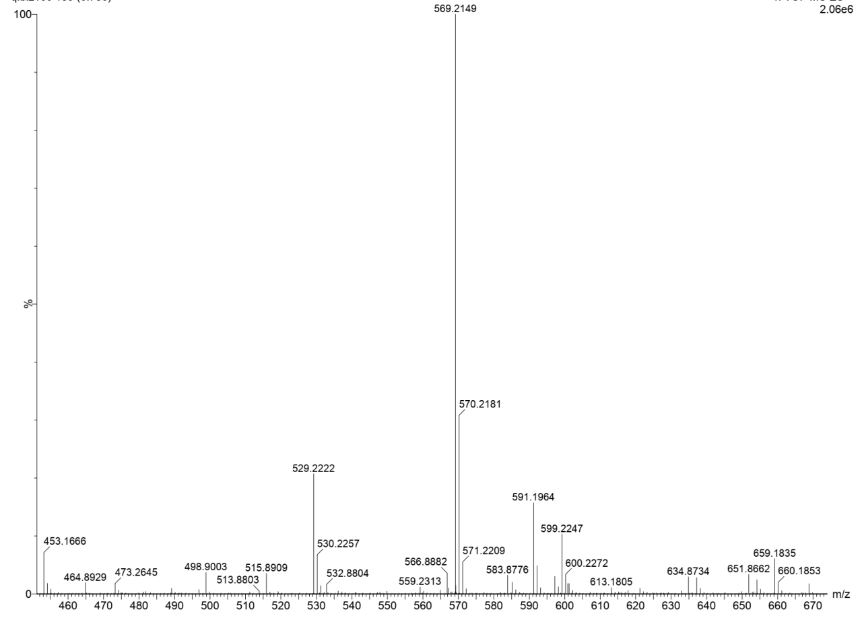


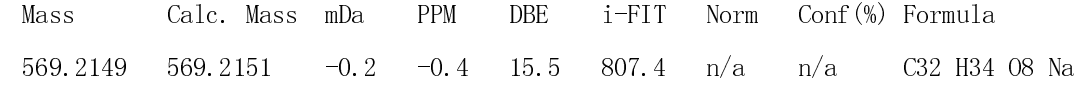


**Figure S95.** HRESIMS of **10**.

**Figure S96**. CD spectrum of (+)-**10**.

**Figure S97**. CD spectrum of (–)-**10**.


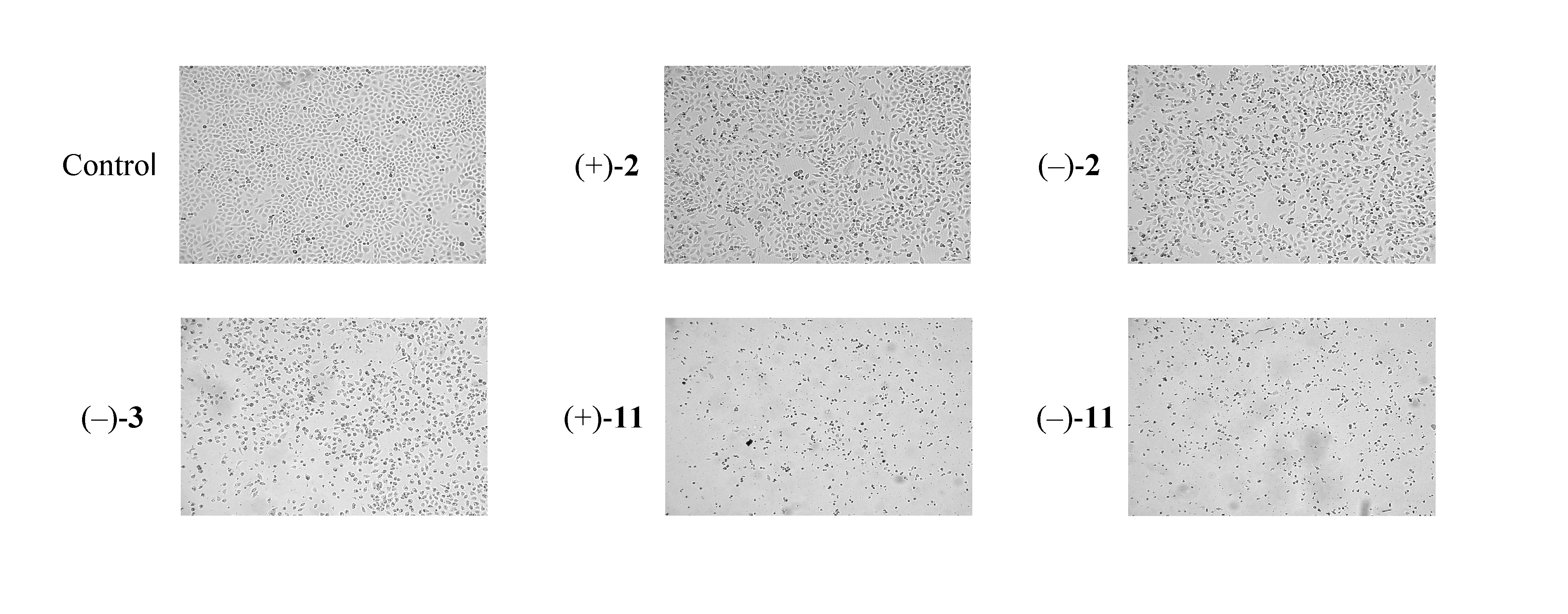


**Figure S98**. Representative images of the cell viability assay in MDA-MB-231 cells.

**ECD calculations**

The conformation search based on molecular mechanics with MMFF force fields. The low energy conformers were optimized at the B3LYP/6-31G(d,p) level using DFT/TDDFT method in the Gaussian 09 software package [1]. ECD calculations were conducted at the APFD/6-311+G(2d,p) level or B3LYP/6-31G(d,p) level in MeOH solution. The program SpecDis was used for comparison of the calculated curves and experimental CD spectra [2].

References

1. Frisch, M. J.; Trucks, G. W.; Schlegel, H. B.; Scuseria, G. E.; Robb, M. A.; Cheeseman, J. R.; Scalmani, G.; Barone, V.; Mennucci, B.; Petersson, G. A.; Nakatsuji, H.; Caricato, M.; Li, X.; Hratchian, H. P.; Izmaylov, A. F.; Bloino, J.; Zheng, G.; Sonnenberg, J. L.; Hada, M.; Ehara, M.; Toyota, K.; Fukuda, R.; Hasegawa, J.; Ishida, M.; Nakajima, T.; Honda, Y.; Kitao, O.; Nakai, H.; Vreven, T.; Montgomery, J. A.; Peralta, J. E.; Ogliaro, F.; Bearpark, M.; Heyd, J. J.; Brothers, E.; Kudin, K. N.; Staroverov, V. N.; Keith, T.; Kobayashi, R.; Normand, J.; Raghavachari, K.; Rendell, A.; Burant, J. C.; Iyengar, S. S.; Tomasi, J.; Cossi, M.; Rega, N.; Millam, J. M.; Klene, M.; Knox, J. E.; Cross, J. B.; Bakken, V.; Adamo, C.; Jaramillo, J.; Gomperts, R.; Stratmann, R. E.; Yazyev, O.; Austin, A. J.; Cammi, R.; Pomelli, C.; Ochterski, J. W.; Martin, R. L.; Morokuma, K.; Zakrzewski, V. G.; Voth, G. A.; Salvador, P.; Dannenberg, J. J.; Dapprich, S.; Daniels, A. D.; Farkas, O.; Foresman, J. B.; Ortiz, J. V.; Cioslowski, J.; Fox, D. J. Gaussian 09, revision C.01. Gaussian, Inc.: Wallingford CT, 2010.
2. Bruhn, T.; Schaumlöffel, A.; Hemberger, Y.; Bringmann, G. SpecDis: Quantifying the comparison of calculated and experimental electronic circular dichroism spectra. *Chirality.* **2013**, *25,* 243–249.
